# Global injury morbidity and mortality from 1990 to 2017: results from the Global Burden of Disease Study 2017

**DOI:** 10.1136/injuryprev-2019-043494

**Published:** 2020-04-24

**Authors:** Spencer L James, Chris D Castle, Zachary V Dingels, Jack T Fox, Erin B Hamilton, Zichen Liu, Nicholas L S Roberts, Dillon O Sylte, Nathaniel J Henry, Kate E LeGrand, Ahmed Abdelalim, Amir Abdoli, Ibrahim Abdollahpour, Rizwan Suliankatchi Abdulkader, Aidin Abedi, Akine Eshete Abosetugn, Abdelrahman I Abushouk, Oladimeji M Adebayo, Marcela Agudelo-Botero, Tauseef Ahmad, Rushdia Ahmed, Muktar Beshir Ahmed, Miloud Taki Eddine Aichour, Fares Alahdab, Genet Melak Alamene, Fahad Mashhour Alanezi, Animut Alebel, Niguse Meles Alema, Suliman A Alghnam, Samar Al-Hajj, Beriwan Abdulqadir Ali, Saqib Ali, Mahtab Alikhani, Cyrus Alinia, Vahid Alipour, Syed Mohamed Aljunid, Amir Almasi-Hashiani, Nihad A Almasri, Khalid Altirkawi, Yasser Sami Abdeldayem Amer, Saeed Amini, Arianna Maever Loreche Amit, Catalina Liliana Andrei, Alireza Ansari-Moghaddam, Carl Abelardo T Antonio, Seth Christopher Yaw Appiah, Jalal Arabloo, Morteza Arab-Zozani, Zohreh Arefi, Olatunde Aremu, Filippo Ariani, Amit Arora, Malke Asaad, Babak Asghari, Nefsu Awoke, Beatriz Paulina Ayala Quintanilla, Getinet Ayano, Martin Amogre Ayanore, Samad Azari, Ghasem Azarian, Alaa Badawi, Ashish D Badiye, Eleni Bagli, Atif Amin Baig, Mohan Bairwa, Ahad Bakhtiari, Arun Balachandran, Maciej Banach, Srikanta K Banerjee, Palash Chandra Banik, Amrit Banstola, Suzanne Lyn Barker-Collo, Till Winfried Bärnighausen, Lope H Barrero, Akbar Barzegar, Mohsen Bayati, Bayisa Abdissa Baye, Neeraj Bedi, Masoud Behzadifar, Tariku Tesfaye Bekuma, Habte Belete, Corina Benjet, Derrick A Bennett, Isabela M Bensenor, Kidanemaryam Berhe, Pankaj Bhardwaj, Anusha Ganapati Bhat, Krittika Bhattacharyya, Sadia Bibi, Ali Bijani, Muhammad Shahdaat Bin Sayeed, Guilherme Borges, Antonio Maria Borzì, Soufiane Boufous, Alexandra Brazinova, Nikolay Ivanovich Briko, Shyam S Budhathoki, Josip Car, Rosario Cárdenas, Félix Carvalho, João Mauricio Castaldelli-Maia, Carlos A Castañeda-Orjuela, Giulio Castelpietra, Ferrán Catalá-López, Ester Cerin, Joht S Chandan, Wagaye Fentahun Chanie, Soosanna Kumary Chattu, Vijay Kumar Chattu, Irini Chatziralli, Neha Chaudhary, Daniel Youngwhan Cho, Mohiuddin Ahsanul Kabir Chowdhury, Dinh-Toi Chu, Samantha M Colquhoun, Maria-Magdalena Constantin, Vera M Costa, Giovanni Damiani, Ahmad Daryani, Claudio Alberto Dávila-Cervantes, Feleke Mekonnen Demeke, Asmamaw Bizuneh Demis, Gebre Teklemariam Demoz, Desalegn Getnet Demsie, Afshin Derakhshani, Kebede Deribe, Rupak Desai, Mostafa Dianati Nasab, Diana Dias da Silva, Zahra Sadat Dibaji Forooshani, Kerrie E Doyle, Tim Robert Driscoll, Eleonora Dubljanin, Bereket Duko Adema, Arielle Wilder Eagan, Aziz Eftekhari, Elham Ehsani-Chimeh, Maysaa El Sayed Zaki, Demelash Abewa Elemineh, Shaimaa I El-Jaafary, Ziad El-Khatib, Christian Lycke Ellingsen, Mohammad Hassan Emamian, Daniel Adane Endalew, Sharareh Eskandarieh, Pawan Sirwan Faris, Andre Faro, Farshad Farzadfar, Yousef Fatahi, Wubalem Fekadu, Tomas Y Ferede, Seyed-Mohammad Fereshtehnejad, Eduarda Fernandes, Pietro Ferrara, Garumma Tolu Feyissa, Irina Filip, Florian Fischer, Morenike Oluwatoyin Folayan, Masoud Foroutan, Joel Msafiri Francis, Richard Charles Franklin, Takeshi Fukumoto, Biniyam Sahiledengle Geberemariyam, Abadi Kahsu Gebre, Ketema Bizuwork Gebremedhin, Gebreamlak Gebremedhn Gebremeskel, Berhe Gebremichael, Getnet Azeze Gedefaw, Birhanu Geta, Mansour Ghafourifard, Farhad Ghamari, Ahmad Ghashghaee, Asadollah Gholamian, Tiffany K Gill, Alessandra C Goulart, Ayman Grada, Michal Grivna, Mohammed Ibrahim Mohialdeen Gubari, Rafael Alves Guimarães, Yuming Guo, Gaurav Gupta, Juanita A Haagsma, Nima Hafezi-Nejad, Hassan Haghparast Bidgoli, Brian James Hall, Randah R Hamadeh, Samer Hamidi, Josep Maria Haro, Md Mehedi Hasan, Amir Hasanzadeh, Soheil Hassanipour, Hadi Hassankhani, Hamid Yimam Hassen, Rasmus Havmoeller, Khezar Hayat, Delia Hendrie, Fatemeh Heydarpour, Martha Híjar, Hung Chak Ho, Chi Linh Hoang, Michael K Hole, Ramesh Holla, Naznin Hossain, Mehdi Hosseinzadeh, Sorin Hostiuc, Guoqing Hu, Segun Emmanuel Ibitoye, Olayinka Stephen Ilesanmi, Irena Ilic, Milena D Ilic, Leeberk Raja Inbaraj, Endang Indriasih, Seyed Sina Naghibi Irvani, Sheikh Mohammed Shariful Islam, M Mofizul Islam, Rebecca Q Ivers, Kathryn H Jacobsen, Mohammad Ali Jahani, Nader Jahanmehr, Mihajlo Jakovljevic, Farzad Jalilian, Sudha Jayaraman, Achala Upendra Jayatilleke, Ravi Prakash Jha, Yetunde O John-Akinola, Jost B Jonas, Nitin Joseph, Farahnaz Joukar, Jacek Jerzy Jozwiak, Suresh Banayya Jungari, Mikk Jürisson, Ali Kabir, Rajendra Kadel, Amaha Kahsay, Leila R Kalankesh, Rohollah Kalhor, Teshome Abegaz Kamil, Tanuj Kanchan, Neeti Kapoor, Manoochehr Karami, Amir Kasaeian, Hagazi Gebremedhin Kassaye, Taras Kavetskyy, Hafte Kahsay Kebede, Peter Njenga Keiyoro, Abraham Getachew Kelbore, Bayew Kelkay, Yousef Saleh Khader, Morteza Abdullatif Khafaie, Nauman Khalid, Ibrahim A Khalil, Rovshan Khalilov, Mohammad Khammarnia, Ejaz Ahmad Khan, Maseer Khan, Tripti Khanna, Habibolah Khazaie, Fatemeh Khosravi Shadmani, Roba Khundkar, Daniel N Kiirithio, Young-Eun Kim, Daniel Kim, Yun Jin Kim, Adnan Kisa, Sezer Kisa, Hamidreza Komaki, Shivakumar K M Kondlahalli, Vladimir Andreevich Korshunov, Ai Koyanagi, Moritz U G Kraemer, Kewal Krishan, Burcu Kucuk Bicer, Nuworza Kugbey, Vivek Kumar, Nithin Kumar, G Anil Kumar, Manasi Kumar, Girikumar Kumaresh, Om P Kurmi, Oluwatosin Kuti, Carlo La Vecchia, Faris Hasan Lami, Prabhat Lamichhane, Justin J Lang, Van C Lansingh, Dennis Odai Laryea, Savita Lasrado, Arman Latifi, Paolo Lauriola, Janet L Leasher, Shaun Wen Huey Lee, Tsegaye Lolaso Lenjebo, Miriam Levi, Shanshan Li, Shai Linn, Xuefeng Liu, Alan D Lopez, Paulo A Lotufo, Raimundas Lunevicius, Ronan A Lyons, Mohammed Madadin, Muhammed Magdy Abd El Razek, Narayan Bahadur Mahotra, Marek Majdan, Azeem Majeed, Jeadran N Malagon-Rojas, Venkatesh Maled, Reza Malekzadeh, Deborah Carvalho Malta, Navid Manafi, Amir Manafi, Ana-Laura Manda, Narayana Manjunatha, Fariborz Mansour-Ghanaei, Borhan Mansouri, Mohammad Ali Mansournia, Joemer C Maravilla, Lyn M March, Amanda J Mason-Jones, Seyedeh Zahra Masoumi, Benjamin Ballard Massenburg, Pallab K Maulik, Gebrekiros Gebremichael Meles, Addisu Melese, Zeleke Aschalew Melketsedik, Peter T N Memiah, Walter Mendoza, Ritesh G Menezes, Meresa Berwo Mengesha, Melkamu Merid Mengesha, Tuomo J Meretoja, Atte Meretoja, Hayimro Edemealem Merie, Tomislav Mestrovic, Bartosz Miazgowski, Tomasz Miazgowski, Ted R Miller, GK Mini, Andreea Mirica, Erkin M Mirrakhimov, Mehdi Mirzaei-Alavijeh, Prasanna Mithra, Babak Moazen, Masoud Moghadaszadeh, Efat Mohamadi, Yousef Mohammad, Karzan Abdulmuhsin Mohammad, Aso Mohammad Darwesh, Naser Mohammad Gholi Mezerji, Abdollah Mohammadian-Hafshejani, Milad Mohammadoo-Khorasani, Reza Mohammadpourhodki, Shafiu Mohammed, Jemal Abdu Mohammed, Farnam Mohebi, Mariam Molokhia, Lorenzo Monasta, Yoshan Moodley, Mahmood Moosazadeh, Masoud Moradi, Ghobad Moradi, Maziar Moradi-Lakeh, Farhad Moradpour, Lidia Morawska, Ilais Moreno Velásquez, Naho Morisaki, Shane Douglas Morrison, Tilahun Belete Mossie, Atalay Goshu Muluneh, Srinivas Murthy, Kamarul Imran Musa, Ghulam Mustafa, Ashraf F Nabhan, Ahamarshan Jayaraman Nagarajan, Gurudatta Naik, Mukhammad David Naimzada, Farid Najafi, Vinay Nangia, Bruno Ramos Nascimento, Morteza Naserbakht, Vinod Nayak, Duduzile Edith Ndwandwe, Ionut Negoi, Josephine W Ngunjiri, Cuong Tat Nguyen, Huong Lan Thi Nguyen, Rajan Nikbakhsh, Dina Nur Anggraini Ningrum, Chukwudi A Nnaji, Peter S Nyasulu, Felix Akpojene Ogbo, Onome Bright Oghenetega, In-Hwan Oh, Emmanuel Wandera Okunga, Andrew T Olagunju, Tinuke O Olagunju, Ahmed Omar Bali, Obinna E Onwujekwe, Kwaku Oppong Asante, Heather M Orpana, Erika Ota, Nikita Otstavnov, Stanislav S Otstavnov, Mahesh P A, Jagadish Rao Padubidri, Smita Pakhale, Keyvan Pakshir, Songhomitra Panda-Jonas, Eun-Kee Park, Sangram Kishor Patel, Ashish Pathak, Sanghamitra Pati, George C Patton, Kebreab Paulos, Amy E Peden, Veincent Christian Filipino Pepito, Jeevan Pereira, Hai Quang Pham, Michael R Phillips, Marina Pinheiro, Roman V Polibin, Suzanne Polinder, Hossein Poustchi, Swayam Prakash, Dimas Ria Angga Pribadi, Parul Puri, Zahiruddin Quazi Syed, Mohammad Rabiee, Navid Rabiee, Amir Radfar, Anwar Rafay, Ata Rafiee, Alireza Rafiei, Fakher Rahim, Siavash Rahimi, Vafa Rahimi-Movaghar, Muhammad Aziz Rahman, Ali Rajabpour-Sanati, Fatemeh Rajati, Ivo Rakovac, Kavitha Ranganathan, Sowmya J Rao, Vahid Rashedi, Prateek Rastogi, Priya Rathi, Salman Rawaf, Lal Rawal, Reza Rawassizadeh, Vishnu Renjith, Andre M N Renzaho, Serge Resnikoff, Aziz Rezapour, Ana Isabel Ribeiro, Jennifer Rickard, Carlos Miguel Rios González, Luca Ronfani, Gholamreza Roshandel, Anas M Saad, Yogesh Damodar Sabde, Siamak Sabour, Basema Saddik, Saeed Safari, Roya Safari-Faramani, Hamid Safarpour, Mahdi Safdarian, S Mohammad Sajadi, Payman Salamati, Farkhonde Salehi, Saleh Salehi Zahabi, Marwa R Rashad Salem, Hosni Salem, Omar Salman, Inbal Salz, Abdallah M Samy, Juan Sanabria, Lidia Sanchez Riera, Milena M Santric Milicevic, Abdur Razzaque Sarker, Arash Sarveazad, Brijesh Sathian, Monika Sawhney, Susan M Sawyer, Sonia Saxena, Mehdi Sayyah, David C Schwebel, Soraya Seedat, Subramanian Senthilkumaran, Sadaf G Sepanlou, Seyedmojtaba Seyedmousavi, Feng Sha, Faramarz Shaahmadi, Saeed Shahabi, Masood Ali Shaikh, Mehran Shams-Beyranvand, Morteza Shamsizadeh, Mahdi Sharif-Alhoseini, Hamid Sharifi, Aziz Sheikh, Mika Shigematsu, Jae Il Shin, Rahman Shiri, Soraya Siabani, Inga Dora Sigfusdottir, Pankaj Kumar Singh, Jasvinder A Singh, Dhirendra Narain Sinha, Catalin-Gabriel Smarandache, Emma U R Smith, Amin Soheili, Bija Soleymani, Ali Reza Soltanian, Joan B Soriano, Muluken Bekele Sorrie, Ireneous N Soyiri, Dan J Stein, Mark A Stokes, Mu'awiyyah Babale Sufiyan, Hafiz Ansar Rasul Suleria, Bryan L Sykes, Rafael Tabarés-Seisdedos, Karen M Tabb, Biruk Wogayehu Taddele, Degena Bahrey Tadesse, Animut Tagele Tamiru, Ingan Ukur Tarigan, Yonatal Mesfin Tefera, Arash Tehrani-Banihashemi, Merhawi Gebremedhin Tekle, Gebretsadkan Hintsa Tekulu, Ayenew Kassie Tesema, Berhe Etsay Tesfay, Rekha Thapar, Asres Bedaso Tilahune, Kenean Getaneh Tlaye, Hamid Reza Tohidinik, Roman Topor-Madry, Bach Xuan Tran, Khanh Bao Tran, Jaya Prasad Tripathy, Alexander C Tsai, Lorainne Tudor Car, Saif Ullah, Irfan Ullah, Maida Umar, Bhaskaran Unnikrishnan, Era Upadhyay, Olalekan A Uthman, Pascual R Valdez, Tommi Juhani Vasankari, Narayanaswamy Venketasubramanian, Francesco S Violante, Vasily Vlassov, Yasir Waheed, Girmay Teklay Weldesamuel, Andrea Werdecker, Taweewat Wiangkham, Haileab Fekadu Wolde, Dawit Habte Woldeyes, Dawit Zewdu Wondafrash, Temesgen Gebeyehu Wondmeneh, Adam Belay Wondmieneh, Ai-Min Wu, Rajaram Yadav, Ali Yadollahpour, Yuichiro Yano, Sanni Yaya, Vahid Yazdi-Feyzabadi, Paul Yip, Engida Yisma, Naohiro Yonemoto, Seok-Jun Yoon, Yoosik Youm, Mustafa Z Younis, Zabihollah Yousefi, Yong Yu, Chuanhua Yu, Hasan Yusefzadeh, Telma Zahirian Moghadam, Zoubida Zaidi, Sojib Bin Zaman, Mohammad Zamani, Maryam Zamanian, Hamed Zandian, Ahmad Zarei, Fatemeh Zare, Zhi-Jiang Zhang, Yunquan Zhang, Sanjay Zodpey, Lalit Dandona, Rakhi Dandona, Louisa Degenhardt, Samath Dhamminda Dharmaratne, Simon I Hay, Ali H Mokdad, Robert C Reiner, Benn Sartorius, Theo Vos

**Affiliations:** 1 Institute for Health Metrics and Evaluation, University of Washington, Seattle, WA, USA; 2 Department of Neurology, Cairo University, Cairo, Egypt; 3 Department of Parasitology and Mycology, Jahrom University of Medical Sciences, Jahrom, Iran; 4 Neuroscience Research Center, Isfahan University of Medical Sciences, Isfahan, Iran; 5 Department of Public Health, Ministry of Health, Riyadh, Saudi Arabia; 6 Department of Orthopaedic Surgery, University of Southern California, Los Angeles, CA, USA; 7 Department of Public Health, Debre Berhan University, Debre Berhan, Ethiopia; 8 Cardiovascular Medicine, Ain Shams University, Abbasia, Egypt; 9 Department of Medicine, University College Hospital, Ibadan, Ibadan, Nigeria; 10 School of Medicine Center for Politics, Population and Health Research, National Autonomous University of Mexico, Mexico City, Mexico; 11 Department of Epidemiology and Health Statistics, Southeast University Nanjing, Nanjing, China; 12 Department of Microbiology, Hazara University Mansehra, Mansehra, Pakistan; 13 James P Grant School of Public Health, BRAC University, Dhaka, Bangladesh; 14 Health Systems and Population Studies Division, International Centre for Diarrhoeal Disease Research, Bangladesh, Dhaka, Bangladesh; 15 Department of Epidemiology, Jimma University, Jimma, Ethiopia; 16 Higher National School of Veterinary Medicine, Algiers, Algeria; 17 Evidence Based Practice Center, Mayo Clinic Foundation for Medical Education and Research, Rochester, MN, USA; 18 School of Health Sciences, Madda Walabu University, Bale Goba, Ethiopia; 19 Department of Computer Sciences, Imam Abdulrehman Bin Faisal University, Dammam, Saudi Arabia; 20 Department of Nursing, Debre Markos University, Debre Markos, Ethiopia; 21 Department of Pharmacy, Adigrat University, Adigrat, Ethiopia; 22 Department of Population Health Research, King Abdullah International Medical Research Center, Riyadh, Saudi Arabia; 23 Faculty of Health Sciences - Health Management and Policy, American University of Beirut, Beirut, Lebanon; 24 British Columbia Injury Research Prevention Unit, British Columbia Children’s Hospital Research Institute, Vancouver, BC, Canada; 25 Medical Technical Institute, Erbil Polytechnic University, Erbil, Iraq; 26 Faculty of Pharmacy, Ishik University, Erbil, Iraq; 27 Department of Information Systems, College of Economics and Political Science, Sultan Qaboos University, Muscat, Oman; 28 School of Health Management and Information Sciences, Department of Health Services Management, Iran University of Medical Sciences, Tehran, Iran; 29 Department of Health Care Management and Economics, Urmia University of Medical Science, Urmia, Iran; 30 Health Management and Economics Research Center, Iran University of Medical Sciences, Tehran, Iran; 31 Health Economics Department, Iran University of Medical Sciences, Tehran, Iran; 32 Department of Health Policy and Management, Kuwait University, Safat, Kuwait; 33 International Centre for Casemix and Clinical Coding, National University of Malaysia, Bandar Tun Razak, Malaysia; 34 Department of Epidemiology, Arak University of Medical Sciences, Arak, Iran; 35 Physiotherapy Department, The University of Jordan, Amman, Jordan; 36 King Saud University, Riyadh, Saudi Arabia; 37 Clinical Practice Guidelines Unit, King Saud University, Riyadh, Saudi Arabia; 38 Alexandria Center for Evidence-Based Clinical Practice Guidelines, Alexandria University, Alexandria, Egypt; 39 Health Services Management Department, Arak University of Medical Sciences, Arak, Iran; 40 Department of Epidemiology and Biostatistics, University of the Philippines Manila, Manila, Philippines; 41 Online Programs for Applied Learning, Johns Hopkins University, Baltimore, MD, USA; 42 Carol Davila University of Medicine and Pharmacy, Bucharest, Romania; 43 Department of Epidemiology and Biostatistics, Health Promotion Research Center, Zahedan, Iran; 44 Department of Health Policy and Administration, University of the Philippines Manila, Manila, Philippines; 45 Department of Applied Social Sciences, Hong Kong Polytechnic University, Hong Kong, China; 46 Department of Sociology and Social Work, Kwame Nkrumah University of Science and Technology, Kumasi, Ghana; 47 Center for International Health, Ludwig Maximilians University, Munich, Germany; 48 Social Determinants of Health Research Center, Birjand University of Medical Sciences, Birjand, Iran; 49 Department of Health Promotion and Education, Tehran University of Medical Sciences, Tehran, Iran; 50 School of Health Sciences, Birmingham City University, Birmingham, UK; 51 Regional Centre for the Analysis of Data on Occupational and Work-related Injuries and Diseases, Local Health Unit Tuscany Centre, Florence, Italy; 52 School of Science and Health, Western Sydney University, Sydney, New South Wales, Australia; 53 Oral Health Services, Sydney Local Health District, Sydney, New South Wales, Australia; 54 Department of Plastic Surgery, University of Texas, Houston, TX, USA; 55 Department of Microbiology, Hamedan University of Medical Sciences, Azad Tabriz University, Iran; 56 Department of Nursing, Wolaita Sodo University, Wolaita Sodo, Ethiopia; 57 The Judith Lumley Centre, La Trobe University, Melbourne, VIC, Australia; 58 General Office for Research and Technological Transfer, Peruvian National Institute of Health, Lima, Peru; 59 School of Public Health, Curtin University, Perth, WA, Australia; 60 Department of Health Policy Planning and Management, University of Health and Allied Sciences, Ho, Ghana; 61 Department of Environmental Health Engineering, Hamadan University of Medical Sciences, Hamadan, Iran; 62 Public Health Risk Sciences Division, Public Health Agency of Canada, Toronto, Ontario, Canada; 63 Department of Nutritional Sciences, University of Toronto, Toronto, Ontario, Canada; 64 Department of Forensic Science, Government Institute of Forensic Science, Nagpur, India; 65 Department of Ophthalmology, University Hospital of Ioannina, Ioannina, Greece; 66 Institute of Molecular Biology & Biotechnology, Foundation for Research & Technology, Ioannina, Greece; 67 Biochemistry Unit, Universiti Sultan Zainal Abidin, Kuala Terengganu, Malaysia; 68 School of Health Sciences, Univeristi Sultan Zainal Abidin, Kuala Terengganu, Malaysia; 69 Institute of Health Management Research, Indian Institute of Health Management Research University, Jaipur, India; 70 Department of Epidemiology, Johns Hopkins University, Baltimore, MD, USA; 71 Health Policy And Management Department, Tehran University of Medical Sciences, Tehran, Iran; 72 Department of Demography, University of Groningen, Groningen, Netherlands; 73 Population Research Centre, Institute for Social and Economic Change, Bengaluru, India; 74 Department of Hypertension, Medical University of Lodz, Lodz, Poland; 75 Polish Mothers’ Memorial Hospital Research Institute, Lodz, Poland; 76 School of Health Sciences, Walden University, Minneapolis, MN, USA; 77 Department of Noncommunicable Diseases, Bangladesh University of Health Sciences (BUHS), Dhaka, Bangladesh; 78 Department of Research, Public Health Perspective Nepal, Pokhara-Lekhnath Metropolitan City, Nepal; 79 School of Psychology, University of Auckland, Auckland, New Zealand; 80 Heidelberg Institute of Global Health (HIGH), Heidelberg University, Heidelberg, Germany; 81 T H Chan School of Public Health, Harvard University, Boston, MA, USA; 82 Department of Industrial Engineering, Pontifical Javeriana University, Bogota, Colombia; 83 Occupational Health Department, Kermanshah University of Medical Sciences, Kermanshah, Iran; 84 Health Human Resources Research Center, Shiraz University of Medical Sciences, Shiraz, Iran; 85 Department of Public Health, Ambo University, Ambo, Ethiopia; 86 Department of Community Medicine, Gandhi Medical College Bhopal, Bhopal, India; 87 Jazan University, Jazan, Saudi Arabia; 88 Social Determinants of Health Research Center, Lorestan University of Medical Sciences, Khorramabad, Iran; 89 Institute of Health Sciences, School of Public Health, Wollega University, Nekemte, Ethiopia; 90 Department of Psychiatry, Bahir Dar University, Bahir Dar, Ethiopia; 91 Department of Epidemiology and Psychosocial Reseach, Ramón de la Fuente Muñiz National Institute of Psychiatry, Mexico City, Mexico; 92 Nuffield Department of Population Health, University of Oxford, Oxford, UK; 93 Department of Internal Medicine, University of São Paulo, São Paulo, Brazil; 94 Department of Nutrition and Dietetics, Mekelle University, Mekelle, Ethiopia; 95 Department of Community Medicine and Family Medicine, All India Institute of Medical Sciences, Jodhpur, India; 96 Department of Community Medicine, Datta Meghe Institute of Medical Sciences, Wardha, India; 97 Department of Internal Medicine, University of Massachusetts Medical School, Springfield, MA, USA; 98 Department of Statistical and Computational Genomics, National Institute of Biomedical Genomics, Kalyani, India; 99 Department of Statistics, University of Calcutta, Kolkata, India; 100 Institute of Soil and Environmental Sciences, University of Agriculture, Faisalabad, Faisalabad, Pakistan; 101 Social Determinants of Health Research Center, Babol University of Medical Sciences, Babol, Iran; 102 National Centre for Epidemiology and Population Health, Australian National University, Canberra, ACT, Australia; 103 Department of Clinical Pharmacy and Pharmacology, University of Dhaka, Dhaka, Bangladesh; 104 Department of Clinical and Experimental Medicine, University of Catania, Catania, Italy; 105 Transport and Road Safety (TARS) Research Department, University of New South Wales, Sydney, New South Wales, Australia; 106 Institute of Epidemiology, Comenius University, Bratislava, Slovakia; 107 Department of Epidemiology and Evidence Based Medicine, I M Sechenov First Moscow State Medical University, Moscow, Russia; 108 Research Department, Golden Community, Kathmandu, Nepal; 109 Centre for Population Health Sciences, Nanyang Technological University, Singapore; 110 Global eHealth Unit, Imperial College London, London, UK; 111 Department of Population and Health, Metropolitan Autonomous University, Mexico City, Mexico; 112 Research Unit on Applied Molecular Biosciences (UCIBIO), University of Porto, Porto, Portugal; 113 Department of Psychiatry, University of São Paulo, São Paulo, Brazil; 114 Colombian National Health Observatory, National Institute of Health, Bogota, Colombia; 115 Epidemiology and Public Health Evaluation Group, National University of Colombia, Bogota, Colombia; 116 Primary Care Services Area, Central Health Directorate, Region Friuli Venezia Giulia, Trieste, Italy; 117 Department of Medicine (DAME), University of Udine, Udine, Italy; 118 National School of Public Health, Carlos III Health Institute, Madrid, Spain; 119 Clinical Epidemiology Program, Ottawa Hospital Research Institute, Ottawa, ON, Canada; 120 Mary MacKillop Institute for Health Research, Australian Catholic University, Melbourne, VIC, Australia; 121 School of Public Health, University of Hong Kong, Hong Kong, China; 122 Institute of Applied Health Research, University of Birmingham, Birmingham, UK; 123 Department of Gynecology and Obstetrics, University of Gondar, Gondar, Ethiopia; 124 Department of Public Health, Texila American University, Georgetown, Guyana; 125 Department of Medicine, University of Toronto, Toronto, Canada; 126 2nd Department of Ophthalmology, University of Athens, Haidari, Greece; 127 Ophthalmology Private Practice Office, Independent Consultant, Athens, Greece; 128 Department of Pediatrics, Harvard University, Boston, MA, USA; 129 Department of Neonatology, Beth Israel Deaconess Medical center, Boston, MA, USA; 130 Department of Surgery, Division of Plastic and Reconstructive Surgery, University of Washington, Seattle, WA, USA; 131 Maternal and Child Health Division, International Centre for Diarrhoeal Disease Research, Bangladesh, Dhaka, Bangladesh; 132 Department of Epidemiology and Biostatistics, University of South Carolina, Columbia, SC, USA; 133 Faculty of Biology, Hanoi National University of Education, Hanoi, Vietnam; 134 Research School of Population Health, Australian National University, Action, ACT, Australia; 135 Department of Dermatology, Carol Davila University of Medicine and Pharmacy, Bucharest, Romania; 136 2nd Department of Dermatology, Colentina Clinical Hospital, Bucharest, Romania; 137 Department of Dermatology, Case Western Reserve University, Cleveland, OH, USA; 138 Department of Dermatology, University of Milan, Milan, Italy; 139 Toxoplasmosis Research Center, Mazandaran University of Medical Sciences, Sari, Iran; 140 Population and Development, Facultad Latinoamericana de Ciencias Sociales Mexico, Mexico City, Mexico; 141 Department of Medical Laboratory Sciences, Bahir Dar University, Bahir Dar, Ethiopia; 142 Department of Nursing, Woldia University, Woldia, Ethiopia; 143 School of Nursing, Jimma University, Jimma, Ethiopia; 144 School of Pharmacy, Aksum University, Aksum, Ethiopia; 145 Addis Ababa University, Addis Ababa, Ethiopia; 146 Immunology Research Center, Tabriz University of Medical Sciences, Iran; 147 Department of Global Health and Infection, Brighton and Sussex Medical School, Brighton, UK; 148 School of Public Health, Addis Ababa University, Addis Ababa, Ethiopia; 149 Division of Cardiology, Atlanta Veterans Affairs Medical Center, Decatur, GA, USA; 150 Department of Epidemiology, Shiraz University of Medical Sciences, Shiraz, Iran; 151 Faculty of Pharmacy, University of Porto, Porto, Portugal; 152 Tehran University of Medical Sciences, Tehran, Iran; 153 School of Health and Biomedical Sciences, Royal Melbourne Institute of Technology University, Bundoora, VIC, Australia; 154 Sydney School of Public Health, University of Sydney, Sydney, NSW, Australia; 155 Faculty of Medicine, University of Belgrade, Belgrade, Serbia; 156 Public Health Department, Hawassa University, Hawassa, Ethiopia; 157 Curtin University, Perth, WA, Australia; 158 Department of Global Health and Social Medicine, Harvard University, Boston, MA, USA; 159 Department of Social Services, Tufts Medical Center, Boston, MA, USA; 160 Department of Pharmacology and Toxicology, Maragheh University of Medical Sciences, Maragheh, Iran; 161 Department of Pharmacology and Toxicology, Tabriz University of Medical Sciences, Tabriz, Iran; 162 National Institute for Health Researches, Tehran University of Medical Sciences, Tehran, Iran; 163 Department of Clinical Pathology, Mansoura University, Mansoura, Egypt; 164 Department of Statistics, Debre Markos University, Debre Markos, Ethiopia; 165 Department of Public Health Sciences, Karolinska Institutet, Stockholm, Sweden; 166 World Health Programme, Université du Québec en Abitibi-Témiscamingue, Rouyn-Noranda, QC, Canada; 167 Department of Pathology, Stavanger University Hospital, Stavanger, Norway; 168 Norwegian Institute of Public Health, Oslo, Norway; 169 Ophthalmic Epidemiology Research Center, Shahroud University of Medical Sciences, Shahroud, Iran; 170 Department of Midwifery, Wolkite University, Wolkite, Ethiopia; 171 Multiple Sclerosis Research Center, Tehran University of Medical Sciences, Tehran, Iran; 172 Biology Department, Salahaddin University-Erbil, Erbil, Iraq; 173 Biology and Biotechnolaniogy"L Spallanzani", University of Pavia, Pavia, Italy; 174 Department of Psychology, Federal University of Sergipe, Sao Cristovao, Brazil; 175 Non-communicable Diseases Research Center, Tehran University of Medical Sciences, Tehran, Iran; 176 Pharmaceutical Nanotechnology, Tehran University of Medical Sciences, Tehran, Iran; 177 Department of Psychiatry, Addis Ababa University, Addis Ababa, Ethiopia; 178 Nursing Department, Hawassa University, Hawassa, Ethiopia; 179 Department of Neurobiology, Karolinska Institutet, Stockholm, Sweden; 180 Division of Neurology, University of Ottawa, Ottawa, ON, Canada; 181 REQUIMTE/LAQV, University of Porto, Porto, Portugal; 182 Research Centre on Public Health (CESP), University of Milan Bicocca, Monza, Italy; 183 Department of Health Education & Behavioral Sciences, Jimma University, Jimma, Ethiopia; 184 Psychiatry Department, Kaiser Permanente, Fontana, CA, USA; 185 School of Health Sciences, A T Still University, Mesa, AZ, USA; 186 Department of Population Medicine and Health Services Research, Bielefeld University, Bielefeld, Germany; 187 Department of Child Dental Health, Obafemi Awolowo University, Ile-Ife, Nigeria; 188 Abadan School of Medical Sciences, Abadan University of Medical Sciences, Abadan, Iran; 189 Department of Family Medicine and Primary Care, University of the Witwatersrand, Johannesburg, South Africa; 190 College of Public Health, Medical and Veterinary Science, James Cook University, Douglas, QLD, Australia; 191 Royal Life Saving Society, Sydney, NSW, Australia; 192 Department of Dermatology, Kobe University, Kobe, Japan; 193 Gene Expression & Regulation Program, The Wistar Institute, Philadelphia, PA, USA; 194 Public Health Department, Madda Walabu University, Bale-Robe, Ethiopia; 195 School of Pharmacy, Mekelle University, Mekelle, Ethiopia; 196 Department of Nursing and Midwifery, Addis Ababa University, Addis Ababa, Ethiopia; 197 Department of Nursing, Aksum University, Aksum, Ethiopia; 198 Department of Nursing, Mekelle University, Mekelle, Ethiopia; 199 School of Public Health, Haramaya University, Harar, Ethiopia; 200 Bahir Dar University, Bahir Dar, Ethiopia; 201 Haramaya University, Dire Dawa, Ethiopia; 202 Department of Pharmacy, Wollo University, Dessie, Ethiopia; 203 Department of Medical Surgery, Tabriz University of Medical Sciences, Tabriz, Iran; 204 Occupational Health Department, Arak University of Medical Sciences, Arak, Iran; 205 Department of Health Services Management, Iran University of Medical Sciences, Tehran, Iran; 206 Science and Research Branch, Islamic Azad University, Tehran, Iran; 207 Young Researchers and Elite Club, Rasht Branch, Islamic Azad University, Rasht, Iran; 208 Adelaide Medical School, University of Adelaide, Adelaide, SA, Australia; 209 Center for Clinical and Epidemiological Research, University of São Paulo, Sao Paulo, Brazil; 210 Department of Dermatology, Boston University, Boston, MA, USA; 211 Institute of Public Health, United Arab Emirates University, Al Ain, United Arab Emirates; 212 Technical College of Health, Sulaimani Polytechnic University, Sulaimani, Iraq; 213 Instituto de Patologia Tropical e Saúde Pública, Federal University of Goias, Goiânia, Brazil; 214 School of Public Health and Preventive Medicine, Monash University, Melbourne, VIC, Australia; 215 Department of Epidemiology and Biostatistics, Zhengzhou University, Zhengzhou, China; 216 Non-Communicable Diseases (NCD), World Health Organization (WHO), New Delhi, India; 217 Department of Public Health, Erasmus University Medical Center, Rotterdam, Netherlands; 218 Department of Radiology and Radiological Sciences, Johns Hopkins University, Baltimore, MD, USA; 219 School of Medicine, Tehran University of Medical Sciences, Tehran, Iran; 220 Institute for Global Health, University College London, London, UK; 221 Global and Community Mental Health Research Group, University of Macau, Macao, China; 222 Department of Family and Community Medicine, Arabian Gulf University, Manama, Bahrain; 223 School of Health and Environmental Studies, Hamdan Bin Mohammed Smart University, Dubai, United Arab Emirates; 224 Biomedical Research Networking Center for Mental Health Network (CiberSAM), Madrid, Spain; 225 Research and Development Unit, San Juan de Dios Sanitary Park, Sant Boi de Llobregat, Spain; 226 Institute for Social Science Research, The University of Queensland, Indooroopilly, QLD, Australia; 227 Department of Microbiology, Maragheh University of Medical Sciences, Maragheh, Iran; 228 Department of Microbiology, Tehran University of Medical Sciences, Tehran, Iran; 229 Gastrointestinal and Liver Disease Research Center, Guilan University of Medical Sciences, Rasht, Iran; 230 School of Nursing and Midwifery, Tabriz University of Medical Sciences, Tabriz, Iran; 231 Independent Consultant, Tabriz, Iran; 232 Department of Public Health, Mizan-Tepi University, Tepi, Ethiopia; 233 Unit of Epidemiology and Social Medicine, University Hospital Antwerp, Wilrijk, Belgium; 234 Department of Clinical Sciences, Karolinska University Hospital, Stockholm, Sweden; 235 Institute of Pharmaceutical Sciences, University of Veterinary and Animal Sciences, Lahore, Pakistan; 236 Department of Pharmacy Administration and Clinical Pharmacy, Xian Jiaotong University, Xian, China; 237 Medical Biology Research Center, Kermanshah University of Medical Sciences, Kermanshah, Iran; 238 Research Coordination, AC Environments Foundation, Cuernavaca, Mexico; 239 CISS, National Institute of Public Health, Cuernavaca, Mexico; 240 Department of Urban Planning and Design, University of Hong Kong, Hong Kong, China; 241 Center of Excellence in Behavioral Medicine, Nguyen Tat Thanh University, Ho Chi Minh City, Vietnam; 242 Department of Pediatrics, Dell Medical School, University of Texas Austin, Austin, TX, USA; 243 Kasturba Medical College, Manipal Academy of Higher Education, Manipal, India; 244 Department of Pharmacology and Therapeutics, Dhaka Medical College, Dhaka, Bangladesh; 245 Department of Pharmacology, Bangladesh Industrial Gases Limited, Tangail, Bangladesh; 246 Department of Computer Engineering, Islamic Azad University, Tehran, Iran; 247 Computer Science Department, University of Human Development, Sulaymaniyah, Iraq; 248 Department of Legal Medicine and Bioethics, Carol Davila University of Medicine and Pharmacy, Bucharest, Romania; 249 Clinical Legal Medicine Department, National Institute of Legal Medicine Mina Minovici, Bucharest, Romania; 250 Department of Epidemiology and Health Statistics, Central South University, Changsha, China; 251 Department of Health Promotion and Education, University of Ibadan, Ibadan, Nigeria; 252 Department of Community Medicine, University of Ibadan, Ibadan, Nigeria; 253 Department of Epidemiology, University of Kragujevac, Kragujevac, Serbia; 254 Department of Family Medicine, Bangalore Baptist Hospital, Bangalore, India; 255 Center for Health Resource and Services Research and Development, National Institute of Health Research & Development, Jakarta, Indonesia; 256 Research Institute for Endocrine Sciences, Shahid Beheshti University of Medical Sciences, Tehran, Iran; 257 Institute for Physical Activity and Nutrition, Deakin University, Burwood, VIC, Australia; 258 Sydney Medical School, University of Sydney, Sydney, NSW, Australia; 259 School of Psychology and Public Health, La Trobe University, Bundoora, Melbourne, VIC, Australia; 260 School of Public Health and Community Medicine, University of New South Wales, Sydney, Australia; 261 Department of Global and Community Health, George Mason University, Fairfax, VA, USA; 262 Faculty of Medicine, Babol University of Medical Sciences, Babol, Iran; 263 School of Management and Medical Education, Shahid Beheshti University of Medical Sciences, Tehran, Iran; 264 Safety Promotion and Injury Prevention Research Center, Shahid Beheshti University of Medical Sciences, Tehran, Iran; 265 Department for Health Care and Public Health, I M Sechenov First Moscow State Medical University, Moscow, Russia; 266 Social Development & Health Promotion Research Center, Kermanshah University of Medical Sciences, Kermanshah, Iran; 267 Department of Surgery, Virginia Commonwealth University, Richmond, VA, USA; 268 Institute of Medicine, University of Colombo, Colombo, Sri Lanka; 269 Faculty of Graduate Studies, University of Colombo, Colombo, Sri Lanka; 270 Department of Community Medicine, Banaras Hindu University, Varanasi, India; 271 Department of Ophthalmology, Heidelberg University, Mannheim, Germany; 272 Beijing Ophthalmology & Visual Science Key Laboratory, Beijing Tongren Hospital, Beijing, China; 273 Department of Community Medicine, Kasturba Medical College, Manipal Academy of Higher Education, Mangalore, India; 274 Department of Family Medicine and Public Health, University of Opole, Opole, Poland; 275 School of Health Sciences, Savitribai Phule Pune University, Pune, India; 276 Institute of Family Medicine and Public Health, University of Tartu, Tartu, Estonia; 277 Minimally Invasive Surgery Research Center, Iran University of Medical Sciences, Tehran, Iran; 278 Personal Social Services Research Unit, London School of Economics and Political Science, London, UK; 279 Department of Medical Informatics, Tabriz University of Medical Sciences, Tabriz, Iran; 280 Social Determinants of Health Research Center, Research Institute for Prevention of Non-Communicable Diseases, Qazvin University of Medical Sciences, Qazvin, Iran; 281 Health Services Management Department, Qazvin University of Medical Sciences, Qazvin, Iran; 282 School of Public Health, Department of Health Informatics and Health Innovation, A C S Medical College and Hospital, Mekelle, Ethiopia; 283 Department of Forensic Medicine and Toxicology, All India Institute of Medical Sciences, Jodhpur, India; 284 Department of Epidemiology, Hamadan University of Medical Sciences, Hamadan, Iran; 285 Hematology-Oncology and Stem Cell Transplantation Research Center, Tehran University of Medical Sciences, Tehran, Iran; 286 Pars Advanced and Minimally Invasive Medical Manners Research Center, Iran University of Medical Sciences, Tehran, Iran; 287 Department of Applied Physics, The John Paul II Catholic University of Lublin, Lublin Voivodeship, Poland; 288 Department of Biology and Chemistry, Drohobych Ivan Franko State Pedagogical University, Drohobych, Ukraine; 289 Department of Pharmacy, Jimma University, Jimma, Ethiopia; 290 Open, Distance and eLearning Campus, University of Nairobi, Nairobi, Kenya; 291 Department of Dermatology, Wolaita Sodo University, Wolaita Sodo, Ethiopia; 292 Department of Midwifery, University of Gondar, Gondar, Ethiopia; 293 Department of Public Health, Jordan University of Science and Technology, Irbid, Jordan; 294 Social Determinants of Health Research Center, Ahvaz Jundishapur University of Medical Sciences, Ahvaz, Iran; 295 School of Food and Agricultural Sciences, University of Management and Technology, Lahore, Pakistan; 296 Department of Global Health, University of Washington, Seattle, WA, USA; 297 Department of Physiology, Baku State University, Baku, Azerbaijan; 298 Health Care Management, Zahedan University of Medical Sciences, zahedan, Iran; 299 Epidemiology and Biostatistics Department, Health Services Academy, Islamabad, Pakistan; 300 Faculty of Public Health and Tropical Medicine, Jazan University, Jazan, Saudi Arabia; 301 Department of Health Research, Indian Council of Medical Research, New Delhi, India; 302 Centre for Ethics, Jawahar Lal Nehru University, New Delhi, India; 303 Department of Psychiatry, Kermanshah University of Medical Sciences, Kermanshah, Iran; 304 Department of Epidemiology, Kermanshah University of Medical Sciences, Kermanshah, Iran; 305 Nuffield Department of Surgical Sciences, Oxford University Global Surgery Group, University of Oxford, Oxford, UK; 306 Research and Data Solutions, Synotech Consultant, Nairobi, Kenya; 307 Department of Preventive Medicine, Korea University, Seoul, South Korea; 308 Department of Health Sciences, Northeastern University, Boston, MA, USA; 309 School of Medicine, Xiamen University Malaysia, Sepang, Malaysia; 310 School of Health Sciences, Kristiania University College, Oslo, Norway; 311 Department of Nursing and Health Promotion, Oslo Metropolitan University, Oslo, Norway; 312 Neurophysiology Research Center, Hamadan University of Medical Sciences, Hamadan, Iran; 313 Brain Engineering Research Center, Institute for Research in Fundamental Sciences, Tehran, Iran; 314 Department of Public Health Dentistry, Deemed University, karad, India; 315 CIBERSAM, San Juan de Dios Sanitary Park, Sant Boi de Llobregat, Spain; 316 Catalan Institution for Research and Advanced Studies (ICREA), Barcelona, Spain; 317 Department of Zoology, University of Oxford, Oxford, UK; 318 Harvard Medical School, Harvard University, Boston, MA, USA; 319 Department of Anthropology, Panjab University, Chandigarh, India; 320 Department of Public Health, Yuksek Ihtisas University, Ankara, Turkey; 321 Department of Public Health, Hacettepe University, Ankara, Turkey; 322 Department of Family and Community Health, University of Health and Allied Sciences, Ho, Ghana; 323 Department of Psychology and Health Promotion, University of KwaZulu-Natal, Durban, South Africa; 324 Department of Medicine, Brigham and Women’s Hospital, Harvard University, Boston, MA, USA; 325 Public Health Foundation of India, Gurugram, India; 326 Department of Psychiatry, University of Nairobi, Nairobi, Kenya; 327 Institute of Occupational and Environmental Medicine, University of Birmingham, Birmingham, UK; 328 Mechanical and Industrial Engineering, Indian Institute of Technology, Roorkee, Roorkee, India; 329 Department of Medicine, McMaster University, Hamilton, ON, Canada; 330 Health and Nutrition Section, United Nations Childrens’ Fund (UNICEF), Accra, Ghana; 331 Department of Clinical Medicine and Community Health, University of Milan, Milano, Italy; 332 Department of Community and Family Medicine, University of Baghdad, Baghdad, Iraq; 333 School of Medicine, Deakin University, Geelong, VIC, Australia; 334 Health Promotion and Chronic Disease Prevention Branch, Public Health Agency of Canada, Ottawa, ON, Canada; 335 HelpMeSee, New York, NY, USA; 336 International Relations, Mexican Institute of Ophthalmology, Queretaro, Mexico; 337 Disease Control Department, Ghana Health Service, Accra, Ghana; 338 Department of Otorhinolaryngology (ENT), Father Muller Medical College, Mangalore, India; 339 Department of Public Health, Maragheh University of Medical Sciences, Maragheh, Iran; 340 Institute of Clinical Physiology, Italian National Research Council, Pisa, Italy; 341 College of Optometry, Nova Southeastern University, Fort Lauderdale, FL, USA; 342 School of Pharmacy, Monash University, Bandar Sunway, Malaysia; 343 School of Pharmacy, Taylor’s University Lakeside Campus, Subang Jaya, Malaysia; 344 School of Public Health, Wolaita Sodo University, Wolaita Sodo, Ethiopia; 345 Department of Health Sciences, University of Florence, Florence, Italy; 346 School of Public Health, University of Haifa, Haifa, Israel; 347 Department of Systems, Populations and Leadership, University of Michigan, Ann Arbor, MI, USA; 348 School of Population and Global Health, University of Melbourne, Melbourne, VIC, Australia; 349 Department of Health Metrics Sciences, School of Medicine, University of Washington, Seattle, WA, USA; 350 Department of Medicine, University of São Paulo, Sao Paulo, Brazil; 351 Department of General Surgery, Aintree University Hospital National Health Service (NHS) Foundation Trust, Liverpool, UK; 352 Department of Surgery, University of Liverpool, Liverpool, UK; 353 Health Data Research UK, Swansea University, Swansea, UK; 354 College of Medicine, Pathology Department, Imam Abdulrahman Bin Faisal University, Dammam, Saudi Arabia; 355 Ophthalmology Department, Aswan Faculty of Medicine, Aswan, Egypt; 356 Institute of Medicine, Tribhuvan University, Kathmandu, Nepal; 357 Department of Public Health, Trnava University, Trnava, Slovakia; 358 Department of Primary Care and Public Health, Imperial College London, London, UK; 359 Public Health Research Department, National Health Institute Colombia, Bogota, Colombia; 360 Faculty of Medicine, El Bosque University, Bogota, Colombia; 361 Health Education and Research Department, SDM College of Medical Sciences & Hospital, Dharwad, India; 362 Rajiv Gandhi University of Health Sciences, Bangalore, India; 363 Digestive Diseases Research Institute, Tehran University of Medical Sciences, Tehran, Iran; 364 Non-communicable Diseases Research Center, Shiraz University of Medical Sciences, Shiraz, Iran; 365 Department of Maternal and Child Nursing and Public Health, Federal University of Minas Gerais, Belo Horizonte, Brazil; 366 Department of Ophthalmology, Iran University of Medical Sciences, Tehran, Iran; 367 Ophthalmology Department, University of Manitoba, Winnipeg, MB, Canada; 368 Department of Surgery, University of Virginia, Charlottesville, VA, USA; 369 Surgery Department, Emergency University Hospital Bucharest, Bucharest, Romania; 370 Department of Psychiatry, National Institute of Mental Health and Neurosciences, Bengaluru, India; 371 Substance Abuse Prevention Research Center, Kermanshah University of Medical Sciences, Kermanshah, Iran; 372 Department of Epidemiology and Biostatistics, Tehran University of Medical Sciences, Tehran, Iran; 373 Institute for Social Science Research, The University of Queensland, Brisbane, QLD, Australia; 374 Institute of Bone and Joint Research, University of Sydney, St Leonards, NSW, Australia; 375 Department of Health Sciences, University of York, York, UK; 376 Department of Midwifery-Reproductive Health, Hamadan University of Medical Sciences, Hamadan, Iran; 377 Research Department, The George Institute for Global Health, New Delhi, India; 378 School of Medicine, University of New South Wales, Sydney, NSW, Australia; 379 Department of Public Health, Mekelle University, Mekelle, Ethiopia; 380 Department of Nursing, Arba Minch University, Arba Minch, Ethiopia; 381 Division of Epidemiology and Prevention, Institute of Human Virology, University of Maryland, Baltimore, MD, USA; 382 Peru Country Office, United Nations Population Fund (UNFPA), Lima, Peru; 383 Forensic Medicine Division, Imam Abdulrahman Bin Faisal University, Dammam, Saudi Arabia; 384 College of Health Science, Department of Midwifery, Adigrat University, Adigrat, Ethiopia; 385 Department of Epidemiology and Biostatistics, Haramaya University, Harar, Ethiopia; 386 Breast Surgery Unit, Helsinki University Hospital, Helsinki, Finland; 387 University of Helsinki, Helsinki, Finland; 388 Neurocenter, Helsinki University Hospital, Helsinki, Finland; 389 School of Health Sciences, University of Melbourne, Parkville, VIC, Australia; 390 Clinical Microbiology and Parasitology Unit, ZoraProfozic Polyclinic, Zagreb, Croatia; 391 University Centre Varazdin, University North, Varazdin, Croatia; 392 Center for Innovation in Medical Education, Pomeranian Medical University, Szczecin, Poland; 393 Department of Propedeutics of Internal Diseases & Arterial Hypertension, Pomeranian Medical University, Szczecin, Poland; 394 Pacific Institute for Research & Evaluation, Calverton, MD, USA; 395 Achutha Menon Centre for Health Science Studies, Sree Chitra Tirunal Institute for Medical Sciences and Technology, Trivandrum, India; 396 Global Institute of Public Health (GIPH), Ananthapuri Hospitals and Research Centre, Trivandrum, India; 397 Department of Statistics and Econometrics, Bucharest University of Economic Studies, Bucharest, Romania; 398 President’s Office, National Institute of Statistics Romania, Bucharest, Romania; 399 Faculty of Internal Medicine, Kyrgyz State Medical Academy, Bishkek, Kyrgyzstan; 400 Department of Atherosclerosis and Coronary Heart Disease, National Center of Cardiology and Internal Disease, Bishkek, Kyrgyzstan; 401 Heidelberg Institute of Global Health (HIGH), Faculty of Medicine and University Hospital, Heidelberg University, Heidelberg, Germany; 402 Institute of Addiction Research (ISFF), Frankfurt University of Applied Sciences, Frankfurt, Germany; 403 Biotechnology Research Center, Tabriz University of Medical Sciences, Tabriz, Iran; 404 Molecular Medicine Research Center, Tabriz University of Medical Sciences, Tabriz, Iran; 405 Health Equity Research Center, Tehran University of Medical Sciences, Tehran, Iran; 406 Internal Medicine Department, King Saud University, Riyadh, Saudi Arabia; 407 Research Center, Salahaddin University, Erbil, Iraq; 408 Ishik University, Erbil, Iraq; 409 Department of Information Technology, University of Human Development, Sulaymaniyah, Iraq; 410 Department of Biostatistics, Hamadan University of Medical Sciences, Hamadan, Iran; 411 Department of Epidemiology and Biostatistics, Shahrekord University of Medical Sciences, Shahrekord, Iran; 412 Department of Clinical Biochemistry, Faculty of Medicine, Gonabad University of Medical Sciences, Gonabad, Iran; 413 Department of Nursing, Shahroud University of Medical Sciences, Shahroud, Iran; 414 Health Systems and Policy Research Unit, Ahmadu Bello University, Zaria, Nigeria; 415 Department of Public Health, Samara University, Samara, Ethiopia; 416 Iran National Institute of Health Research, Tehran University of Medical Sciences, Tehran, Iran; 417 Faculty of Life Sciences and Medicine, King’s College London, London, UK; 418 Clinical Epidemiology and Public Health Research Unit, Burlo Garofolo Institute for Maternal and Child Health, Trieste, Italy; 419 Department of Public Health Medicine, University of KwaZulu-Natal, Durban, South Africa; 420 Health Sciences Research Center, Mazandaran University of Medical Sciences, Sari, Iran; 421 Research Center for Environmental Determinants of Health, Kermanshah University of Medical Sciences, Kermanshah, Iran; 422 Social Determinants of Health Research Center, Kurdistan University of Medical Sciences, Sanandaj, Iran; 423 Department of Epidemiology and Biostatistics, Kurdistan University of Medical Sciences, Sanandaj, Iran; 424 Preventive Medicine and Public Health Research Center, Iran University of Medical Sciences, Tehran, Iran; 425 International Laboratory for Air Quality and Health, Queensland University of Technology, Brisbane, QLD, Australia; 426 Gorgas Memorial Institute for Health Studies, Panama City, Panama; 427 Department of Social Medicine, National Center for Child Health and Development, Setagaya, Japan; 428 Department of Epidemiology and Biostatistics, University of Gondar, Gondar, Ethiopia; 429 Department of Pediatrics, University of British Columbia, Vancouver, BC, Canada; 430 School of Medical Sciences, Science University of Malaysia, Kubang Kerian, Malaysia; 431 Department of Pediatric Medicine, Nishtar Medical University, Multan, Pakistan; 432 Department of Pediatrics & Pediatric Pulmonology, Institute of Mother & Child Care, Multan, Pakistan; 433 Department of Obstetrics and Gynecology, Ain Shams University, Cairo, Egypt; 434 Knowledge Translation and Utilization, Egyptian Center for Evidence Based Medicine, Egypt; 435 Research and Analytics, Initiative for Financing Health and Human Development, Chennai, India; 436 Research and Analytics, Bioinsilico Technologies, Chennai, India; 437 Department of Epidemiology, University of Alabama at Birmingham, Birmingham, AL, USA; 438 Laboratory of Public Health Indicators Analysis and Health Digitalization, Moscow Institute of Physics and Technology, Dolgoprudny, Russia; 439 Experimental Surgery and Oncology Laboratory, Kursk State Medical University of the Ministry of Health of the Russian Federation, Kursk, Russia; 440 Department of Epidemiology & Biostatistics, Kermanshah University of Medical Sciences, Kermanshah, Iran; 441 Suraj Eye Institute, Nagpur, India; 442 Hospital of the Federal University of Minas Gerais, Federal University of Minas Gerais, Belo Horizonte, Brazil; 443 Mental Health Research Center, Iran University of Medical Sciences, Tehran, Iran; 444 Department of Forensic Medicine and Toxicology, Manipal Academy of Higher Education, Manipal, India; 445 Cochrane Unit, South African Medical Research Council, Cape Town, South Africa; 446 Department of General Surgery, Carol Davila University of Medicine and Pharmacy, Bucharest, Romania; 447 Department of General Surgery, Emergency Hospital of Bucharest, Bucharest, Romania; 448 Department of Biological Sciences, University of Embu, Embu, Kenya; 449 Institute for Global Health Innovations, Duy Tan University, Hanoi, Vietnam; 450 Department of Pharmacology, Shahid Beheshti University of Medical Sciences, Tehran, Iran; 451 Heidelberg University Hospital, Heidelberg, Germany; 452 Public Health Department, Universitas Negeri Semarang, Kota Semarang, Indonesia; 453 Graduate Institute of Biomedical Informatics, Taipei Medical University, Taipei City, Taiwan; 454 School of Public Health and Family Medicine, University of Cape Town, Cape Town, South Africa; 455 Faculty of Medicine & Health Sciences, Stellenbosch University, Cape Town, South Africa; 456 Reproductive Health Sciences, Department Obstetrics and Gynecology, University of Ibadan, Ibadan, Nigeria; 457 Department of Preventive Medicine, Kyung Hee University, Dongdaemun-gu, South Korea; 458 Disease Surveillance and Epidemic Response, Ministry of Health, Nairobi, Kenya; 459 Department of Psychiatry and Behavioural Neurosciences, McMaster University, Hamilton, ON, Canada; 460 Department of Psychiatry, University of Lagos, Lagos, Nigeria; 461 Department of Pathology and Molecular Medicine, McMaster University, Hamilton, ON, Canada; 462 Diplomacy and Public Relations Department, University of Human Development, Sulaimaniyah, Iraq; 463 Department of Pharmacology and Therapeutics, University of Nigeria Nsukka, Enugu, Nigeria; 464 Department of Psychology, University of Ghana, Accra, Ghana; 465 Discipline of Psychology, University of KwaZulu-Natal, Durban, South Africa; 466 Applied Research Division, Public Health Agency of Canada, Ottawa, ON, Canada; 467 School of Psychology, University of Ottawa, Ottawa, ON, Canada; 468 Department of Global Health Nursing, St Luke’s International University, Chuo-ku, Japan; 469 Academic department, Unium Ltd, Moscow, Russia; 470 Department of Project Management, National Research University Higher School of Economics, Moscow, Russia; 471 Department of Respiratory Medicine, Jagadguru Sri Shivarathreeswara Academy of Health Education and Research, Mysore, India; 472 Department of Forensic Medicine, Manipal Academy of Higher Education, Manipal, India; 473 Department of Medicine, Ottawa Hospital Research Institute, Ottawa ON, Canada; 474 Parasitology and Mycology, Shiraz University of Medical Sciences, Shiraz, Iran; 475 Augenpraxis Jonas, Heidelberg University, Heidelberg, Germany; 476 Department of Medical Humanities and Social Medicine, Kosin University, Busan, South Korea; 477 Research and Evaluation, Population Council, New Delhi, India; 478 Indian Institute of Health Management Research University Delhi, Jaipur, India; 479 Department of Pediatircs, RD Gardi Medical College, Ujjain, India; 480 Regional Medical Research Centre, Indian Council of Medical Research, Bhubaneswar, India; 481 Department of Paediatrics, University of Melbourne, Melbourne, VIC, Australia; 482 Population Health Theme, Murdoch Childrens Research Institute, Melbourne, VIC, Australia; 483 Department of Midwifery, Wolaita Sodo University, Wolaita Sodo, Ethiopia; 484 School of Public Health and Community Medicine, Faculty of Medicine, University of New South Wales, Sydney, NSW, Australia; 485 Center for Research and Innovation, Ateneo De Manila University, Pasig City, Philippines; 486 Department of Orthopedics, Yenepoya Medical College, Mangalore, India; 487 Shanghai Mental Health Center, Shanghai Jiao Tong University, Shanghai, China; 488 Department of Psychiatry, Department of Epidemiology, Columbia University, New York City, NY, USA; 489 Department of Chemistry, Faculty of Pharmacy, University of Porto, Porto, Portugal; 490 Department of Epidemiology and Evidence-Based Medicine, Sechenon University, Moscow, Russia; 491 Department of Nephrology, Sanjay Gandhi Postgraduate Institute of Medical Sciences, Lucknow, India; 492 Health Sciences Department, Muhammadiyah University of Surakarta, Sukoharjo, Indonesia; 493 Department of Population Studies, International Institute for Population Sciences, Mumbai, India; 494 Biomedical Engineering Department, Amirkabir University of Technology, Tehran, Iran; 495 Department of Chemistry, Sharif University of Technology, Tehran, Iran; 496 College of Medicine, University of Central Florida, Orlando, FL, USA; 497 College of Graduate Health Sciences, A T Still University, Mesa, AZ, USA; 498 Department of Epidemiology & Biostatistics, Contech School of Public Health, Lahore, Pakistan; 499 Department of Medicine, University of Alberta, Edmonton, AB, Canada; 500 Department of Immunology, Mazandaran University of Medical Sciences, Sari, Iran; 501 Molecular and Cell Biology Research Center, Mazandaran University of Medical Sciences, Sari, Iran; 502 Thalassemia and Hemoglobinopathy Research Center, Ahvaz Jundishapur University of Medical Sciences, Ahvaz, Iran; 503 Metabolomics and Genomics Research Center, Tehran University of Medical Sciences, Tehran, Iran; 504 Faculty of Medicine, Mazandaran University of Medical Sciences, Sari, Iran; 505 Sina Trauma and Surgery Research Center, Tehran University of Medical Sciences, Tehran, Iran; 506 School of Nursing and Healthcare Professions, Federation University Australia, Berwick, VIC, Australia; 507 School of Nursing and Midwifery, La Trobe University, Melbourne, VIC, Australia; 508 Faculty of Medicine, Birjand University of Medical Sciences, Birjand, Iran; 509 European Office for the Prevention and Control of Noncommunicable Diseases, World Health Organization (WHO), Moscow, Russia; 510 Department of Surgery, University of Michigan, Ann Arbor, MI, USA; 511 Department of Oral Pathology, Srinivas Institute of Dental Sciences, Mangalore, India; 512 School of Behavioral Sciences and Mental Health, Tehran Institute of Psychiatry, Tehran, Iran; 513 Forensic Medicine, Manipal Academy of Higher Education, Mangalore, India; 514 Kasturba Medical College, Manipal Academy of Higher Education, Mangalore, India; 515 Academic Public Health Department, Public Health England, London, UK; 516 School of Health, Medical and Applied Sciences, CQ University, Sydney, NSW, Australia; 517 Department of Computer Science, Metropolitan College, Boston University, Boston, USA; 518 Neurology Department, Sree Chitra Tirunal Institute for Medical Sciences and Technology, Thiruvananthapuram, India; 519 School of Social Sciences and Psychology, Western Sydney University, Penrith, NSW, Australia; 520 Translational Health Research Institute, Western Sydney University, Penrith, NSW, Australia; 521 Brien Holden Vision Institute, Sydney, NSW, Australia; 522 Organization for the Prevention of Blindness, Paris, France; 523 EPIUnit - Public Health Institute University Porto (ISPUP), University of Porto, Porto, Portugal; 524 Surgery Department, University of Minnesota, Minneapolis, MN, USA; 525 Surgery Department, University Teaching Hospital of Kigali, Kigali, Rwanda; 526 Research Directorate, Nihon Gakko University, Fernando de la Mora, Paraguay; 527 Research Direction, Universidad Nacional de Caaguazú, Coronel Oviedo, Paraguay; 528 Golestan Research Center of Gastroenterology and Hepatology, Golestan University of Medical Sciences, Gorgan, Iran; 529 Faculty of Medicine, Ain Shams University, Cairo, Egypt; 530 National Institute for Research in Environmental Health, Indian Council of Medical Research, Bhopal, India; 531 Department of Epidemiology, Shahid Beheshti University of Medical Sciences, Tehran, Iran; 532 College of Medicine, University of Sharjah, Sharjah, United Arab Emirates; 533 Emergency Department, Shahid Beheshti University of Medical Sciences, Tehran, Iran; 534 Faculty of Public Health, Kermanshah University of Medical Sciences, Kermanshah, Iran; 535 Department of Health in Disasters and Emergencies, Shahid Beheshti University of Medical Sciences, Tehran, Iran; 536 Department of Neuroscience, Iran University of Medical Sciences, Tehran, Iran; 537 Nanobiotechnology Center, Soran University, Soran, Iraq; 538 Taleghani Hospital, Kermanshah University of Medical Sciences, Kermanshah, Iran; 539 Radiology and Nuclear Medicine Department, Kermanshah University of Medical Sciences, Kermanshah, Iran; 540 Research Deputy, Taleghani Hospital, Kermanshah, Iran; 541 Public Health and Community Medicine Department, Cairo University, Giza, Egypt; 542 Department of Urology, Cairo University, Cairo, Egypt; 543 Public Health and Policy, London School of Hygiene & Tropical Medicine, London, UK; 544 Global Health Institute, American University of Beirut, Beirut, Lebanon; 545 Health and Disability Intelligence Group, Ministry of Health, Wellington, New Zealand; 546 Department of Entomology, Ain Shams University, Cairo, Egypt; 547 Department of Surgery, Marshall University, Huntington, WV, USA; 548 Department of Nutrition and Preventive Medicine, Case Western Reserve University, Cleveland, OH, USA; 549 Rheumatology Department, University Hospitals Bristol NHS Foundation Trust, Bristol, UK; 550 Institute of Bone and Joint Research, University of Sydney, Syndey, NSW, Australia; 551 Institute of Social Medicine, University of Belgrade, Belgrade, Serbia; 552 Centre-School of Public Health and Health Management, University of Belgrade, Belgrade, Serbia; 553 Health Economics, Bangladesh Institute of Development Studies (BIDS), Dhaka, Bangladesh; 554 Colorectal Research Center, Iran University of Medical Sciences, Tehran, Iran; 555 Surgery Department, Hamad Medical Corporation, Doha, Qatar; 556 Faculty of Health & Social Sciences, Bournemouth University, Bournemouth, UK; 557 Department of Public Health Sciences, University of North Carolina at Charlotte, Charlotte, NC, USA; 558 Department of Paediatrics, University of Melbourne, Parkville, VIC, Australia; 559 Centre for Adolescent Health, Murdoch Childrens Research Institute, Parkville, VIC, Australia; 560 School of Public Health, Imperial College London, London, UK; 561 Faculty member of Education Development Center, Ahvaz Jundishapur University of Medical Sciences, Ahvaz, Iran; 562 Department of Psychology, University of Alabama at Birmingham, Birmingham, AL, USA; 563 Department of Psychiatry, Stellenbosch University, Cape Town, South Africa; 564 Emergency Department, Manian Medical Centre, Erode, India; 565 Microbiology Service, National Institutes of Health, Bethesda, MD, USA; 566 Center for Biomedical Information Technology, Shenzhen Institutes of Advanced Technology, Chinese Academy of Sciences, Shenzhen, China; 567 Department of Health Promotion and Education, Alborz University of Medical Sciences, Karaj, Iran; 568 Health Policy Research Center, Shiraz University of Medical Sciences, Shiraz, Iran; 569 Independent Consultant, Karachi, Pakistan; 570 School of Medicine, Alborz University of Medical Sciences, Karaj, Iran; 571 Chronic Diseases (Home Care) Research Center, Hamadan University of Medical Sciences, Hamadan, Iran; 572 HIV/STI Surveillance Research Center, and WHO Collaborating Center for HIV Surveillance, Institute for Futures Studies in Health, Kerman University of Medical Sciences, Kerman, Iran; 573 Centre for Medical Informatics, University of Edinburgh, Edinburgh, UK; 574 Division of General Internal Medicine, Harvard University, Boston, MA, USA; 575 National Institute of Infectious Diseases, Tokyo, Japan; 576 College of Medicine, Yonsei University, Seodaemun-gu, South Korea; 577 Division of Cardiology, Emory University, Atlanta, GA, USA; 578 Finnish Institute of Occupational Health, Helsinki, Finland; 579 Department of Health Education & Promotion, Kermanshah University of Medical Sciences, Kermanshah, Iran; 580 School of Health, University of Technology Sydney, Sydney, NSW, Australia; 581 Department of Psychology, Reykjavik University, Reykjavik, Iceland; 582 Department of Health and Behavior Studies, Columbia University, New York, NY, USA; 583 Department of Forensic Medicine, Kathmandu University, Dhulikhel, Nepal; 584 Department of Medicine, University of Alabama at Birmingham, Birmingham, AL, USA; 585 Medicine Service, US Department of Veterans Affairs (VA), Birmingham, AL, USA; 586 Department of Epidemiology, School of Preventive Oncology, Patna, India; 587 Department of Epidemiology, Healis Sekhsaria Institute for Public Health, Mumbai, India; 588 2nd Department of Surgery-SUUB, Carol Davila University of Medicine and Pharmacy, Bucharest, Romania; 589 2nd Surgery Department, Bucharest Emergency Hospital, Bucharest, Romania; 590 Pain Management Research Institute (PMRI), Northern Clinical School, University of Sydney, St Leonards, NSW, Australia; 591 Michael J Cousins Pain Management & Research Centre, Northern Sydney Local Health District, St Leonards, NSW, Australia; 592 Department of Medical Surgical Nursing, Urmia University of Medical Science, Urmia, Iran; 593 Emergency Nursing Department, Semnan University of Medical Sciences, Semnan, Iran; 594 Department of Biostatistics, Hamedan University of Medical Sciences, Hamadan, Iran; 595 Hospital Universitario de la Princesa, Autonomous University of Madrid, Madrid, Spain; 596 Centro de Investigación Biomédica en Red Enfermedades Respiratorias (CIBERES), Madrid, Spain; 597 Department of Public Health, Arba Minch University, Arba Minch, Ethiopia; 598 Hull York Medical School, University of Hull, Hull City, UK; 599 Usher Institute of Population Health Sciences and Informatics, University of Edinburgh, Edinburgh, UK; 600 Department of Psychiatry and Mental Health, University of Cape Town, Cape Town, South Africa; 601 South African Medical Research Council, Cape Town, South Africa; 602 Department of Psychology, Deakin University, Burwood, VIC, Australia; 603 Department of Community Medicine, Ahmadu Bello University, Zaria, Nigeria; 604 Department of Agriculture and Food Systems, University of Melbourne, Melbourne, VIC, Australia; 605 Department of Criminology, Law and Society, University of California Irvine, Irvine, CA, USA; 606 Department of Medicine, University of Valencia, Valencia, Spain; 607 Carlos III Health Institute, Biomedical Research Networking Center for Mental Health Network (CiberSAM), Madrid, Spain; 608 School of Social Work, University of Illinois, Urbana, IL, USA; 609 Department of Public Health, Arbaminch College of Health Sciences, Arbaminch town sikela, Ethiopia; 610 Axum College of Health Science, mekelle, Ethiopia; 611 School of Midwifery, University of Gondar, Gondar, Ethiopia; 612 School of Public Health, University of Adelaide, Adelaide, SA, Australia; 613 Department of Environmental Health, Wollo University, Dessie, Ethiopia; 614 Department of Community and Family Medicine, Iran University of Medical Sciences, Tehran, Iran; 615 Department of Pharmacognosy, Mekelle University, Mekelle, Ethiopia; 616 Institute of Public Health, University of Gondar, Gondar, Ethiopia; 617 Department of Public Health, Adigrat University, Adigrat, Ethiopia; 618 Psychiatry Department, Hawassa University, Hawassa, Ethiopia; 619 Institute of Public Health, Jagiellonian University Medical College, Krakow, Poland; 620 The Agency for Health Technology Assessment and Tariff System, Warszawa, Poland; 621 Department of Health Economics, Hanoi Medical University, Hanoi, Vietnam; 622 Department of Molecular Medicine and Pathology, University of Auckland, Auckland, New Zealand; 623 Clinical Hematology and Toxicology, Military Medical University, Hanoi, Vietnam; 624 Department of Community Medicine, All India Institute of Medical Sciences, Nagpur, India; 625 Department of Psychiatry, Massachusetts General Hospital, Boston, MA, USA; 626 Mbarara University of Science and Technology, Mbarara, Uganda; 627 Lee Kong Chian School of Medicine, Nanyang Technological University, Singapore; 628 Institute of Soil and Environmental Sciences, A T Still University, Faisalabad, Pakistan; 629 Gomal Center of Biochemistry and Biotechnology, Gomal University, Dera Ismail Khan, Pakistan; 630 TB Culture Laboratory, Mufti Mehmood Memorial Teaching Hospital, Dera Ismail Khan, Pakistan; 631 Research Department, National Institute of Population Studies (NIPS), Islamabad, Pakistan; 632 Amity Institute of Biotechnology, Amity University Rajasthan, Jaipur, India; 633 Division of Health Sciences, University of Warwick, Coventry, UK; 634 Argentine Society of Medicine, Buenos Aires, Argentina; 635 Velez Sarsfield Hospital, Buenos Aires, Argentina; 636 UKK Institute, Tampere, Finland; 637 Raffles Neuroscience Centre, Raffles Hospital, Singapore; 638 Yong Loo Lin School of Medicine, National University of Singapore, Singapore; 639 Department of Medical and Surgical Sciences, University of Bologna, Bologna, Italy; 640 Occupational Health Unit, Sant’Orsola Malpighi Hospital, Bologna, Italy; 641 Department of Health Care Administration and Economics, National Research University Higher School of Economics, Moscow, Russia; 642 Foundation University Medical College, Foundation University, Islamabad, Pakistan; 643 Demographic Change and Ageing Research Area, Federal Institute for Population Research, Wiesbaden, Germany; 644 Center of Population and Health, Wiesbaden, Germany; 645 Department of Physical Therapy, Naresuan University, Meung District, Thailand; 646 Department of Human Anatomy, Histology, Embryology, Bahir Dar University, Bahir Dar, Ethiopia; 647 Department of Pharmacology and Toxicology, Mekelle University, Mekelle, Ethiopia; 648 Department of Pharmacology, Addis Ababa University, Addis ababa, Ethiopia; 649 Department of Nursing, Wollo University, Dessie, Ethiopia; 650 Department of Orthopaedics, Wenzhou Medical University, Wenzhou, China; 651 Medical Physics Department, Ahvaz Jundishapur University of Medical Sciences, Ahvaz, Iran; 652 Department of Preventive Medicine, Northwestern University, Chicago, IL, USA; 653 School of International Development and Global Studies, University of Ottawa, Ottawa ON, Canada; 654 Health Services Management Research Center, Kerman University of Medical Sciences, Kerman, Iran; 655 Department of Health Management, Policy and Economics, Kerman University of Medical Sciences, Kerman, Iran; 656 Centre for Suicide Research and Prevention, University of Hong Kong, Hong Kong, China; 657 Department of Social Work and Social Administration, University of Hong Kong, Hong Kong, China; 658 School of Allied Health Sciences, Addis Ababa University, Addis Ababa, Ethiopia; 659 Department of Psychopharmacology, National Center of Neurology and Psychiatry, Tokyo, Japan; 660 Department of Sociology, Yonsei University, Seoul, South Korea; 661 Department of Health Policy & Management, Jackson State University, Jackson, MS, USA; 662 School of Medicine, Tsinghua University, Beijing, China; 663 Department of Environmental Health, Mazandaran University of Medical Sciences, Sari, Iran; 664 Department of Environmental Health, Academy of Medical Science, Sari, Iran; 665 School of Public Health and Management, Hubei University of Medicine, Shiyan, China; 666 Department of Epidemiology and Biostatistics, Wuhan University, Wuhan, China; 667 Global Health Institute, Wuhan University, Wuhan, China; 668 Social Determinants of Health Research Center, Ardabil University of Medical Science, Ardabil, Iran; 669 Department of Epidemiology, University Hospital of Setif, Setif, Algeria; 670 Department of Medicine, School of Clinical Sciences at Monash Health, Monash University, Melbourne, VIC, Australia; 671 Student Research Committee, Babol University of Medical Sciences, Babol, Iran; 672 Department of Community Medicine, Ardabil University of Medical Science, Ardabil, Iran; 673 Department of Environment Health Engineering, Gonabad University of Medical Sciences, Gonabad, Iran; 674 Faculty of Medical Sciences, Department of Health Education, Tarbiat Modares University, Tehran, Iran; 675 Department of Preventive Medicine, Wuhan University, Wuhan, China; 676 School of Public Health, Wuhan University of Science and Technology, Wuhan, China; 677 Hubei Province Key Laboratory of Occupational Hazard Identification and Control, Wuhan University of Science and Technology, Wuhan, China; 678 Indian Institute of Public Health, Public Health Foundation of India, Gurugram, India; 679 National Drug and Alcohol Research Centre, University of New South Wales, Sydney, NSW, Australia; 680 Department of Community Medicine, University of Peradeniya, Peradeniya, Sri Lanka; 681 Faculty of Infectious and Tropical Diseases, London School of Hygiene & Tropical Medicine, London, UK

**Keywords:** burden of disease, global, descriptive epidemiology

## Abstract

**Background:**

Past research in population health trends has shown that injuries form a substantial burden of population health loss. Regular updates to injury burden assessments are critical. We report Global Burden of Disease (GBD) 2017 Study estimates on morbidity and mortality for all injuries.

**Methods:**

We reviewed results for injuries from the GBD 2017 study. GBD 2017 measured injury-specific mortality and years of life lost (YLLs) using the Cause of Death Ensemble model. To measure non-fatal injuries, GBD 2017 modelled injury-specific incidence and converted this to prevalence and years lived with disability (YLDs). YLLs and YLDs were summed to calculate disability-adjusted life years (DALYs).

**Findings:**

In 1990, there were 4 260 493 (4 085 700 to 4 396 138) injury deaths, which increased to 4 484 722 (4 332 010 to 4 585 554) deaths in 2017, while age-standardised mortality decreased from 1079 (1073 to 1086) to 738 (730 to 745) per 100 000. In 1990, there were 354 064 302 (95% uncertainty interval: 338 174 876 to 371 610 802) new cases of injury globally, which increased to 520 710 288 (493 430 247 to 547 988 635) new cases in 2017. During this time, age-standardised incidence decreased non-significantly from 6824 (6534 to 7147) to 6763 (6412 to 7118) per 100 000. Between 1990 and 2017, age-standardised DALYs decreased from 4947 (4655 to 5233) per 100 000 to 3267 (3058 to 3505).

**Interpretation:**

Injuries are an important cause of health loss globally, though mortality has declined between 1990 and 2017. Future research in injury burden should focus on prevention in high-burden populations, improving data collection and ensuring access to medical care.

## Introduction

Injury burden assessments are a critical component of population health measurement. Across the global landscape of population health research, injuries are unique in that they are almost universally avertable yet can cause death or disability at any age. Even common injuries such as concussion resulting from falls, violence or road injuries may cause longer term sequelae, and injuries such as spinal cord injuries or limb amputations can cause long-term disability.^1^ As a result, injuries are recognised as being a source of lost health and human capital that could be averted with improved safety and prevention programmes as well as ensuring access to care resources.^2^ Across geographies, certain injuries such as envenomation may be relevant in specific locations where venomous creatures live, while injuries such as those occurring from adverse medical events are an increasing area of research in higher income areas of the world.^3–5^ Bolstering such programmes, however, requires detailed measurement of when, where and to whom injuries are occurring, necessitating focused research studies to add insight and context to broader geographical trends. Across all domains of injury prevention research, it is important to measure the causes of injury, such as road injuries, and the resulting disability, such as fractures, burns or traumatic brain injury, that can occur as a result. Such detailed measurement lends perspective for understanding burden and anticipating resources needed to care for and hopefully prevent future injury burden. Detailed measurements and assessments of this nature are critical for empowering policy makers and health system planners to appropriately plan and invest for mitigating future health loss from injuries. Reducing injury burden is an important component in global efforts such as the Sustainable Development Goal 3 to ‘ensure healthy lives and promote well-being for all at all ages’.^6^

While some research has focused on a certain type of injury or outcome from injury or specific area of the world,^7–10^ it has become important in an era of more sophisticated population health measurement to measure health loss from injuries comprehensively with detailed fatal and non-fatal estimates for different ages, sexes, across time periods and accounting for multiple different types of morbidity that can occur in an injury. Previously published literature on global injury burden through 2015 has provided comprehensive measurements of health loss due to injuries but still require regular updates to help inform research and policy, as new years of estimates are added and as new injuries and injury outcomes are incorporated.^11^ Comprehensive research of this nature shows how injury burden varies dynamically by age, sex, year, area of the world and type of injury, and hence, it is important to maintain close monitoring of injury burden every year in all parts of the world. In addition, as new datasets and statistical modelling methods become available, producing regular updates to burden estimation also ensures that results are as accurate as possible.

While the burden of injuries is widely studied and monitored through various methods of research, the Global Burden of Diseases, Injuries, and Risk Factors (GBD) Study is the only study framework that routinely provides estimates of morbidity and mortality from an exhaustive list of injuries in all areas of the world across ages and sexes. The most recent update to GBD was published in 2018 and provided morbidity and mortality estimates for 30 mutually exclusive causes of injury for 195 countries from 1990 to 2017.^12–17^ As part of this regular update, new datasets on cause of death and incidence are incorporated into the study, and additional geographical detail is added to better measure heterogeneity in burden estimates at a subnational level. In addition, updates such as reporting both nature of injury and cause of injury (described in more detail below) are incorporated. In this study, we describe key components in the GBD injury methodology and provide results from key trends in injury burden in terms of incidence, prevalence, years lived with disability (YLDs), cause-specific mortality, years of life lost (YLLs) and disability-adjusted life years (DALYs) by country, age groups, sex, year and injury type.

## Methods

The methods and results in this study are the same as are provided in GBD capstone publications, and a detailed description of GBD data and methods used for all processes related to GBD 2017 is provided in associated studies.^12–17^ Overall, GBD methods are also summarised in online supplementary appendix 1. Below, we summarise the specific methods used for measurement of injuries morbidity and mortality in GBD 2017.

10.1136/injuryprev-2019-043494.supp1 Supplementary data

### Key components of GBD study design

The GBD study incorporates several key components to allow for internally consistent estimates across all burden measures and metrics. First, population is measured to ensure consistent denominators for all population-level measurement. Second, all-cause mortality is measured using demographic methods. Third, cause-specific mortality for a mutually exclusive, collectively exhaustive hierarchy of diseases and injuries is measured, such that every death has one underlying cause of death and such that estimates for every possible cause of death are included, which requires the use of residual causes like ‘other transport injuries’. This results in the sum of cause-specific mortality equalling total all-cause mortality. Fourth, non-fatal health loss is measured for individuals living with a disease or injury that detracts from their full health status. Fifth, a composite measure of mortality and morbidity is computed. These steps are conducted within an age, sex and location hierarchy constructed such that demographic detail is available but where all estimates are internally consistent with all other estimates. GBD produces estimates for all causes, ages, sexes, years and locations. Risk factors and attributable burden for different are also measured, but those results are not included in this study.

### Case definition and cause hierarchy

The GBD case definition for an injury death is a death where the injury was the underlying cause of death. For example, if an individual falls on ice and sustains an epidural haematoma and dies after a seizure, the fall is the underlying cause. If an individual sustains a myocardial infarction and then falls and sustains the same epidural haematoma, then the myocardial infarction is the underlying cause of death. For non-fatal injuries, we define a case as an injury that warranted medical care. For example, if an individual slips and falls but does not sustain any bodily injury, it is not considered an injury. Online supplementary appendix table 1 provides the International Classification of Disease (ICD) codes used to identify causes of injury.

10.1136/injuryprev-2019-043494.supp2 Supplementary data

### Cause-specific mortality estimation

Cause-specific mortality from injuries is measured using the Cause of Death Ensemble model (CODEm). CODEm is described in more detail elsewhere; a summary of its use for injuries is as follows.^18^ First, all available data that can be used for cause of death estimation are identified. For injuries, this includes vital registration, verbal autopsy, police records, mortuary data and census data. These data are processed for use in the GBD cause and demographic hierarchy via a series of data processing steps including a process whereby ill-defined causes of death are reassigned to true underlying causes of death, which is described in more detail elsewhere but essentially is the process by which ill-defined causes of death are reclassified to causes of death in the GBD cause hierarchy.^19 20^ Next, a cause-specific mortality model is developed for each one of the 30 different causes of injury. For example, falls are modelled differently than road injuries, though both use the same CODEm modelling architecture. For each cause of injury, covariates that may be associated with the cause are identified and added as candidate covariates. CODEm runs different combinations of models using different covariates and outcome variables, specifically cause fraction models and cause-specific mortality rate models. Ensembles of models are also conducted to test performance of overall models formed from submodels. Once all models have been run, the top-performing models are selected based on out-of-sample predictive validity, wherein the model makes predictions on data that were not included in developing the model. The top-performing models are then weighted according to performance, and the final estimates form the penultimate estimate for cause-specific mortality from that injury. Those estimates are then adjusted to fit within the all-cause mortality estimate, so that cause-specific deaths sum up to the overall mortality estimate for each population and demographic. YLLs are computed as the cause-specific mortality rate at a given age multiplied by the residual life expectancy at that age, which is based on the observed maximum global life expectancy.

### Non-fatal injury estimation

Non-fatal injury estimation is also described in more detail in GBD literature. Key components in this process are as follows. First, data on incidence of non-fatal injury causes (eg, road injuries) is obtained from the GBD collaborator network and other injury research groups and researchers around the world. Data are cleaned and organised according to GBD study guidelines. Next, incidence of each cause of injury is modelled in DisMod-MR 2.1, which is a Bayesian meta-analysis tool used extensively in GBD research. Incidence estimates of injuries requiring medical care for each cause of injury then stream through an analytical pipeline. During this process, injury incidence is split into inpatient and outpatient to account for the different severity that is expected to occur. The coefficient that determines this split is derived from locations where both inpatient and outpatient data are available. After this, we measure the proportion of each cause of injury that leads to one of 47 different natures of injury using clinical data where both cause and nature are coded as well a Dirichlet statistical modelling process. Based on these steps, the incidence of each cause is also split into incidence of each cause-nature, which is the proportion of a given cause’s incidence leading to some specific nature of injury being the most severe injury sustained as estimated by the Dirichlet regression. These estimates are then converted to short-term and long-term injuries based on probability of each injury becoming long term, as determined by long-term follow-up injury surveys.^21–27^ For short-term injuries, incidence is converted to prevalence based on multiplying incidence by an expected duration of injury as determined by physicians and injury experts involved in the GBD study. For long-term injuries, incidence is converted to prevalence using differential equations that take into account the increased mortality for certain types of injury, for example, traumatic brain injury.^1^ Disability weights as derived elsewhere in the GBD study are then used to measure disability based on nature of injury.^28^ These measures are then summed across natures of injury for each cause to calculate YLDs. Each of these steps is conducted for every cause, age, sex, year and location in the GBD study design. Associated literature provides more detail on each of these steps.^12–17^

### DALY measurement

DALYs are calculated by summing YLLs and YLDs for each cause, age, sex, year and location.

### Uncertainty measurement

Uncertainty is measured at each step of the analytical process based on the sample size, SE or original uncertainty interval (UI) from each input to the study. Uncertainty is propagated through each step of the analysis by maintaining distributions of 1000 draws on which each analytical step is conducted. Final 95% UIs are determined based on the 25th and 975th values of the ordered values across draws.

### Code and results

Steps of the analytical process were conducted in Python version 2.7, Stata V.13.1 or R version 3.3. All steps of the analytical process are available online at ghdx.healthdata.org. This study reports a subset of measures and metrics for every cause of injury. All results and results with additional detail by age, sex, year and location can be downloaded at ghdx.healthdata.org.

### Guidelines for Accurate and Transparent Health Estimates Reporting (GATHER) statement

This study is adherent with guidelines from the GATHER (described in more detail in online supplementary appendix 2).^29^

10.1136/injuryprev-2019-043494.supp3 Supplementary data

## Results

Online supplementary appendix table 2 shows age-standardised incidence, prevalence, YLDs, deaths, YLLs and DALYs in 2017 by country as well as percentage change and UI from 1990 for each metric. Online supplementary appendix table 3 shows all-age numbers (ie, not divided by population) of incidence, prevalence, YLDs, deaths, YLLs and DALYs in 2017 by country as well as percentage change from 1990 and UI for each metric. In some instances, the UI for the per cent change crosses zero, meaning that statistically there was no significant difference. Online supplementary appendix figures 1–6, show the incidence and mortality from transport injuries, unintentional injuries, and interpersonal violence and self-harm by country for 2017 as well as the percentage change for both incidence and mortality between 1990 and 2017. All other results including age-specific and sex-specific results can be viewed and downloaded via freely and publicly available tools at ghdx.healthdata.org.

10.1136/injuryprev-2019-043494.supp4 Supplementary data

10.1136/injuryprev-2019-043494.supp5 Supplementary data

10.1136/injuryprev-2019-043494.supp6 Supplementary data

10.1136/injuryprev-2019-043494.supp7 Supplementary data

10.1136/injuryprev-2019-043494.supp8 Supplementary data

10.1136/injuryprev-2019-043494.supp9 Supplementary data

10.1136/injuryprev-2019-043494.supp10 Supplementary data

10.1136/injuryprev-2019-043494.supp11 Supplementary data

### Global trends in overall injury burden

In terms of fatal outcomes, deaths due to all injuries increased from 4 260 493 (4 085 700 to 4 396 138) in 1990 to 4 484 722 (4 332 010 to 4 585 554) in 2017, while YLLs decreased from 232 104 206 (219 920 058 to 241 973 733) to 195 231 148 (188 807 653 to 199 825 464) and age-standardised mortality rates decreased from 1079 (1073 to 1086) to 738 (730 to 745) per 100 000. In terms of non-fatal outcomes, all-injury incidence (new cases) increased from 354 064 302 (338 174 876 to 371 610 802) in 1990 to 520 710 288 (493 430 247 to 547 988 635) in 2017, and YLDs increased from 37 452 031 (27 805 854 to 49 010 103) to 57 174 469 (42 073 855 to 75 427 036), while age-standardised incidence rates decreased non-significantly from 6824 (6534 to 7147) to 6763 (6412 to 7118) per 100 000. In terms of DALYs, age-standardised DALY rates decreased from 4947 (4655 to 5233) per 100 000 in 1990 to 3267 (3058 to 3505) in 2017.

**Figure 1:**
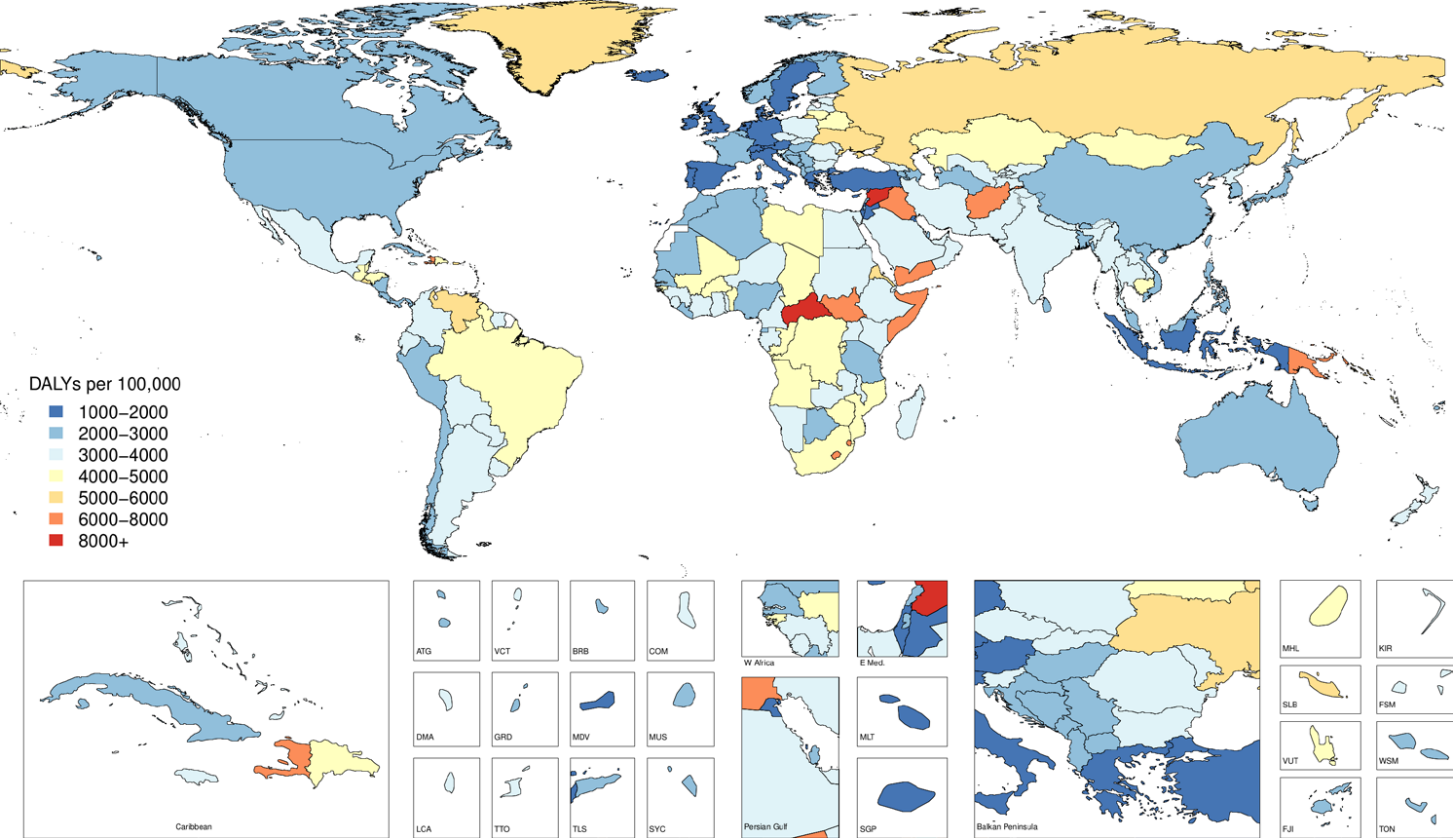
Age-standardised DALY rates by country, 2017. DALYs, disability-adjusted life years.

Figure 1 shows age-standardised DALY rates by country for 2017. While certain countries—specifically, Syria, Central African Republic and Iraq—have much higher DALY rates than most other countries, there still exists considerable heterogeneity across countries that are not among these countries with the highest burden. South Sudan, Somalia and Yemen have much higher injury burden than much of the rest of the world, for example, with age-standardised DALY rates of 7391.51 per 100 000 (6536.44 to 8440.14), 7364.66 per 100 000 (6143.11 to 8960.58) and 7297.88 per 100 000 (6525.7 to 8438.15), respectively. Papua New Guinea also demonstrates high all-injury burden with 6803.33 DALYs per 100 000 (5652.2 to 8040.89) in 2017.

**Figure 2:**
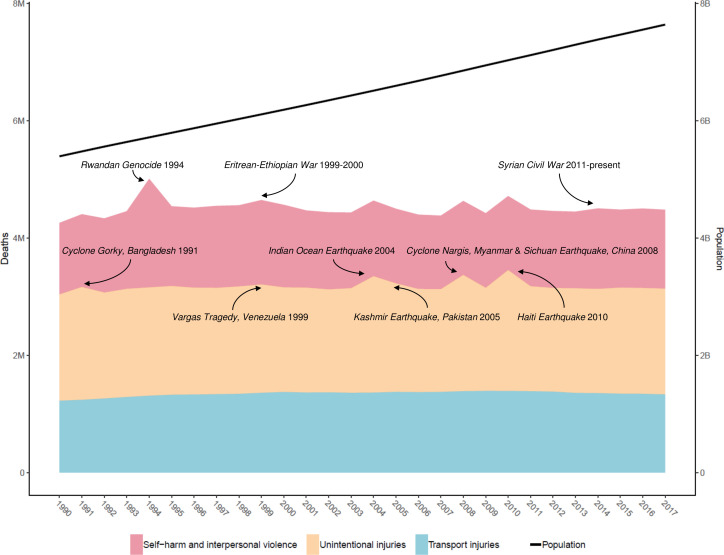
Global deaths for level 2 injuries and population from 1990 to 2017 with labelled fatal discontinuities.

Figure 2 presents deaths as a stacked graph for overall injury groups and population from 1990 to 2017 with labelled fatal discontinuities, defined as changes in deaths due to sudden, unexpected spikes in mortality that depart from the underlying mortality trend.^13^ Although population has steadily increased in the 28 years of the study, deaths per year due to injuries have remained relatively consistent over time. Natural disasters, such as earthquakes, have caused pronounced spikes in unintentional injuries deaths, while conflict and genocide have caused spikes in deaths in the interpersonal violence injury category.

### All-injury YLDs and YLLs by country in 2017

**Figure 3:**
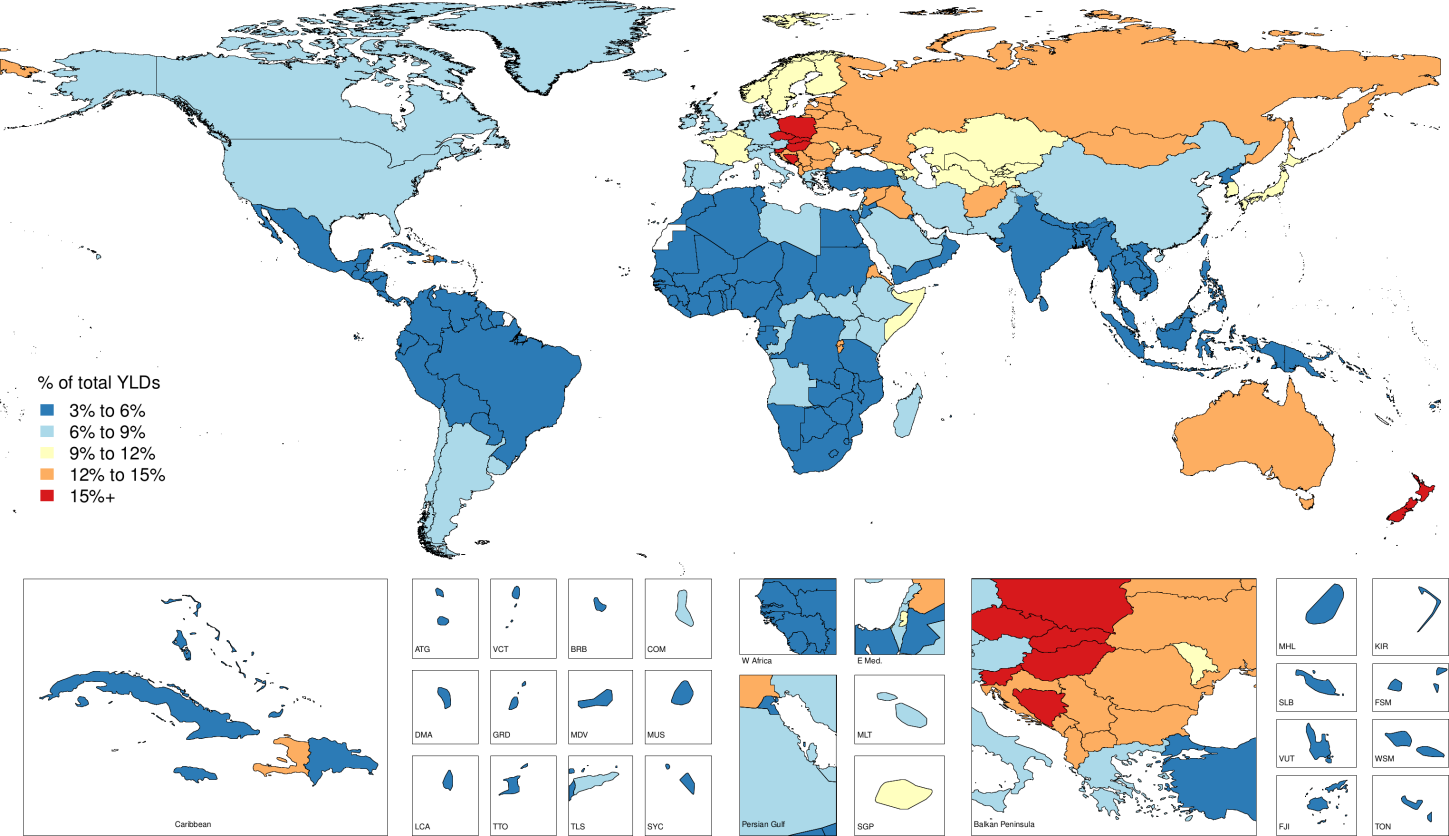
Percentage of YLDs in all ages due to injuries in 2017. YLDs, years lived with disability.

Figure 3 shows the percentage of total all-age, combined-sex YLDs by country in 2017. This figure shows several geographical patterns that help depict the non-fatal burden of injuries globally in terms of their relative contribution to overall disability. First, the percentage of total disability caused by injuries varies widely by country. Mauritius experiences only 3.04% (2.79% to 3.29%) of non-fatal burden from injuries, while Slovenia experiences 19.11% (17.11% to 21.27%) of non-fatal burden from injuries. In other words, if all disability in these two populations is combined in 2017, there is over sixfold variation in how much of this disability was caused by injuries. These patterns also reflect burden from non-injury conditions, since locations with higher burden from communicable disease may have correspondingly lower proportion due to injuries. As an extension of these geographical trends, this map makes it evident that there are striking regional patterns in non-fatal injury burden. Eastern and Central Europe and Central Asia as well as Australasia have a notably higher percentage of total non-fatal burden from injuries than countries in other regions, while these percentages are relatively lower in most areas of Africa, the Americas and areas of South, East and Southeast Asia. To some extent, this map also reflects the underlying burden from non-injury causes, too, since areas of the world with high non-fatal disability from conditions such as anaemia, communicable diseases and other types of health loss could have correspondingly higher percentages of disability from these conditions instead of injuries. This map also shows examples of positive deviations from global trends; Indonesia, for example, has a relatively low percentage of non-fatal health loss due to injuries compared with many other countries.

**Figure 4:**
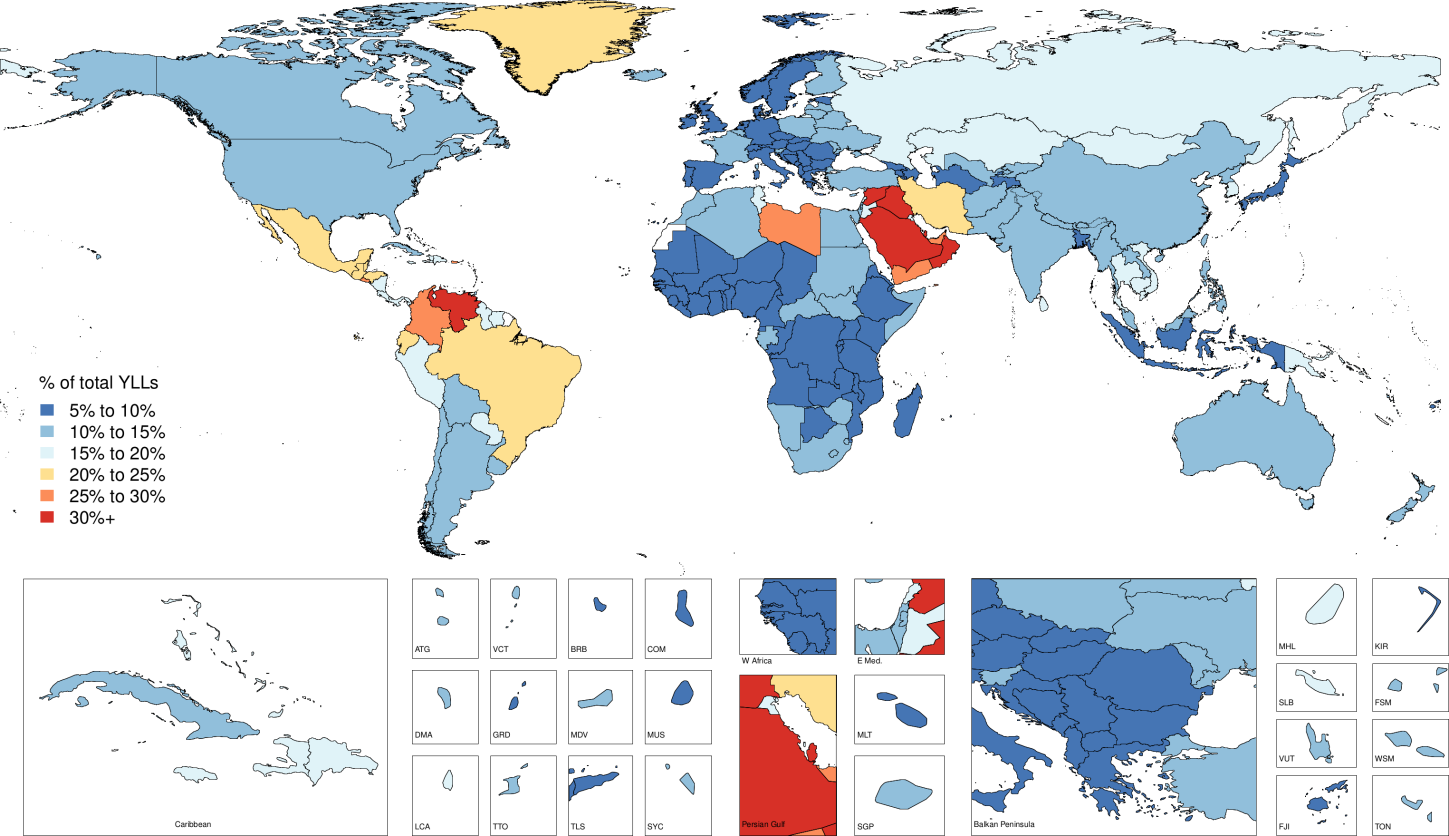
Percentage of YLLs in all ages due to injuries in 2017. YLLs, years of life lost.

Figure 4 similarly shows the percentage of total all-age, combined-sex YLLs by country in 2017. This figure interestingly shows how mortality patterns demonstrate different geographical trends than the non-fatal burden, as depicted in figure 2, though it should be noted that YLLs will also be disproportionately higher in younger populations, all else being equal. In particular, the locations with the highest percentage of YLLs due to injuries are in certain countries in North Africa and the Middle East, including Syria, where 59.51% (56.59% to 62.35%) of YLLs were due to injuries in 2017, and Iraq, where 41.34% of YLLs were due to injuries in 2017. Areas of Latin America including Venezuela, Honduras and Belize also have a relatively high percentage of total YLLs due to injuries. Conversely, certain areas of the world also demonstrate a relatively low percentage of total YLLs due to injuries, specifically, certain countries in Africa such as Nigeria and Madagascar have relatively lower percentages, though this also reflects relatively higher mortality from other non-injury causes in these countries.

### Cause-specific DALY rates by sex

**Figure 5:**
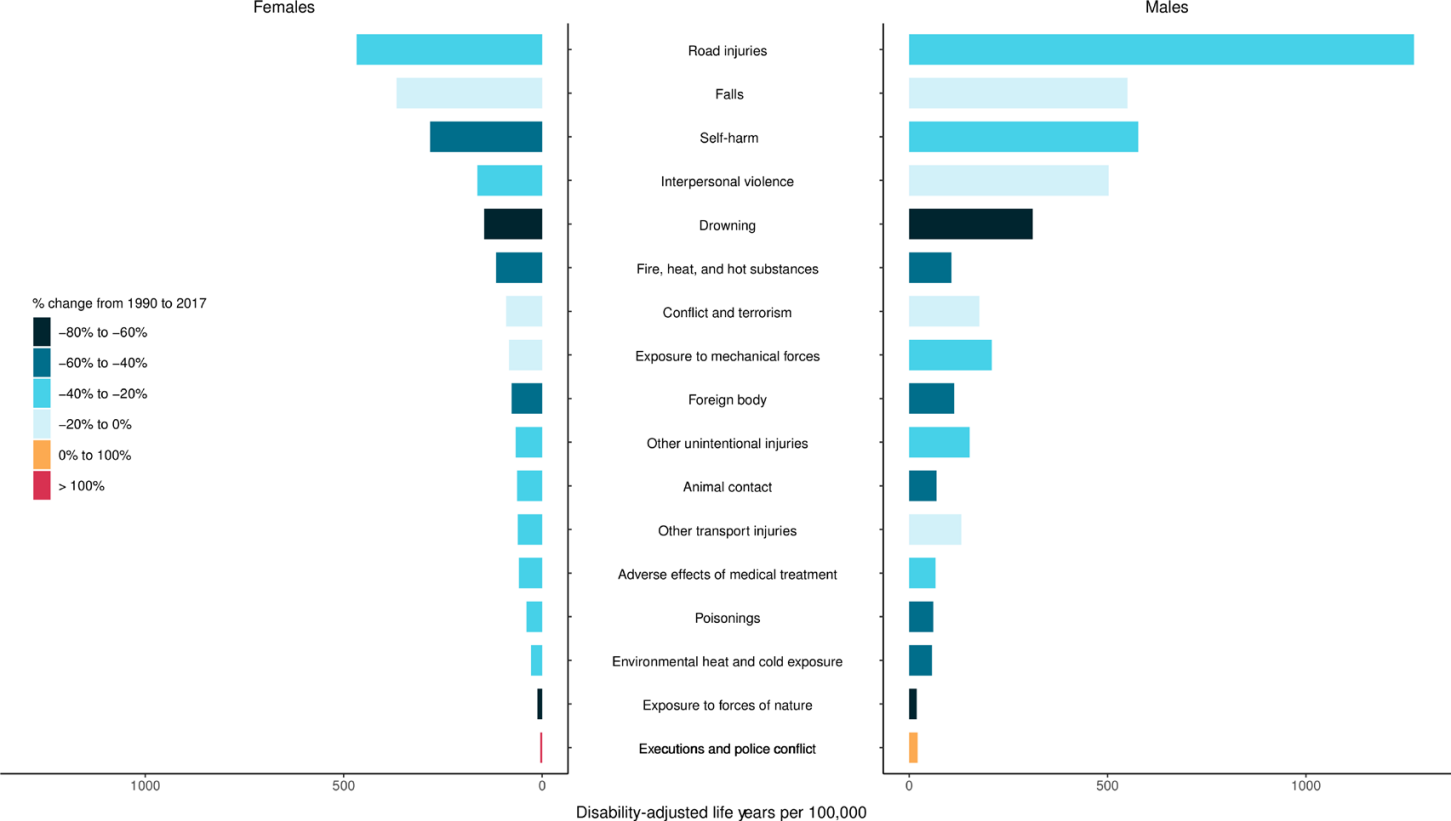
Age-standardised DALY rates by sex for injuries in level 3 of the GBD cause hierarchy in 2017 and percentage change from 1990 to 2017. DALY, disability-adjusted life year; GBD, Global Burden of Disease.

Figure 5 shows cause-specific DALY rates by sex for 17 injuries in 2017 as well as percentage change from 1990 to 2017 by cause and sex. The black and dark blue bars show causes with greater relative improvement over the time period of this study, while lighter blue, orange and red show injuries that have had lesser improvements, no improvements or increasing burden over time.

In 2017, men experienced higher age-standardised DALY rates than women for all injuries except fire, heat and hot substances. The most marked differences, where DALY rates for men are more than double those of women, can be seen in self-harm, interpersonal violence, road injuries, other transport injuries, exposure to mechanical forces, environmental heat and cold exposure, and executions and police violence. Road injuries (1272 (1209 to 1331) per 100 000), self-harm (577 (525 to 604)) and falls (550 (462 to 653)) were the causes with the highest DALY rates for men in 2017. Women had the highest DALY rates due to the same injuries, but at a lesser magnitude, with rates of 467 (432 to 502) per 100 000 for road injuries, 367 (304 to 442) for falls and 282 (268 to 293) for self-harm.

The causes with the largest decreases in DALY rates for men from 1990 to 2017 were exposure to forces of nature (72.4% (63.8% to 79.1%)), drowning (62.7% (58.8% to 65.4%)) and fire, heat and hot substances (43.6% (26.4% to 49.9%)). For women, exposure to forces of nature (72.8% (63.8% to 79.6%)), drowning (65.8% (58.6% to 69.2%)) and self-harm (50.8% (48.2% to 55.9%)) had the largest decreases in DALY rates. The only increases in DALY rates were seen in executions and police conflict for both women (298.0% (257.1% to 389.0%)) and men (46.4% (31.2% to 173.0%)).

### Comparative regional DALY rates in 2017

**Figure 6:**
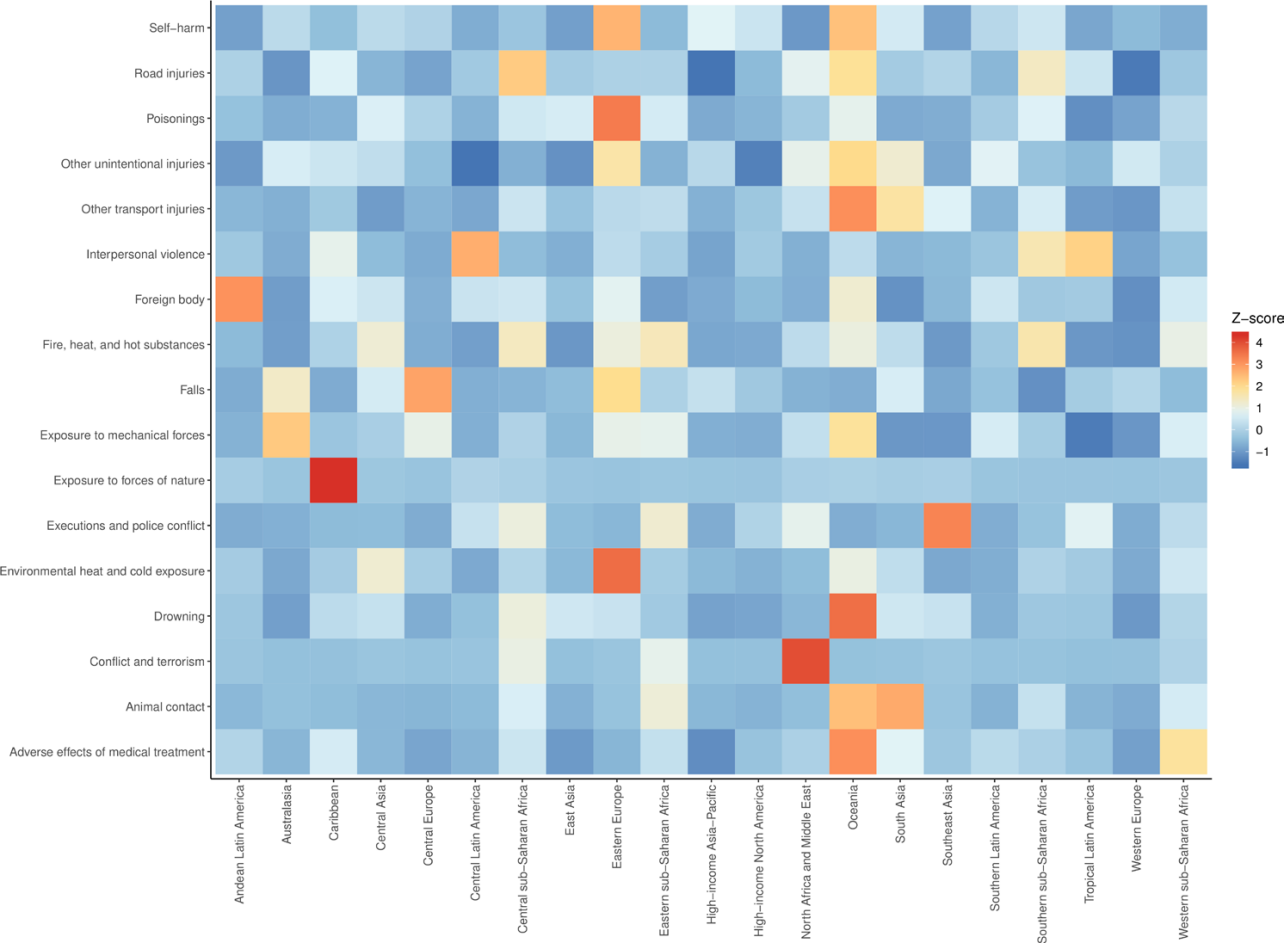
Heatmap showing the Z-score of age-standardised mean DALY rates for select injuries by GBD region in 2017. GBD, Global Burden of Disease.

Figure 6 shows a heatmap of the number of standard deviations (SD) above or below the mean of a row (ie, a Z-score) of age-standardised DALY rates for select injuries by GBD region in 2017. For example, the figure shows that the rate of age-standardised DALYs in Eastern Europe is approximately three SD higher than the across mean age-standardised DALY rates of environmental heat and cold exposure across all regions. Poisonings is also a cause with an age-standardised DALY rate that is approximately three SD higher than in other regions. Positive deviance is seen in high-income Asia Pacific for road injuries, where age-standardised DALYs are one SD lower than the mean across regions. Conversely, Central sub-Saharan Africa has age-standardised DALY rates that are two SD higher than the mean across regions. This figure also demonstrates how certain causes have relatively less variation across regions, for example, most regions do not deviate from the mean age-standardised DALY rates across regions for exposure to forces of nature, with the exception of the Caribbean, which had an age-standardised DALY rate that was approximately four SD above the mean across regions in 2017. Oceania and Eastern Europe stand out as having higher DALY rates for select injuries than other regions, while East Asia, high-income Asia Pacific, high-income North America, Western Europe and Southern Latin America experienced less than average burden of injuries in 2017.

## Discussion

Measuring, understanding and acting on the global burden of injuries should be considered a foundational component of population health research. While this study has reviewed injury burden trends from GBD 2017, it is also evident that these trends are sufficiently different by injury type and geography that it becomes difficult to succinctly generalise the findings in this study. Nevertheless, this study reveals themes and principles germane to the state of global injury burden in 2017 that are relevant to injury burden and prevention research.

First, it should be recognised that despite global population growth with increases in injury cases and deaths, age-standardised death rates from injuries declined from 1990 to 2017. More research into successful improvements for specific injuries in specific countries should be more investigated to help guide efforts towards future improvements. In general terms, the reduction in injury mortality likely represent the combined effects of improvements in healthcare systems, investments in injury prevention programmes and, in certain circumstances, safety improvement such as vehicle safety testing, helmet, seatbelt and drinking and driving laws. While burden trends across all diseases and injuries vary by geography and time, these improvements in injury burden are generally consistent with reporting of communicable and non-communicable disease trends reported in GBD 2017.

Despite improvements in terms of rates, however, it is important to consider the impact of absolute injury burden in younger and adult ages on the social capital and workforce in a country. Second, in reviewing temporal trends in figure 2, it becomes evident that war and conflict and environmental disasters can cause profound increases in deaths over a short period of time. This unfortunate and tragic reality should be made more broadly visible as issues such as war, conflict and climate change continue to threaten the populations of the 21st century. Third, sex differentials in the burden of different injury types are large, with men experiencing significantly higher burden from the four leading causes of injury DALYs in 2017. Preventive research and focused interventions into why this is occurring in road injuries, falls, self-harm, interpersonal violence and drowning is critical. It is also critical to address injuries such as fire, heat and hot substance and sexual violence where females experience greater burden and to better understand the factors that drive sex differences. As a fourth theme, we observed that there are cases of both positive and negative deviance from cross-region trends for each injury, as shown in figure 6, which appear to occur even outside of expected differences by income group. For example, understanding why high-income Asia Pacific and Western Europe are performing better than high-income North America in road injury burden could help improve road injury burden even in this higher income setting.

Beyond these four themes, there are evidently a great deal of nuances and specific outcomes to measure and understand in future injury research. While every cause of health loss in a population is important to measure and understand, injuries are unique in that understanding burden requires investigation of an array of circumstances such as infrastructure, the built environment, rates of interpersonal violence in a population and individual behaviours such as alcohol intoxication or drug use. The findings in this paper also demonstrate how it is critical to measure and understand the spectrum of health loss due to injuries ranging from relatively silent injuries to injuries that profoundly affect functional status. An incident as elemental as a trip and fall can lead to profoundly disabling health consequences such as spinal cord injury, which can have lifelong disability. The disability caused by shorter term injuries, such as an arm fracture, in addition to causing suffering and disability, can cause loss of human capital.^30^ While this study focused more on the causes of injury as defined in the GBD cause hierarchy, future GBD studies should focus also on depicting the distribution of nature of injury results to better understand how these types of disability affect an individual’s functional status. Such analyses become increasingly meaningful as research emerges on, for example, the increased risk of dementia that traumatic brain injury patients may experience.^31^ The findings in this paper also demonstrate how measuring injury burden necessitates review of the population factors that affect injury risk. For example, an event as disastrous as an earthquake may have radically different impacts on a population depending on infrastructure and access to care resources. Understanding how populations can protect themselves against future, unanticipated catastrophe could lead to averted death and disability in the future. As was shown in figure 2, catastrophic events both in terms of natural disasters and war and conflict can significantly add to the death and disability experienced by a population in a short period of time.

The geographical trends shown in this paper are also critical to review and understand by the broader global health community. As shown in figure 6, considerable heterogeneity exists across regions for certain causes. While vehicles were driven in nearly every populated area of earth in 2017, this study shows that different regions of the world have markedly different rates of death and disability resulting from road injuries, underscoring the importance of measuring and understanding the effects of specific factors on injury burden.^32^ It is not necessarily surprising to observe that countries or regions with relatively lower healthcare access and quality, less road safety infrastructure and lower utilisation of vehicles with modern safety standards would have higher rates of road injuries DALYs. The question that extends from this observation, however, is the extent to which burden from this type of injury cause could be avoided were every country to have the safety and prevention factors available in higher income settings. The injury and safety research communities should consider future investigation of counterfactual analyses to better measure and understand the impact that road safety legislation, modernisation of roads and vehicles and improving first response medical care could have on road injury burden, as an example, though parallel examples can be developed for other injury causes as well. This research could help cost-effectiveness analyses and guide investment in safer infrastructure.

These observations converge on a common theme: much of the injury burden may be largely preventable and understanding the success or failure of different prevention efforts should be a prioritised area of health research. Moreover, it is critical for there to be continued engagement across different areas of the world for the purposes of discussing effective and ineffective injury prevention strategies. Dialogue focused on findings across injury prevention efforts via forums such as global safety conferences as well as studies published in research journals should continue to help policy makers and public health planners make strategic investments for preventing future injury burden.^33^ In addition, more research into the cause of injury and resulting bodily injury and environmental and contextual features where injuries occur such type of road in a road injury or fires in factories versus in residences may provide further insight into preventing future injury burden.

Known limitations of injury burden estimation in the GBD framework have been reported previously in peer-reviewed literature.^1 11 13 16^ Generally, identified limitations include data sparsity and correspondingly greater uncertainty in certain geographies, limited geographical coverage of data informing long-term disability estimates and cause–nature relationships, and potential reporting biases for injuries such as self-harm and interpersonal violence. These limitations have been discussed in the aforementioned literature, and this overview study was additionally limited in scope due to the extensive size of the GBD cause hierarchy and location hierarchy. Indeed, over 1400 different cause–nature combinations are available for reporting in the GBD cause hierarchy, and future research would benefit from examining results in the detailed cause hierarchy and across the detailed location, age and sex hierarchy. The GBD Study platform and collaborator network provide a constructive collaborative platform on which future assessments can be conducted and published.

## Conclusion

Injury burden is complex but foundational in formulating global health loss. We have identified four broad trends in global injury burden that converge on the principle that injuries should be considered largely preventable but that detailed burden estimates through recent years are a critical global resource to inform meaningful policy. It will be important accurate measurement to continue into the future to guide injury prevention policy.

**What is already known on the subject**

- Injury burden globally varies across many dimensions but remains as an important component of global health loss. Regular updates in injury burden measurement are critical.
- Injuries can be largely preventable, but prevention efforts must be guided by up-to-date estimates of injury burden that can be used on an age-specific, sex-specific, year-specific, location-specific and injury-specific basis.

**What this study adds**

- This study incorporates updated data and methods that were used in Global Burden of Disease 2017 with updated burden estimates for the year 2017, as well as newly available results in terms of nature of injury.
- Global age-standardised mortality and disability-adjusted life years decreased between 1990 and 2017. Decreases in age-standardised incidence were not statistically significant.
- Trends over time vary depending on the specific injury, sex and location.
- Injury burden in a population can be radically affected by war, civil conflict and natural disasters.
