# Correction: *Global injury morbidity and mortality from 1990 to 2017: results from the Global Burden of Disease Study 2017*

**DOI:** 10.1136/injuryprev-2019-043494corr1

**Published:** 2020-09-28

James SL, Castle CD, Dingels ZV, *et al*. Global injury morbidity and mortality from 1990 to 2017: results from the Global Burden of Disease Study 2017. *Inj Prev*. 2020. doi: 10.1136/injuryprev-2019-043494

This article was previously published with errors in affiliations for authors Serge Resnikof and Aziz Rezapour. The updated affiliations are below

Serge Resnikoff ^521, 522^

^521^Brien Holden Vision Institute, Sydney, NSW, Australia

^522^Organization for the Prevention of Blindness, Paris, France

Aziz Rezapour^30^

^30^Health Management and Economics Research Center, Iran University of Medical Sciences, Tehran, Iran

