# Supplementary material for: Global injury morbidity and mortality from 1990 to 2017: results from the Global Burden of Disease Study 2017
Source: Inj Prev. 2020 Apr 24;26(Suppl 1):i96–i114. doi: 10.1136/injuryprev-2019-043494 (PMC7571366; doi:10.1136/injuryprev-2019-043494)
Supplement: Supplementary data [file injuryprev-2019-043494supp002.pdf]

Appendix Table 1: List of International Classification of Diseases (ICD) codes mapped to the Global Burden of Disease cause list

Cause	Non-fatal case definition	ICD9	ICD9 Used in Hospital/Claims Analyses	ICD10	ICD10 Used in Hospital/Claims Analyses
Road injuries	Interaction with an automobile, motorcycle, pedal cycle, or other vehicles resulting in bodily damage.	E800.3, E801.3, E802.3, E803.3, E804.3, E805.3, E806.3, E807.3, E810.0-E810.6, E811.0-E811.7, E812.0-E812.7, E813.0-E813.7, E814.0-E814.7, E815.0-E815.7, E816.0-E816.7, E817.0-E817.7, E818.0-E818.7, E819.0-E819.7, E820.0-E820.6, E821.0-E821.6, E822.0-E822.7, E823.0-E823.7, E824.0-E824.7, E825.0-E825.7, E826.0-E826.1, E826.3-E826.4, E827.0, E827.3-E827.4, E828.0, E828.4, E829.0-E829.4	E800.3, E801.3, E802.3, E803.3, E804.3, E805.3, E806.3, E807.3-E810.6, E811.0-E820.6, E821.0-E821.6, E822.0-E826.1, E826.3-E827.0, E827.3-E828.0, E828.4-E829.4	V01-V04.99, V06-V80.929, V82-V82.9, V87.2-V87.3	V01-V04.99, V06-V80.929, V82-V82.9, V87.2-V87.3
Pedestrian road injuries	Interaction, as a pedestrian on the road, with an automobile, motorcycle, pedal cycle, or other vehicles resulting in bodily damage.	E811.7, E812.7, E813.7, E814.7, E815.7, E816.7, E817.7, E818.7, E819.7, E822.7, E823.7, E824.7, E825.7, E826.0, E827.0, E828.0, E829.0	E811.7, E812.7, E813.7, E814.7, E815.7, E816.7, E817.7, E818.7, E819.7, E822.7, E823.7, E824.7, E825.7-E826.0, E827.0, E828.0, E829.0	V01-V04.99, V06-V09.9	V01-V04.99, V06-V09.9
Cyclist road injuries	Accident, as a cyclist or passenger on a pedal cycle, resulting in bodily damage.	E800.3, E801.3, E802.3, E803.3, E804.3, E805.3, E806.3, E807.3, E810.6, E811.6, E812.6, E813.6, E814.6, E815.6, E816.6, E817.6, E818.6, E819.6, E820.6, E821.6, E822.6, E823.6, E824.6, E825.6, E826.1	E800.3, E801.3, E802.3, E803.3, E804.3, E805.3, E806.3, E807.3, E810.6, E811.6, E812.6, E813.6, E814.6, E815.6, E816.6, E817.6, E818.6, E819.6, E820.6, E821.6, E822.6, E823.6, E824.6, E825.6, E826.1	V10-V19.9	V10-V19.9
Motorcyclist road injuries	Accident, as a rider on a motorcycle, resulting in bodily damage.	E810.2-E810.3, E811.2-E811.3, E812.2-E812.3, E813.2-E813.3, E814.2-E814.3, E815.2-E815.3, E816.2-E816.3, E817.2-E817.3, E818.2-E818.3, E819.2-E819.3, E820.2-E820.3, E821.2-E821.3, E822.2-E822.3, E823.2-E823.3, E824.2-E824.3, E825.2-E825.3	E810.2-E810.3, E811.2-E811.3, E812.2-E812.3, E813.2-E813.3, E814.2-E814.3, E815.2-E815.3, E816.2-E816.3, E817.2-E817.3, E818.2-E818.3, E819.2-E819.3, E820.2-E820.3, E821.2-E821.3, E822.2-E822.3, E823.2-E823.3, E824.2-E824.3, E825.2-E825.3	V20-V29.9	V20-V29.9
Motor vehicle road injuries	Accident, as a driver or passenger in a motor vehicle, resulting in bodily damage.	E810.0-E810.1, E811.0-E811.1, E812.0-E812.1, E813.0-E813.1, E814.0-E814.1, E815.0-E815.1, E816.0-E816.1, E817.0-E817.1, E818.0-E818.1, E819.0-E819.1, E820.0-E820.1, E821.0-E821.1, E822.0-E822.1, E823.0-E823.1, E824.0-E824.1, E825.0-E825.1	E810.0-E810.1, E811.0-E811.1, E812.0-E812.1, E813.0-E813.1, E814.0-E814.1, E815.0-E815.1, E816.0-E816.1, E817.0-E817.1, E818.0-E818.1, E819.0-E819.1, E820.0-E820.1, E821.0-E821.1, E822.0-E822.1, E823.0-E823.1, E824.0-E824.1, E825.0-E825.1	V30-V79.9, V87.2-V87.3	V30-V79.9, V87.2-V87.3
Other road injuries	Bodily damage resulting from being a driver or passenger of a vehicle not including automobiles, motorcycles, bicycles (i.e. streetcar).	E810.4-E810.5, E811.4-E811.5, E812.4-E812.5, E813.4-E813.5, E814.4-E814.5, E815.4-E815.5, E816.4-E816.5, E817.4-E817.5, E818.4-E818.5, E819.4-E819.5, E820.4-E820.5, E821.4-E821.5, E822.4-E822.5, E823.4-E823.5, E824.4-E824.5, E825.4-E825.5, E826.3-E826.4, E827.3-E827.4, E828.4, E829.4	E810.4-E810.5, E811.4-E811.5, E812.4-E812.5, E813.4-E813.5, E814.4-E814.5, E815.4-E815.5, E816.4-E816.5, E817.4-E817.5, E818.4-E818.5, E819.4-E819.5, E820.4-E820.5, E821.4-E821.5, E822.4-E822.5, E823.4-E823.5, E824.4-E824.5, E825.4-E825.5, E826.3-E826.4, E827.3-E827.4, E828.4, E829.4	V80-V80.929, V82-V82.9	V80-V80.929, V82-V82.9
Other transport injuries	Interaction with a means of transport other than automobile, motorcycle, pedal cycle, or other road vehicles resulting in bodily damage.	E800-E800.2, E801-E801.2, E802-E802.2, E803-E803.2, E804-E804.2, E805-E805.2, E806-E806.2, E807-E807.2, E810.7, E820.7, E821.7, E826.2, E827.2, E828.2, E830-E838.9, E840-E849.9, E929.1	E800-E800.2, E801-E801.2, E802-E802.2, E803-E803.2, E804-E804.2, E805-E805.2, E806-E806.2, E807-E807.2, E810.7, E820.7, E821.7, E826.2, E827.2, E828.2, E830-E849.9, E929.1	V00-V00.898, V05-V05.99, V81-V81.9, V83-V86.99, V88.2-V88.3, V90-V98.8	V00-V00.898, V05-V05.99, V81-V81.9, V83-V86.99, V88.2-V98.8
Falls	A sudden movement downward due to slipping, tripping, or other accidental movement which results in a person coming to rest inadvertently on the ground, floor or other lower level, resulting in tissue damage.	E880-E886.99, E888-E888.9, E929.3	E880-E888.9, E929.3	W00-W19.9	W00-W19.9
Drowning	Non-fatal immersion or submersion in water or another fluid, regardless of whether tissue damage has occurred. The subject can be resuscitated and has not suffered brain death.	E910-E910.99	E910-E910.99	W65-W70.9, W73-W74.9	W65-W74.9
Fire, heat, and hot substances	Non-fatal exposure to fire, heat, or hot substance which results in tissue damage (need not be a burn).	E890-E899.09, E924-E924.99, E929.4	E890-E899.09, E924-E924.99, E929.4	X00-X06.9, X08-X19.9	X00-X19.9
Poisonings	Accidental exposure to a non-infectious substance which contacts the body or enters into the body via inhalation, ingestion, injection, or absorption and causes deranged physiological function of body and/or cellular injury/death.	E850.3-E858.99, E862-E869.99, E929.2	E856-E869.99	J70.5, X40-X44.9, X47-X49.9, Y10-Y14.9, Y16-Y19.9	J70.5, X46-X48.9
Poisoning by carbon monoxide	Accidental exposure to carbon monoxide (CO) as identified in a person who does not smoke, or a child (age < 14 years) whose smoking status is unknown, and has a carboxyhemoglobin (COHb) level of ≥ 5.0% as measured in a blood sample; OR a person who smokes, or a person (age ≥ 14 years) whose smoking status is unknown, with a carboxyhemoglobin (COHb) level of > 12.0% as measured in a blood sample; OR a person who smokes, or whose smoking status is unknown (age ≥ 14 years), and has a carboxyhemoglobin (COHb) level of ≥ 9.0% and ≤ 12.0% as measured in a blood sample; OR a person who was present and exposed in the same CO exposure event as that of a confirmed CO poisoning case.	E862-E862.99, E868-E869.99	E862-E862.99, E868-E868.99, E869.90-E869.99	J70.5, X47-X47.9	J70.5, X47-X47.9
Poisoning by other means	Accidental exposure to a non-infectious substance (other than carbon monoxide) which contacts the body or enters into the body via inhalation, ingestion, injection, or absorption and causes deranged physiological function of body and/or cellular injury/death.	E850.3-E858.99, E863-E866.99	E856-E861.99, E863-E867.09, E869-E869.9	X40-X44.9, X48-X49.9, Y10-Y14.9, Y16-Y19.9	X46-X46.9, X48-X48.9
Exposure to mechanical forces	Unintentional bodily damage resulting from contact with or threat of an (in)animate object, human, or plant.	E916-E922.99, E928.1-E928.7	E916-E922.99, E928.1-E928.7	W20-W38.9, W40-W43.9, W45.0-W45.2, W46-W46.2, W49-W52	W20-W38.9, W40-W43.9, W45.0-W45.2, W46-W52
Unintentional firearm injuries	Unintentional bodily damage resulting from contact with a firearm.	E922-E922.99, E928.7	E922-E922.99, E928.7	W32-W34.9	W32-W34.9
Other exposure to mechanical forces	Unintentional bodily damage resulting from contact with or threat of an (in)animate object (not including a firearm), human, or plant.	E916-E921.99, E928.1-E928.6	E916-E921.99, E928.1-E928.6	W20-W31.9, W35-W38.9, W40-W43.9, W45.0-W45.2, W46-W46.2, W49-W52	W20-W31.9, W35-W38.9, W40-W43.9, W45.0-W45.2, W46-W52
Adverse effects of medical treatment	Unintentional harm done to an individual as a result of medical treatment, where the individual would not have been harmed if he or she had not been exposed to the treatment, regardless of whether the treatment resulted in improvements to the original indication for treatment.	244.0-244.1, 244.3, 331.81, 333.92, 349-349.9, 357.6, 359.24, 379.6-379.63, 440.3-440.32, 457.0, 458.2-458.29, 518.6, 519.00-519.1, 525.6-525.79, 526.62-526.63, 530.87, 536.4-536.49, 539-539.9, 551.2-551.29, 552.2-552.29, 553.2-553.29, 564.2-564.4, 569.6-569.8, 596.81-596.83, 598.2, 612-612.1, 780.66, 995.89, E870-E876.9, E878-E879.9, E930-E949.9, V44-V45, V45.2-V45.4, V45.7, V45.77, V45.79-V45.8, V45.87-V45.89	244.0-244.1, 244.3, 331.81, 333.92, 349-349.9, 357.6-359.24, 379.6-379.63, 440.3-440.32, 457.0, 458.2-458.29, 518.6-519.1, 525.6-525.79, 526.62-526.63, 530.87, 536.4-536.49, 539-539.9, 551.2-551.29, 552.2-552.29, 553.2-553.29, 564.2-564.4, 569.6-569.8, 596.81-596.83, 598.2, 612-612.1, 780.66, 995.89, E870-E879.9, E930-E949.9, V44-V45.89	D69.5-D69.59, D70.1, D78-D78.89, E03.2, E06.4, E09-E09.9, E16.0, E23.1, E24.2, E27.3, E36-E36.8, E66.1, E89-E89.9, G21.1-G21.19, G24.0-G24.09, G25.1, G25.4, G25.6-G25.79, G62.0, G72.0, G93.7, G96.0, G96.11, G97-G97.9, H59-H59.89, H91.0-H91.09, H95-H95.9, I95.2-I95.81, I97-I97.9, J70-J70.4, J95-J95.9, K08.5-K08.59, K43-K43.9, K62.7, K68.11, K91-K91.9, K94-K95.89, L23.3, L27.0-L27.1, L56.0-L56.1, L64.0, L76-L76.82, M10.2-M10.29, M87.1-M87.19, M96-M96.9, N14-N14.4, N46.021-N46.121, N52.2-N52.39, N65-N65.1, N99-N99.9, P93-P93.8, P96.2, Y40-Y84.9, Y88-Y88.3, Z21.0, Z42-Z43.0, Z43.8-Z48.9, Z51-Z51.9, Z88-Z94.0, Z94.6, Z94.8-Z94.9, Z96-Z97.2, Z97.8-Z99.12, Z99.3-Z99.9	D69.5-D69.59, D70.1, D78-D78.89, E03.2, E06.4, E09-E09.9, E16.0, E23.1, E24.2, E27.3, E36-E36.8, E66.1, E89-E89.9, G21.1-G25.79, G62.0-G97.9, H59-H59.89, H91.0-H91.09, H95-H95.9, I95.2-I97.9, J70-J70.4, J95-J95.9, K08.5-K08.59, K43-K43.9, K62.7, K68.11, K91-K91.9, K94-K95.89, L23.3, L27.0-L27.1, L56.0-L56.1, L64.0, L76-L76.82, M10.2-M10.29, M87.1-M87.19, M96-M96.9, N46.021-N46.121, N52.2-N52.39, N65-N65.1, N99-N99.9, P93-P93.8, P96.2, Y40-Y84.9, Y88-Y88.3, Z21.0, Z42-Z51.9, Z88-Z94.0, Z94.6-Z99.9
Animal contact	Bodily damage resulting from unintentionally being attacked, butted, impaled, bitten, stung, crushed, exposed to or stepped on by a non-human animal.	E905-E906.99	E905-E906.99	W52.0-W62.9, W64-W64.9, X20-X29.9	W52.0-W64.9, X20-X29.9
Venomous animal contact	Bodily damage resulting from unintentionally being bitten by, stung by, or exposed to a non-human venomous or poisonous animal.		E905-E905.99		W52.3, X20-X29.9

Appendix Table 1: List of International Classification of Diseases (ICD) codes mapped to the Global Burden of Disease cause list

Cause	Non-fatal case definition	ICD9	ICD9 Used in Hospital/Claims Analyses	ICD10	ICD10 Used in Hospital/Claims Analyses
Non-venomous animal contact	Bodily damage resulting from unintentionally being attacked, struck, impaled, crushed, exposed to or stepped on by a non-human animal.	E905-E906.99	E906-E906.99	W52.0-W62.9, W64-W64.9, X20-X29.9	W52.0-W52.2, W52.4-W64.9
Foreign body	Unintentional bodily damage from an extraneous material or substance being within the body, not including medical or surgical devices.	360.5-360.69, 374.86, 376.6, 709.4, 770.1-770.18, E911-E912.09, E913.8-E915.09		H02.81-H02.819, H44.6-H44.799, M60.2-M60.28, W44-W45, W45.3-W45.9, W78-W80.9, W83-W84.9	
Pulmonary aspiration and foreign body in airway	Unintentional bodily damage from inhaling, swallowing, or aspirating extraneous materials or substance that enters the airway or lungs.	770.1-770.18, E911-E912.09, E913.8-E913.99	770.1-770.18, E911-E913.19, E913.8-E913.99	W78-W80.9, W83-W84.9	W75-W76.9, W78-W80.9, W83-W84.9
Foreign body in eyes	Unintentional damage from extraneous materials or substance in the orbital structure or eye.	360.5-360.69, 374.86, 376.6, E914-E914.09	360.5-360.69, 374.86, 376.6, E914-E914.09	H02.81-H02.819, H44.6-H44.799	H02.81-H02.819, H44.6-H44.799
Foreign body in other body part	Unintentional bodily damage from an extraneous material or substance being within the body, not including the airway, lungs, or eyes.	709.4, E915-E915.09	709.4, E915-E915.09	M60.2-M60.28, W44-W45, W45.3-W45.9	M60.2-M60.28, W44-W45, W45.3-W45.9
Environmental heat and cold exposure	Tissue damage resulting from low or high temperatures existing in the non-indoors environment.	E900-E902.99, E926-E926.99, E929.5	E900-E902.99, E926-E926.99, E929.5	L55-L55.9, L58-L58.9, W88-W94.9, W97.9, W99-W99.9, X30-X32.9, X39-X39.9	L55-L55.9, L58-L58.9, W88-W99.9, X30-X32.9, X39-X39.9
Exposure to forces of nature	Bodily damage resulting from an unforeseen and often sudden natural event such as a hurricane, earthquake, tsunami, or tornado.	E907-E909.9		X33-X38.9	
Other unintentional injuries	Bodily damage that occurs by some external cause not including falls, poisoining, drowning, fire, heat, and hot substance, or other causes included in the GBD hierarchy.	E903-E904.99, E913.2-E913.39, E923-E923.99, E927-E928.09, E928.8-E928.89	E903-E904.99, E913.2-E913.39, E923-E923.99, E925-E925.99, E927-E928.09, E928.8-E928.89	W39-W39.9, W77-W77.9, W81-W81.9, X50-X58.9	W39-W39.9, W77-W77.9, W81-W81.9, W85-W87.9, X50-X58.9
Self-harm	Deliberate bodily damage inflicted on oneself with or without intent to kill oneself	E950-E959	E950-E959	X60-X64.9, X66-X84.9, Y87.0	X60-X64.9, X66-X84.9, Y87.0
Self-harm by firearm	Deliberate bodily damage inflicted on oneself by firearm with or without intent to kill oneself	E955-E955.9	E955-E955.9	X72-X74.9	X72-X74.9
Self-harm by other specified means	Deliberate bodily damage inflicted on oneself with or without intent to kill oneself by means of:* self-poisoning, medication overdose, transport incident, falling from height, hanging/strangulation *(not exhaustive)	E950-E954, E956-E959	E950-E954, E956-E959	X60-X64.9, X66-X71.9, X75-X84.9, Y87.0	X60-X64.9, X66-X71.9, X75-X84.9, Y87.0
Interpersonal violence	Sustaining bodily harm in terms of tissue damage from intentional use of physical force or power, threatened or actual, from another person or group not including military or police forces.	E960-E969	E960-E969	X85-Y08.9, Y87.1-Y87.2	T74.2-T76.22, X85-Y08.9, Y87.1-Y87.2
Physical violence by firearm	Sustaining bodily harm in terms of tissue damage from intentional use of physical force or power by a firearm from another person or group not including military or police forces.	E965-E965.4	E965-E965.4	X93-X95.9	X93-X95.9
Physical violence by sharp object	Sustaining bodily harm in terms of tissue damage from intentional use of physical force or power by a sharp object from another person or group not including military or police forces.	E966	E966	X99-X99.9	X99-X99.9
Sexual violence	For the sexual violence cause, we estimate the yearly prevalence of sexual violence, i.e., the proportion of the population that experienced at least one event of sexual violence in the last year. We define sexual violence as any sexual assault, including both penetrative sexual violence (rape) and non-penetrative sexual violence (other forms of unwanted sexual touching).	E960-E960.1		Y05-Y05.9	
Physical violence by other means	Sustaining bodily harm in terms of tissue damage from intentional use of physical force or power by an object other than a firearm or sharp object from another person or group not including military or police forces.	E961-E964, E965.5-E965.9, E967-E969	E961-E964, E965.5-E965.9, E967-E969	X85-X92.9, X96-X98.9, Y00-Y04.9, Y06-Y08.9, Y87.1-Y87.2	X85-X92.9, X96-X98.9, Y00-Y04.9, Y06-Y08.9, Y87.1-Y87.2
Conflict and terrorism	Bodily harm resulting from the instrumental use of violence by people who identify themselves as members of a group – whether this group is transitory or has a more permanent identity – against another group or set of individuals, in order to achieve political, economic or social objectives.	E979-E979.9, E990-E999.1		U00-U03, Y36-Y38.9, Y89.1	
Executions and police conflict	State-sanctioned executions or police-related altercations leading to bodily damage.	E970-E978		Y35-Y35.93, Y89.0	
