# Supplementary material for: Global injury morbidity and mortality from 1990 to 2017: results from the Global Burden of Disease Study 2017
Source: Inj Prev. 2020 Apr 24;26(Suppl 1):i96–i114. doi: 10.1136/injuryprev-2019-043494 (PMC7571366; doi:10.1136/injuryprev-2019-043494)
Supplement: Supplementary data [file injuryprev-2019-043494supp004.pdf]

Appendix Table 2: Age-standardised incidence, prevalence, YLDs, deaths, YLLs, and DALYs per 100 000 in 2017 and percent change between 1990 and 2017 for all injuries by location

Location	Incidence (95% UI)		Prevalence (95% UI)		Years lived with disability (95% UI)		Deaths (95% UI)		Years of life lost (95% UI)		Disability adjusted life years (95% UI)	
	2017 age-standardised rates per 100 000	Percentage change in age-standardised rates between 1990 and 2017	2017 age-standardised rates per 100 000	Percentage change in age-standardised rates between 1990 and 2017	2017 age-standardised rates per 100 000	Percentage change in age-standardised rates between 1990 and 2017	2017 age-standardised rates per 100 000	Percentage change in age-standardised rates between 1990 and 2017	2017 age-standardised rates per 100 000	Percentage change in age-standardised rates between 1990 and 2017	2017 age-standardised rates per 100 000	Percentage change in age-standardised rates between 1990 and 2017
**Global**	**6 763 (6 412 to 7 118)**	**-0.9 (-2.3 to 0.6)**	**18 927 (18 075 to 19 937)**	**-2.8 (-3.4 to -2.1)**	**719 (529 to 948)**	**-7.8 (-9.3 to -6.3)**	**58 (56 to 59)**	**-31.6 (-33.6 to -29.1)**	**2 548 (2 462 to 2 610)**	**-38.8 (-40.9 to -35.9)**	**3 267 (3 058 to 3 505)**	**-34.0 (-36.4 to -31.0)**
**Low SDI**	**6 081 (5 779 to 6 402)**	**-7.7 (-9.5 to -5.9)**	**17 161 (16 348 to 18 060)**	**6.8 (5.4 to 8.2)**	**768 (582 to 980)**	**-2.4 (-4.8 to 0.6)**	**80 (75 to 84)**	**-31.3 (-36.0 to -22.8)**	**3 218 (2 991 to 3 404)**	**-40.5 (-45.7 to -30.3)**	**3 987 (3 693 to 4 277)**	**-35.6 (-40.8 to -25.9)**
**Low-middle SDI**	**5 748 (5 474 to 6 048)**	**12.2 (10.3 to 14.2)**	**15 642 (14 944 to 16 436)**	**9.6 (8.6 to 10.7)**	**651 (489 to 839)**	**0.9 (-2.0 to 4.2)**	**77 (73 to 80)**	**-22.6 (-27.8 to -17.6)**	**3 155 (2 992 to 3 300)**	**-31.3 (-35.0 to -26.8)**	**3 805 (3 564 to 4 031)**	**-27.4 (-30.8 to -22.9)**
**Middle SDI**	**4 728 (4 486 to 4 978)**	**23.0 (20.8 to 25.4)**	**14 612 (13 957 to 15 306)**	**12.2 (10.7 to 13.8)**	**515 (383 to 674)**	**4.7 (0.7 to 9.1)**	**60 (57 to 61)**	**-32.8 (-35.2 to -30.3)**	**2 562 (2 490 to 2 619)**	**-40.9 (-42.9 to -39.0)**	**3 078 (2 918 to 3 257)**	**-36.3 (-38.4 to -33.9)**
**High-middle SDI**	**8 122 (7 661 to 8 643)**	**0.1 (-1.7 to 1.9)**	**22 979 (21 888 to 24 262)**	**-2.5 (-3.1 to -1.8)**	**798 (581 to 1 065)**	**-13.3 (-16.1 to -10.4)**	**49 (48 to 50)**	**-41.6 (-43.6 to -39.8)**	**2 121 (2 074 to 2 164)**	**-48.4 (-49.9 to -47.0)**	**2 919 (2 699 to 3 194)**	**-42.0 (-44.1 to -39.6)**
**High SDI**	**10 978 (10 315 to 11 722)**	**-6.4 (-8.3 to -4.4)**	**25 158 (23 749 to 26 884)**	**-8.7 (-9.3 to -8.1)**	**934 (671 to 1 265)**	**-9.5 (-10.3 to -8.8)**	**34 (33 to 34)**	**-36.1 (-37.0 to -35.1)**	**1 353 (1 333 to 1 374)**	**-42.5 (-43.4 to -41.6)**	**2 287 (2 027 to 2 618)**	**-32.4 (-34.6 to -30.1)**
**Central Europe, Eastern Europe, and Central Asia**	**15 640 (14 540 to 16 865)**	**-4.3 (-6.6 to -1.8)**	**36 441 (34 247 to 38 829)**	**-2.9 (-3.6 to -2.1)**	**1 432 (1 022 to 1 943)**	**-10.6 (-12.8 to -8.3)**	**64 (63 to 65)**	**-31.0 (-32.0 to -29.9)**	**2 893 (2 839 to 2 950)**	**-34.5 (-35.6 to -33.4)**	**4 325 (3 913 to 4 836)**	**-28.2 (-30.0 to -26.3)**
**Central Asia**	**10 307 (9 623 to 11 055)**	**-1.6 (-4.7 to 1.6)**	**26 513 (24 859 to 28 367)**	**-2.0 (-3.0 to -0.9)**	**1 050 (754 to 1 412)**	**-11.7 (-14.5 to -8.6)**	**48 (46 to 51)**	**-35.9 (-38.6 to -32.8)**	**2 303 (2 200 to 2 430)**	**-40.5 (-43.4 to -37.4)**	**3 353 (3 036 to 3 713)**	**-33.7 (-36.7 to -31.0)**
Armenia	10 159 (9 416 to 10 984)	-19.2 (-22.8 to -15.5)	28 197 (26 197 to 30 450)	-19.9 (-21.8 to -18.2)	1 027 (742 to 1 374)	-30.1 (-33.9 to -26.3)	32 (30 to 33)	-56.1 (-58.3 to -53.8)	1 311 (1 261 to 1 363)	-62.0 (-64.3 to -59.8)	2 338 (2 049 to 2 692)	-52.5 (-55.3 to -49.6)
Azerbaijan	10 236 (9 489 to 11 064)	3.7 (-0.5 to 8.1)	27 214 (25 418 to 29 158)	6.5 (4.3 to 8.9)	1 049 (756 to 1 410)	-9.0 (-13.2 to -4.4)	31 (28 to 35)	-46.7 (-53.0 to -37.9)	1 471 (1 311 to 1 686)	-51.6 (-57.7 to -44.0)	2 520 (2 177 to 2 923)	-39.9 (-45.2 to -33.6)
Georgia	10 832 (10 184 to 11 564)	-1.9 (-5.9 to 2.6)	26 776 (25 184 to 28 608)	-2.8 (-4.8 to -0.9)	1 034 (741 to 1 398)	-8.1 (-10.4 to -5.7)	52 (49 to 54)	-12.8 (-18.1 to -7.3)	2 300 (2 187 to 2 407)	-21.0 (-26.3 to -15.6)	3 334 (3 024 to 3 705)	-17.4 (-21.6 to -13.2)
Kazakhstan	11 583 (10 822 to 12 440)	-1.9 (-5.6 to 2.1)	27 993 (26 241 to 29 836)	-4.2 (-5.1 to -3.1)	1 074 (766 to 1 453)	-13.8 (-17.4 to -10.5)	74 (70 to 79)	-26.5 (-30.4 to -22.2)	3 501 (3 295 to 3 757)	-31.4 (-35.4 to -26.3)	4 574 (4 192 to 5 017)	-27.9 (-31.6 to -23.8)
Kyrgyzstan	9 318 (8 657 to 10 063)	-15.4 (-19.3 to -11.3)	24 211 (22 668 to 25 872)	-13.2 (-14.4 to -12.0)	978 (708 to 1 311)	-20.2 (-22.4 to -17.9)	47 (45 to 49)	-52.7 (-55.6 to -49.3)	2 199 (2 094 to 2 325)	-56.0 (-59.0 to -52.5)	3 177 (2 895 to 3 530)	-48.9 (-52.1 to -45.6)
Mongolia	11 980 (11 181 to 12 888)	14.5 (11.0 to 18.1)	30 038 (28 141 to 32 051)	12.7 (11.7 to 13.9)	1 293 (938 to 1 723)	-2.3 (-6.4 to 2.3)	72 (65 to 81)	-21.7 (-31.9 to -11.1)	3 610 (3 229 to 4 037)	-21.0 (-30.7 to -10.4)	4 903 (4 355 to 5 520)	-16.8 (-25.0 to -8.5)
Tajikistan	9 639 (8 960 to 10 444)	-1.6 (-4.9 to 1.9)	28 619 (26 575 to 30 924)	8.5 (4.2 to 14.5)	1 255 (934 to 1 647)	-1.8 (-6.8 to 5.2)	36 (33 to 40)	-37.4 (-43.4 to -28.9)	1 878 (1 715 to 2 095)	-38.6 (-44.4 to -31.7)	3 134 (2 764 to 3 542)	-27.7 (-32.8 to -22.0)
Turkmenistan	10 161 (9 426 to 10 990)	9.9 (5.5 to 14.0)	25 746 (24 064 to 27 631)	2.5 (1.2 to 3.9)	1 019 (735 to 1 371)	-13.4 (-17.7 to -8.8)	36 (33 to 39)	-44.8 (-50.0 to -39.0)	1 811 (1 650 to 2 006)	-49.4 (-54.9 to -42.8)	2 829 (2 497 to 3 229)	-40.5 (-45.6 to -35.1)
Uzbekistan	9 689 (9 010 to 10 436)	5.0 (0.9 to 9.0)	24 516 (22 938 to 26 311)	2.6 (1.5 to 3.8)	970 (696 to 1 308)	-9.1 (-12.6 to -5.4)	42 (37 to 46)	-33.0 (-40.5 to -25.0)	2 032 (1 811 to 2 267)	-38.4 (-45.5 to -31.0)	3 002 (2 641 to 3 406)	-31.2 (-37.4 to -25.5)
**Central Europe**	**19 299 (17 840 to 20 853)**	**-2.0 (-5.5 to 1.3)**	**41 104 (38 365 to 44 051)**	**-0.6 (-1.5 to 0.2)**	**1 681 (1 196 to 2 291)**	**-9.2 (-11.6 to -6.8)**	**38 (37 to 39)**	**-47.0 (-48.4 to -45.6)**	**1 490 (1 448 to 1 532)**	**-51.1 (-52.5 to -49.6)**	**3 171 (2 691 to 3 782)**	**-35.3 (-38.8 to -31.6)**
Albania	17 200 (15 807 to 18 775)	9.5 (5.5 to 13.6)	39 107 (36 411 to 42 038)	4.1 (2.5 to 5.7)	1 547 (1 101 to 2 124)	-4.6 (-8.8 to -0.4)	26 (22 to 31)	-37.1 (-47.5 to -25.8)	1 236 (1 037 to 1 454)	-41.7 (-51.5 to -31.2)	2 783 (2 272 to 3 380)	-25.6 (-33.0 to -18.7)
Bosnia and Herzegovina	17 598 (16 136 to 19 255)	25.0 (21.5 to 28.5)	43 988 (41 165 to 47 123)	31.0 (26.3 to 36.6)	1 780 (1 293 to 2 393)	20.5 (13.7 to 30.0)	25 (23 to 29)	-35.9 (-44.2 to -18.9)	1 026 (951 to 1 116)	-38.6 (-45.7 to -25.2)	2 806 (2 313 to 3 417)	-10.8 (-18.7 to -0.7)
Bulgaria	18 064 (16 684 to 19 630)	-0.5 (-4.8 to 4.2)	40 138 (37 387 to 43 082)	-3.0 (-4.0 to -1.8)	1 573 (1 111 to 2 152)	-9.6 (-11.8 to -7.4)	34 (32 to 37)	-41.4 (-44.9 to -37.8)	1 488 (1 393 to 1 595)	-45.3 (-48.8 to -41.6)	3 061 (2 595 to 3 640)	-31.3 (-35.3 to -27.7)
Croatia	15 872 (15 172 to 16 593)	-13.3 (-17.7 to -8.5)	33 606 (31 740 to 35 717)	-13.1 (-14.6 to -11.3)	1 389 (996 to 1 872)	-10.7 (-12.7 to -8.2)	38 (36 to 41)	-49.4 (-52.2 to -46.3)	1 223 (1 149 to 1 295)	-59.4 (-61.9 to -56.8)	2 612 (2 219 to 3 098)	-42.8 (-46.3 to -39.3)
Czech Republic	23 408 (21 492 to 25 619)	0.6 (-4.7 to 6.1)	47 907 (44 827 to 51 368)	8.8 (7.6 to 10.2)	2 009 (1 433 to 2 748)	3.3 (1.3 to 5.4)	35 (33 to 37)	-55.9 (-58.5 to -53.1)	1 280 (1 203 to 1 361)	-54.6 (-57.3 to -51.4)	3 289 (2 702 to 4 037)	-30.9 (-36.0 to -25.9)
Hungary	19 165 (17 537 to 20 958)	-14.0 (-18.8 to -9.2)	40 251 (37 382 to 43 440)	-3.0 (-4.6 to -1.0)	1 636 (1 166 to 2 240)	-15.0 (-17.5 to -12.5)	39 (37 to 41)	-63.0 (-65.0 to -60.9)	1 334 (1 260 to 1 414)	-64.4 (-66.4 to -62.2)	2 970 (2 484 to 3 567)	-47.6 (-51.7 to -43.5)
Macedonia	17 351 (15 857 to 18 960)	15.5 (12.3 to 18.7)	39 156 (36 299 to 42 267)	8.3 (6.5 to 9.8)	1 529 (1 088 to 2 101)	0.9 (-2.7 to 4.4)	24 (22 to 26)	-38.9 (-44.2 to -33.7)	997 (932 to 1 070)	-43.4 (-48.3 to -38.6)	2 526 (2 065 to 3 095)	-22.9 (-28.4 to -17.9)
Montenegro	18 290 (16 812 to 19 901)	3.2 (-0.1 to 6.7)	40 537 (37 724 to 43 565)	2.9 (1.6 to 4.3)	1 593 (1 133 to 2 174)	2.0 (0.2 to 3.7)	33 (30 to 36)	-31.3 (-37.5 to -24.4)	1 341 (1 221 to 1 468)	-40.0 (-45.7 to -33.6)	2 934 (2 443 to 3 547)	-22.7 (-28.0 to -17.9)
Poland	19 725 (18 196 to 21 388)	1.6 (-3.2 to 6.5)	41 015 (38 283 to 43 987)	0.4 (-0.6 to 1.4)	1 708 (1 217 to 2 332)	-8.3 (-11.4 to -5.3)	41 (39 to 44)	-40.9 (-44.2 to -37.2)	1 657 (1 563 to 1 760)	-46.0 (-49.3 to -42.5)	3 365 (2 865 to 3 992)	-31.8 (-35.8 to -28.0)
Romania	18 445 (17 003 to 19 967)	-10.3 (-14.5 to -5.5)	39 936 (37 302 to 42 894)	-11.7 (-13.1 to -10.1)	1 596 (1 131 to 2 193)	-23.0 (-26.1 to -20.0)	40 (38 to 42)	-43.7 (-46.7 to -40.7)	1 721 (1 630 to 1 823)	-50.9 (-53.6 to -48.1)	3 317 (2 838 to 3 909)	-40.6 (-43.8 to -37.5)
Serbia	17 446 (15 983 to 19 102)	11.0 (7.2 to 14.9)	40 205 (37 279 to 43 315)	9.8 (8.3 to 11.6)	1 566 (1 118 to 2 133)	4.5 (1.8 to 7.6)	33 (31 to 35)	-43.1 (-47.2 to -38.7)	1 213 (1 132 to 1 309)	-54.5 (-58.0 to -50.2)	2 779 (2 324 to 3 356)	-33.2 (-38.4 to -27.6)
Slovakia	19 987 (18 430 to 21 732)	-7.0 (-11.0 to -2.9)	41 271 (38 599 to 44 118)	-3.8 (-4.9 to -2.7)	1 716 (1 227 to 2 349)	-10.5 (-12.4 to -8.6)	38 (35 to 41)	-48.8 (-52.3 to -44.2)	1 468 (1 372 to 1 583)	-51.6 (-54.9 to -47.3)	3 184 (2 671 to 3 831)	-35.7 (-39.4 to -31.4)
Slovenia	23 879 (21 939 to 26 030)	-0.9 (-6.2 to 5.0)	46 153 (43 333 to 49 344)	3.1 (2.0 to 4.2)	2 034 (1 459 to 2 781)	1.4 (-0.0 to 2.8)	40 (37 to 42)	-52.3 (-55.5 to -48.9)	1 318 (1 222 to 1 409)	-59.5 (-62.6 to -56.5)	3 353 (2 772 to 4 094)	-36.3 (-41.6 to -31.6)
**Eastern Europe**	**16 136 (14 954 to 17 432)**	**-1.4 (-3.8 to 1.4)**	**37 660 (35 378 to 40 240)**	**-1.0 (-2.0 to 0.1)**	**1 423 (1 012 to 1 934)**	**-9.0 (-11.5 to -6.6)**	**83 (82 to 85)**	**-22.8 (-24.1 to -21.5)**	**3 845 (3 769 to 3 920)**	**-26.8 (-28.0 to -25.6)**	**5 268 (4 860 to 5 788)**	**-22.7 (-24.3 to -21.0)**
Belarus	15 996 (14 940 to 17 182)	4.3 (0.1 to 8.4)	37 285 (35 022 to 39 754)	1.3 (0.2 to 2.3)	1 401 (999 to 1 912)	-6.4 (-9.1 to -3.8)	65 (61 to 69)	-29.2 (-33.6 to -24.0)	2 753 (2 570 to 2 949)	-38.7 (-43.0 to -33.8)	4 154 (3 705 to 4 682)	-30.7 (-34.5 to -26.4)
Estonia	15 658 (14 553 to 16 880)	-16.0 (-20.4 to -11.7)	37 174 (34 852 to 39 737)	-9.3 (-10.8 to -7.7)	1 400 (996 to 1 904)	-18.3 (-20.0 to -16.7)	42 (37 to 48)	-65.9 (-70.3 to -61.1)	1 771 (1 548 to 2 018)	-69.8 (-73.6 to -65.5)	3 171 (2 703 to 3 730)	-58.2 (-62.4 to -53.7)
Latvia	16 135 (15 028 to 17 378)	-17.2 (-21.1 to -12.8)	36 743 (34 568 to 39 173)	-11.9 (-13.3 to -10.3)	1 402 (996 to 1 910)	-21.5 (-23.4 to -19.6)	61 (54 to 68)	-52.1 (-57.5 to -46.4)	2 572 (2 282 to 2 884)	-56.7 (-61.6 to -51.5)	3 974 (3 443 to 4 553)	-48.6 (-53.3 to -44.1)
Lithuania	17 107 (15 939 to 18 313)	-4.1 (-8.3 to 0.4)	38 251 (36 069 to 40 632)	-2.6 (-3.9 to -1.3)	1 482 (1 053 to 2 016)	-9.3 (-11.2 to -7.4)	75 (71 to 80)	-33.1 (-37.3 to -28.5)	3 140 (2 945 to 3 369)	-40.2 (-44.2 to -35.9)	4 622 (4 143 to 5 156)	-32.9 (-36.4 to -29.1)
Moldova	13 717 (12 751 to 14 806)	-11.3 (-15.3 to -6.9)	33 425 (31 254 to 35 842)	-9.8 (-11.1 to -8.0)	1 291 (927 to 1 754)	-19.1 (-21.8 to -16.5)	59 (56 to 61)	-42.4 (-45.1 to -39.6)	2 598 (2 475 to 2 735)	-49.6 (-52.7 to -46.0)	3 889 (3 518 to 4 320)	-42.4 (-45.3 to -39.3)
Russian Federation	16 338 (15 136 to 17 665)	-2.2 (-4.7 to 0.7)	38 053 (35 761 to 40 635)	-1.5 (-2.8 to -0.1)	1 444 (1 026 to 1 962)	-9.2 (-11.9 to -6.7)	87 (86 to 89)	-24.0 (-25.1 to -23.1)	3 987 (3 925 to 4 056)	-28.2 (-29.1 to -27.4)	5 431 (5 008 to 5 946)	-24.0 (-25.6 to -22.3)
Ukraine	15 672 (14 483 to 17 033)	2.2 (-0.6 to 5.3)	36 878 (34 570 to 39 431)	1.5 (0.6 to 2.4)	1 371 (971 to 1 872)	-7.5 (-10.1 to -4.8)	80 (76 to 85)	-11.6 (-16.7 to -6.0)	3 888 (3 681 to 4 115)	-12.7 (-17.9 to -6.9)	5 259 (4 810 to 5 777)	-11.4 (-15.3 to -7.0)
**High-income**	**10 528 (9 900 to 11 225)**	**-6.7 (-8.6 to -4.7)**	**23 823 (22 429 to 25 535)**	**-10.7 (-11.4 to -10.0)**	**884 (636 to 1 201)**	**-10.7 (-11.5 to -9.8)**	**35 (34 to 35)**	**-32.9 (-34.1 to -31.7)**	**1 411 (1 388 to 1 436)**	**-39.1 (-40.1 to -38.0)**	**2 295 (2 047 to 2 603)**	**-30.6 (-32.6 to -28.6)**
**Australasia**	**21 160 (19 626 to 22 751)**	**17.6 (14.6 to 20.9)**	**41 666 (38 504 to 45 847)**	**7.5 (6.0 to 9.3)**	**1 568 (1 114 to 2 129)**	**11.0 (9.5 to 12.5)**	**30 (28 to 33)**	**-33.8 (-39.4 to -28.3)**	**1 196 (1 090 to 1 305)**	**-44.3 (-49.2 to -39.3)**	**2 764 (2 291 to 3 350)**	**-22.3 (-28.2 to -17.1)**
Australia	20 376 (18 816 to 22 044)	18.1 (14.6 to 21.9)	40 557 (37 398 to 44 660)	8.4 (7.1 to 10.1)	1 514 (1 077 to 2 057)	11.6 (10.0 to 13.2)	30 (27 to 33)	-32.1 (-38.7 to -25.5)	1 156 (1 033 to 1 284)	-43.2 (-49.4 to -37.1)	2 670 (2 207 to 3 237)	-21.3 (-27.8 to -15.6)
New Zealand	25 354 (23 850 to 27 004)	17.3 (13.0 to 21.7)	47 680 (44 262 to 51 989)	4.7 (1.9 to 7.6)	1 860 (1 321 to 2 527)	9.9 (7.7 to 12.3)	32 (31 to 34)	-39.7 (-42.8 to -36.2)	1 410 (1 339 to 1 491)	-47.0 (-49.8 to -44.1)	3 270 (2 724 to 3 968)	-24.9 (-29.9 to -20.3)
**High-income Asia-Pacific**	**10 684 (9 942 to 11 555)**	**4.2 (1.6 to 6.8)**	**26 922 (25 148 to 29 002)**	**7.0 (6.0 to 8.2)**	**1 006 (718 to 1 365)**	**3.0 (1.4 to 4.6)**	**32 (31 to 34)**	**-36.0 (-38.3 to -33.5)**	**1 209 (1 168 to 1 256)**	**-45.6 (-47.7 to -43.4)**	**2 215 (1 941 to 2 568)**	**-30.8 (-34.3 to -27.2)**
Brunei	11 656 (10 908 to 12 515)	0.8 (-2.1 to 3.5)	27 837 (26 111 to 29 824)	-2.3 (-3.6 to -1.1)	1 031 (725 to 1 411)	-11.9 (-15.0 to -9.0)	44 (41 to 47)	-36.0 (-41.7 to -29.6)	1 839 (1 704 to 2 003)	-36.6 (-42.1 to -30.0)	2 871 (2 523 to 3 283)	-29.5 (-34.2 to -24.4)
Japan	10 978 (10 196 to 11 888)	13.1 (10.8 to 15.5)	28 022 (26 174 to 30 259)	16.2 (14.9 to 17.8)	1 052 (754 to 1 430)	16.0 (14.5 to 17.7)	30 (29 to 31)	-25.5 (-27.6 to -22.8)	1 136 (1 101 to 1 178)	-30.4 (-32.4 to -27.7)	2 188 (1 894 to 2 550)	-13.8 (-17.4 to -10.2)
South Korea	10 174 (9 481 to 10 972)	-12.3 (-16.1 to -8.5)	24 509 (22 933 to 26 295)	-14.5 (-15.6 to -13.3)	912 (645 to 1 249)	-24.0 (-26.7 to -20.9)	43 (39 to 46)	-48.1 (-52.1 to -44.1)	1 463 (1 357 to 1 569)	-61.1 (-64.3 to -57.8)	2 374 (2 098 to 2 719)	-52.1 (-55.4 to -48.6)
Singapore	10 472 (9 745 to 11 336)	3.9 (1.0 to 7.0)	26 128 (24 475 to 28 102)	6.0 (4.9 to 7.1)	966 (683 to 1 323)	2.9 (0.8 to 4.8)	15 (14 to 16)	-56.8 (-59.7 to -54.0)	611 (571 to 650)	-59.1 (-61.8 to -56.5)	1 577 (1 296 to 1 925)	-35.2 (-40.7 to -30.0)
**High-income North America**	**11 105 (10 424 to 11 773)**	**-19.6 (-22.7 to -16.5)**	**21 960 (20 775 to 23 431)**	**-30.9 (-32.8 to -29.3)**	**813 (589 to 1 097)**	**-29.0 (-30.9 to -27.3)**	**45 (44 to 46)**	**-18.2 (-20.5 to -16.1)**	**1 954 (1 898 to 2 006)**	**-27.0 (-29.1 to -24.9)**	**2 768 (2 535 to 3 060)**	**-27.6 (-29.3 to -25.9)**
Canada	10 100 (9 493 to 10 744)	2.1 (-0.4 to 4.8)	22 701 (21 505 to 24 375)	-0.2 (-1.2 to 0.6)	833 (593 to 1 132)	0.2 (-1.1 to 1.5)	33 (31 to 35)	-29.3 (-33.1 to -25.2)	1 301 (1 225 to 1 375)	-38.3 (-41.9 to -34.4)	2 134 (1 890 to 2 437)	-27.4 (-31.2 to -24.0)
Greenland	11 115 (10 511 to 11 735)	-29.6 (-32.0 to -27.5)	24 586 (23 195 to 26 290)	-34.8 (-35.8 to -33.8)	948 (682 to 1 281)	-39.8 (-41.8 to -38.0)	104 (98 to 112)	-51.2 (-55.0 to -46.6)	4 474 (4 153 to 4 799)	-58.4 (-61.9 to -54.5)	5 422 (5 006 to 5 864)	-56.0 (-59.2 to -52.6)
USA	11 236 (10 542 to 11 935)	-21.0 (-24.3 to -17.7)	21 886 (20 690 to 23 380)	-33.2 (-35.2 to -31.5)	811 (589 to 1 093)	-31.2 (-33.2 to -29.5)	46 (45 to 47)	-17.2 (-19.6 to -14.9)	2 024 (1 963 to 2 081)	-26.0 (-28.3 to -23.8)	2 836 (2 605 to 3 129)	-27.6 (-29.4 to -25.7)
**Southern Latin America**	**9 690 (9 170 to 10 228)**	**12.6 (9.7 to 15.7)**	**23 001 (21 744 to 24 469)**	**6.8 (5.6 to 8.1)**	**841 (601 to 1 141)**	**-5.6 (-9.7 to -1.3)**	**46 (43 to 50)**	**-27.9 (-33.4 to -21.6)**	**2 045 (1 890 to 2 230)**	**-30.2 (-35.8 to -23.8)**	**2 886 (2 584 to 3 227)**	**-24.5 (-29.4 to -19.0)**
Argentina	9 895 (9 292 to 10 512)	16.8 (13.6 to 20.2)	23 727 (22 384 to 25 309)	10.5 (9.1 to 12.0)	881 (628 to 1 194)	-1.5 (-5.8 to 2.8)	47 (43 to 53)	-19.0 (-27.2 to -9.4)	2 140 (1 917 to 2 391)	-19.2 (-27.6 to -9.2)	3 021 (2 676 to 3 424)	-14.7 (-21.4 to -6.9)
Chile	9 005 (8 614 to 9 414)	2.8 (-2.1 to 7.6)	21 057 (19 956 to 22 278)	-2.1 (-4.1 to -0.0)	740 (524 to 1 006)	-16.3 (-20.2 to -12.0)	40 (36 to 45)	-48.8 (-54.4 to -42.4)	1 688 (1 494 to 1 903)	-53.3 (-58.7 to -47.0)	2 428 (2 125 to 2 778)	-46.1 (-51.1 to -40.9)
Uruguay	10 459 (9 811 to 11 113)	10.4 (6.3 to 14.7)	24 317 (23 010 to 26 012)	6.6 (5.3 to 8.2)	877 (624 to 1 192)	-1.6 (-5.2 to 1.8)	59 (53 to 66)	-6.7 (-16.7 to 3.1)	2 553 (2 276 to 2 833)	-12.6 (-22.3 to -2.5)	3 430 (3 064 to 3 855)	-10.0 (-17.7 to -2.3)
**Western Europe**	**9 652 (9 004 to 10 411)**	**-2.1 (-4.3 to 0.1)**	**23 087 (21 625 to 24 688)**	**-3.0 (-3.7 to -2.3)**	**857 (609 to 1 170)**	**-2.1 (-2.8 to -1.4)**	**25 (25 to 26)**	**-47.8 (-49.4 to -46.0)**	**890 (863 to 918)**	**-55.3 (-56.7 to -53.8)**	**1 747 (1 500 to 2 055)**	**-39.1 (-42.8 to -35.3)**
Andorra	10 160 (9 433 to 10 988)	2.8 (-0.0 to 5.7)	24 421 (22 872 to 26 130)	-0.3 (-1.3 to 0.8)	911 (650 to 1 243)	2.5 (1.1 to 3.9)	23 (20 to 26)	-35.2 (-46.5 to -21.8)	854 (738 to 980)	-43.5 (-54.6 to -30.1)	1 765 (1 473 to 2 109)	-26.4 (-35.3 to -16.5)
Austria	10 021 (9 338 to 10 801)	-10.8 (-14.5 to -7.3)	23 861 (22 338 to 25 562)	-8.8 (-9.9 to -7.8)	892 (633 to 1 220)	-9.0 (-10.3 to -7.7)	29 (27 to 31)	-50.8 (-53.9 to -47.8)	1 009 (946 to 1 079)	-57.8 (-60.5 to -54.9)	1 901 (1 621 to 2 219)	-43.6 (-47.5 to -39.8)
Belgium	11 238 (10 504 to 12 106)	9.4 (6.3 to 12.8)	25 781 (24 082 to 27 634)	7.4 (6.3 to 8.6)	968 (688 to 1 322)	6.6 (4.9 to 8.3)	38 (36 to 40)	-33.9 (-37.6 to -30.0)	1 348 (1 267 to 1 431)	-44.6 (-48.2 to -41.2)	2 316 (2 028 to 2 695)	-30.7 (-34.7 to -26.3)
Cyprus	9 813 (9 128 to 10 596)	-2.3 (-5.0 to 0.7)	24 023 (22 510 to 25 656)	-3.3 (-4.4 to -2.2)	893 (633 to 1 218)	-8.8 (-10.9 to -6.7)	28 (25 to 31)	-48.6 (-53.9 to -42.9)	1 061 (963 to 1 170)	-49.6 (-55.1 to -44.3)	1 954 (1 673 to 2 298)	-36.7 (-41.7 to -32.0)
Denmark	9 845 (9 128 to 10 656)	-5.1 (-8.4 to -1.9)	23 394 (21 914 to 25 091)	0.2 (-1.1 to 1.4)	866 (617 to 1 183)	-1.8 (-3.5 to -0.2)	25 (23 to 26)	-60.3 (-62.7 to -57.6)	799 (745 to 855)	-64.2 (-66.7 to -61.6)	1 665 (1 404 to 1 995)	-46.5 (-51.2 to -42.1)
Finland	11 613 (10 821 to 12 539)	1.4 (-1.9 to 5.0)	27 049 (25 354 to 28 958)	3.7 (2.8 to 4.8)	1 014 (720 to 1 384)	4.4 (2.9 to 5.9)	35 (33 to 38)	-52.4 (-55.3 to -49.0)	1 282 (1 203 to 1 380)	-58.8 (-61.5 to -55.7)	2 296 (1 993 to 2 681)	-43.8 (-47.9 to -39.3)
France	10 387 (9 663 to 11 197)	-6.6 (-9.5 to -3.8)	24 567 (23 066 to 26 242)	-6.2 (-7.5 to -5.0)	919 (654 to 1 258)	-5.1 (-6.5 to -3.8)	35 (33 to 37)	-51.9 (-54.8 to -48.7)	1 202 (1 128 to 1 277)	-55.7 (-58.6 to -52.8)	2 121 (1 842 to 2 452)	-42.4 (-46.5 to -38.6)
Germany	9 866 (9 186 to 10 660)	2.7 (-0.5 to 6.0)	23 243 (21 720 to 24 940)	2.0 (0.9 to 3.1)	869 (615 to 1 192)	2.4 (0.9 to 3.8)	26 (24 to 29)	-43.1 (-48.3 to -37.2)	919 (826 to 1 022)	-52.4 (-57.1 to -47.1)	1 787 (1 521 to 2 109)	-35.6 (-40.7 to -30.5)
Greece	9 505 (8 818 to 10 265)	-1.7 (-4.4 to 1.0)	23 217 (21 781 to 24 828)	-2.2 (-3.2 to -1.4)	861 (611 to 1 177)	-2.2 (-3.5 to -0.8)	24 (23 to 25)	-39.7 (-43.1 to -36.2)	998 (938 to 1 062)	-43.2 (-46.9 to -39.5)	1 859 (1 594 to 2 175)	-29.6 (-33.7 to -25.4)
Iceland	9 856 (9 166 to 10 640)	2.7 (-0.0 to 5.6)	23 643 (22 128 to 25 380)	3.4 (2.4 to 4.3)	881 (627 to 1 204)	3.0 (1.6 to 4.5)	26 (25 to 27)	-40.9 (-44.0 to -37.3)	949 (906 to 997)	-48.3 (-51.2 to -45.1)	1 830 (1 565 to 2 160)	-32.0 (-36.5 to -27.5)
Ireland	9 622 (8 933 to 10 394)	3.5 (0.8 to 6.4)	23 368 (21 899 to 25 065)	3.6 (2.5 to 4.7)	860 (610 to 1 169)	4.6 (3.1 to 6.4)	21 (19 to 22)	-48.6 (-52.3 to -44.8)	814 (754 to 876)	-52.6 (-56.1 to -49.0)	1 674 (1 417 to 1 979)	-34.1 (-39.1 to -29.6)
Israel	9 398 (8 717 to 10 200)	-1.3 (-4.3 to 1.5)	23 521 (21 867 to 25 333)	8.4 (6.0 to 11.4)	882 (630 to 1 194)	6.9 (4.2 to 11.5)	23 (21 to 24)	-44.7 (-48.3 to -37.4)	831 (782 to 887)	-49.3 (-52.7 to -44.3)	1 713 (1 454 to 2 022)	-30.5 (-35.2 to -25.3)
Italy	8 493 (7 926 to 9 144)	-11.8 (-14.7 to -8.9)	20 984 (19 754 to 22 312)	-15.3 (-16.3 to -14.3)	772 (549 to 1 052)	-11.7 (-13.0 to -10.3)	20 (19 to 21)	-52.5 (-55.5 to -49.6)	686 (640 to 733)	-58.3 (-61.0 to -55.4)	1 458 (1 237 to 1 739)	-42.1 (-46.1 to -38.1)
Luxembourg	10 443 (9 744 to 11 262)	-13.8 (-17.0 to -10.6)	25 143 (23 615 to 26 823)	-10.2 (-11.5 to -9.2)	931 (662 to 1 264)	-10.3 (-11.6 to -8.7)	28 (26 to 31)	-51.4 (-56.1 to -46.6)	938 (847 to 1 034)	-62.0 (-65.8 to -57.9)	1 869 (1 587 to 2 203)	-46.6 (-51.0 to -42.2)
Malta	10 281 (9 557 to 11 070)	4.1 (0.9 to 7.4)	24 671 (23 055 to 26 546)	5.8 (4.4 to 6.9)	919 (654 to 1 253)	8.4 (6.7 to 10.3)	21 (20 to 22)	-29.1 (-33.1 to -25.0)	739 (701 to 780)	-32.5 (-36.7 to -27.5)	1 657 (1 384 to 1 993)	-14.6 (-19.3 to -10.4)
Netherlands	8 514 (8 048 to 9 047)	3.3 (-0.9 to 7.6)	19 490 (18 312 to 20 776)	0.7 (-0.6 to 2.4)	727 (516 to 991)	1.2 (-0.4 to 3.0)	26 (25 to 28)	-25.8 (-30.0 to -21.3)	803 (759 to 855)	-42.1 (-45.3 to -38.1)	1 530 (1 306 to 1 796)	-27.3 (-31.4 to -23.5)
Norway	13 020 (12 190 to 13 912)	13.0 (10.6 to 15.3)	25 768 (24 082 to 27 737)	-1.3 (-2.3 to -0.4)	1 031 (746 to 1 383)	2.0 (0.8 to 3.3)	29 (28 to 29)	-46.7 (-48.5 to -44.7)	970 (942 to 1 007)	-55.9 (-57.4 to -54.1)	2 001 (1 720 to 2 353)	-37.6 (-41.6 to -33.7)
Portugal	8 231 (7 680 to 8 845)	-20.5 (-23.6 to -17.2)	19 975 (18 800 to 21 266)	-24.2 (-25.5 to -22.8)	729 (517 to 998)	-27.8 (-29.7 to -26.0)	25 (24 to 27)	-64.0 (-66.4 to -61.6)	930 (863 to 997)	-71.7 (-73.8 to -69.5)	1 659 (1 436 to 1 910)	-61.4 (-64.6 to -58.4)
Spain	9 205 (8 561 to 9 963)	-4.2 (-7.1 to -1.2)	22 635 (21 241 to 24 209)	-5.1 (-6.1 to -4.1)	836 (595 to 1 140)	-3.6 (-5.0 to -2.1)	19 (18 to 20)	-57.5 (-60.0 to -54.7)	668 (625 to 709)	-67.2 (-69.3 to -65.0)	1 504 (1 253 to 1 806)	-48.2 (-52.8 to -43.8)
Sweden	10 501 (9 741 to 11 370)	9.5 (7.1 to 11.9)	25 234 (23 552 to 27 248)	6.5 (5.5 to 7.5)	958 (693 to 1 292)	7.3 (5.7 to 8.9)	27 (26 to 29)	-38.0 (-41.1 to -34.9)	950 (899 to 1 005)	-47.0 (-50.0 to -43.9)	1 907 (1 633 to 2 237)	-28.9 (-33.3 to -24.7)
Switzerland	10 336 (9 749 to 11 004)	-22.0 (-25.2 to -18.3)	23 574 (22 183 to 25 259)	-19.4 (-20.9 to -17.8)	879 (629 to 1 199)	-21.1 (-22.5 to -19.7)	25 (24 to 27)	-58.2 (-61.0 to -55.2)	804 (751 to 860)	-66.4 (-68.7 to -63.8)	1 683 (1 421 to 2 015)	-52.0 (-55.7 to -48.5)
United Kingdom	9 453 (8 757 to 10 256)	8.2 (6.0 to 10.4)	23 459 (21 938 to 25 167)	9.5 (8.5 to 10.7)	850 (603 to 1 160)	9.8 (8.9 to 10.7)	21 (21 to 21)	-33.0 (-34.2 to -31.9)	774 (762 to 787)	-44.3 (-45.4 to -43.3)	1 623 (1 377 to 1 938)	-25.0 (-29.1 to -20.8)
**Latin America and Caribbean**	**4 602 (4 348 to 4 889)**	**7.2 (4.9 to 9.6)**	**12 674 (12 018 to 13 427)**	**6.3 (4.5 to 8.1)**	**485 (359 to 638)**	**1.1 (-2.4 to 4.3)**	**72 (71 to 74)**	**-26.9 (-28.4 to -25.3)**	**3 477 (3 410 to 3 550)**	**-28.4 (-30.1 to -26.8)**	**3 962 (3 814 to 4 121)**	**-25.8 (-27.6 to -23.9)**
**Andean Latin America**	**4 407 (4 171 to 4 669)**	**-6.4 (-9.6 to -3.4)**	**11 823 (11 233 to 12 496)**	**1.3 (-0.2 to 2.9)**	**452 (332 to 597)**	**-17.9 (-23.5 to -12.0)**	**53 (48 to 57)**	**-44.5 (-49.7 to -39.1)**	**2 446 (2 219 to 2 677)**	**-52.5 (-57.7 to -47.0)**	**2 898 (2 638 to 3 150)**	**-49.1 (-54.1 to -43.9)**
Bolivia	4 188 (3 956 to 4 440)	3.0 (-0.1 to 5.9)	11 048 (10 510 to 11 663)	-0.4 (-1.9 to 1.1)	444 (326 to 584)	-14.5 (-18.8 to -9.8)	60 (47 to 72)	-51.1 (-62.7 to -39.4)	2 580 (1 862 to 3 073)	-58.7 (-70.9 to -47.2)	3 024 (2 301 to 3 564)	-55.3 (-66.8 to -44.2)
Ecuador	4 753 (4 496 to 5 027)	13.8 (10.2 to 17.8)	12 604 (11 963 to 13 316)	4.9 (3.2 to 6.6)	477 (353 to 628)	-16.0 (-22.0 to -9.8)	73 (66 to 80)	-14.1 (-22.0 to -4.9)	3 393 (3 070 to 3 759)	-13.5 (-21.9 to -3.7)	3 869 (3 532 to 4 258)	-13.8 (-21.3 to -5.3)
Peru	4 321 (4 082 to 4 598)	-15.9 (-19.7 to -12.3)	11 666 (11 019 to 12 434)	-0.3 (-2.2 to 1.9)	442 (325 to 581)	-19.9 (-26.3 to -13.1)	41 (35 to 47)	-55.5 (-62.2 to -48.0)	1 913 (1 627 to 2 215)	-64.3 (-70.3 to -57.7)	2 354 (2 038 to 2 689)	-60.1 (-65.7 to -53.8)
**Caribbean**	**5 040 (4 778 to 5 318)**	**30.2 (27.6 to 33.0)**	**16 935 (15 788 to 18 177)**	**63.5 (54.6 to 74.4)**	**706 (526 to 915)**	**69.3 (52.5 to 91.9)**	**79 (74 to 85)**	**-13.4 (-19.5 to -6.2)**	**3 647 (3 373 to 3 955)**	**-18.7 (-25.8 to -9.2)**	**4 353 (4 013 to 4 707)**	**-11.2 (-18.3 to -2.2)**
Antigua and Barbuda	4 924 (4 640 to 5 219)	25.0 (21.3 to 28.7)	12 370 (11 789 to 13 015)	17.0 (15.0 to 18.9)	406 (294 to 545)	6.0 (1.7 to 10.5)	37 (34 to 40)	-20.2 (-27.1 to -13.2)	1 698 (1 556 to 1 847)	-15.8 (-24.0 to -7.3)	2 104 (1 929 to 2 315)	-12.3 (-19.3 to -5.1)
The Bahamas	4 477 (4 232 to 4 744)	21.7 (18.9 to 24.6)	11 883 (11 328 to 12 507)	5.0 (2.6 to 7.5)	407 (296 to 543)	4.1 (0.2 to 7.6)	71 (65 to 77)	-6.0 (-14.2 to 3.0)	3 382 (3 085 to 3 681)	-2.5 (-11.7 to 7.2)	3 789 (3 469 to 4 130)	-1.9 (-10.2 to 7.0)
Barbados	4 376 (4 122 to 4 665)	28.0 (25.5 to 30.9)	11 285 (10 750 to 11 901)	20.1 (18.1 to 22.2)	376 (273 to 504)	14.1 (9.5 to 18.5)	37 (34 to 41)	-19.7 (-26.4 to -12.6)	1 649 (1 512 to 1 795)	-20.9 (-28.0 to -13.4)	2 025 (1 851 to 2 220)	-16.1 (-22.3 to -9.6)
Belize	4 802 (4 554 to 5 076)	32.5 (29.5 to 35.6)	13 213 (12 598 to 13 913)	32.1 (29.4 to 35.1)	494 (367 to 652)	25.3 (20.8 to 29.9)	83 (79 to 87)	7.6 (-0.2 to 15.4)	3 910 (3 710 to 4 119)	6.5 (-1.6 to 15.9)	4 404 (4 159 to 4 646)	8.3 (0.8 to 16.7)
Bermuda	4 739 (4 459 to 5 042)	26.2 (23.2 to 29.3)	13 790 (13 100 to 14 577)	6.3 (3.6 to 8.9)	428 (310 to 568)	6.7 (0.9 to 11.6)	24 (22 to 27)	-50.5 (-55.1 to -45.5)	1 004 (899 to 1 116)	-54.4 (-59.6 to -48.5)	1 432 (1 267 to 1 607)	-45.0 (-50.2 to -39.5)
Cuba	5 226 (4 943 to 5 548)	25.2 (21.7 to 29.0)	12 091 (11 509 to 12 787)	18.5 (16.6 to 20.2)	409 (292 to 553)	8.5 (4.5 to 12.7)	48 (43 to 53)	-39.6 (-45.3 to -32.9)	1 641 (1 473 to 1 846)	-50.3 (-55.3 to -44.2)	2 051 (1 844 to 2 267)	-44.2 (-49.1 to -38.7)
Dominica	4 294 (4 054 to 4 561)	33.0 (30.2 to 35.9)	11 861 (11 272 to 12 493)	23.4 (20.7 to 26.1)	421 (308 to 558)	20.4 (15.5 to 25.1)	56 (52 to 60)	-1.7 (-9.2 to 6.6)	2 732 (2 516 to 2 987)	4.1 (-5.1 to 14.5)	3 152 (2 913 to 3 432)	6.0 (-2.3 to 15.1)
Dominican Republic	5 011 (4 748 to 5 296)	41.8 (38.9 to 45.2)	12 499 (11 930 to 13 137)	32.0 (29.2 to 34.9)	464 (337 to 622)	8.9 (1.5 to 17.1)	80 (69 to 92)	19.8 (-2.8 to 42.5)	3 744 (3 221 to 4 283)	10.1 (-10.5 to 30.9)	4 208 (3 681 to 4 764)	10.0 (-8.7 to 28.2)
Grenada	4 551 (4 301 to 4 819)	23.9 (20.6 to 27.3)	12 159 (11 574 to 12 827)	11.8 (9.6 to 14.2)	439 (321 to 582)	7.7 (3.2 to 12.0)	45 (42 to 48)	-32.5 (-37.0 to -27.3)	1 902 (1 774 to 2 044)	-35.0 (-40.2 to -29.0)	2 341 (2 160 to 2 535)	-29.8 (-35.0 to -24.3)
Guyana	4 615 (4 374 to 4 863)	27.0 (23.9 to 30.3)	11 844 (11 306 to 12 511)	14.7 (12.4 to 17.2)	470 (345 to 620)	11.3 (6.8 to 15.9)	95 (84 to 107)	-2.2 (-13.5 to 10.3)	4 246 (3 738 to 4 780)	-1.8 (-13.7 to 11.4)	4 716 (4 231 to 5 247)	-0.7 (-11.5 to 11.6)
Haiti	3 829 (3 619 to 4 057)	6.5 (3.9 to 9.1)	29 355 (25 657 to 33 618)	147.8 (118.7 to 184.3)	1 433 (1 052 to 1 894)	164.0 (116.7 to 225.8)	108 (90 to 132)	-38.4 (-48.9 to -24.6)	5 052 (4 206 to 6 110)	-45.1 (-55.0 to -30.4)	6 485 (5 560 to 7 616)	-33.5 (-44.5 to -18.2)
Jamaica	4 950 (4 670 to 5 245)	32.0 (28.5 to 35.7)	12 627 (12 032 to 13 320)	29.5 (27.4 to 31.6)	449 (324 to 601)	21.9 (16.7 to 26.7)	56 (47 to 65)	89.2 (58.4 to 123.0)	2 616 (2 209 to 3 055)	94.5 (62.2 to 130.8)	3 065 (2 630 to 3 529)	78.9 (53.7 to 107.7)
Puerto Rico	8 811 (8 128 to 9 644)	120.3 (104.0 to 139.4)	13 127 (12 509 to 13 864)	27.7 (25.6 to 30.0)	478 (341 to 640)	31.4 (27.3 to 35.9)	105 (102 to 109)	50.1 (44.8 to 55.7)	5 175 (5 011 to 5 336)	57.8 (52.0 to 63.9)	5 653 (5 442 to 5 868)	55.1 (49.9 to 60.6)
Saint Lucia	4 387 (4 155 to 4 655)	24.7 (21.8 to 27.6)	11 760 (11 207 to 12 391)	11.3 (9.2 to 13.6)	410 (299 to 546)	4.7 (-0.2 to 9.4)	57 (53 to 61)	-20.3 (-26.0 to -14.1)	2 591 (2 409 to 2 782)	-17.9 (-24.1 to -10.9)	3 001 (2 798 to 3 222)	-15.4 (-21.2 to -8.9)
Saint Vincent and the Grenadines	4 655 (4 409 to 4 918)	36.4 (33.2 to 39.7)	12 257 (11 675 to 12 924)	30.2 (27.5 to 33.0)	455 (334 to 601)	26.9 (22.4 to 31.0)	63 (59 to 67)	4.3 (-3.1 to 12.0)	2 917 (2 731 to 3 107)	7.0 (-1.5 to 15.7)	3 372 (3 156 to 3 618)	9.3 (1.9 to 17.0)
Suriname	4 585 (4 345 to 4 852)	26.2 (23.3 to 29.3)	12 177 (11 616 to 12 844)	15.4 (12.8 to 17.6)	468 (344 to 617)	2.6 (-2.0 to 7.5)	79 (71 to 88)	-17.4 (-27.2 to -6.9)	3 486 (3 107 to 3 886)	-22.1 (-31.3 to -11.9)	3 955 (3 581 to 4 377)	-19.8 (-28.4 to -10.3)

Location	Incidence (95% UI)		Prevalence (95% UI)		Years lived with disability (95% UI)		Deaths (95% UI)		Years of life lost (95% UI)		Disability adjusted life years (95% UI)	
	2017 age-standardised rates per 100 000	Percentage change in age-standardised rates between 1990 and 2017	2017 age-standardised rates per 100 000	Percentage change in age-standardised rates between 1990 and 2017	2017 age-standardised rates per 100 000	Percentage change in age-standardised rates between 1990 and 2017	2017 age-standardised rates per 100 000	Percentage change in age-standardised rates between 1990 and 2017	2017 age-standardised rates per 100 000	Percentage change in age-standardised rates between 1990 and 2017	2017 age-standardised rates per 100 000	Percentage change in age-standardised rates between 1990 and 2017
Trinidad and Tobago	4 563 (4 300 to 4 854)	14.7 (11.1 to 18.6)	11 563 (11 032 to 12 187)	20.6 (18.3 to 23.4)	421 (306 to 561)	11.3 (6.4 to 16.1)	63 (51 to 76)	-7.0 (-23.8 to 12.2)	3 073 (2 504 to 3 725)	-3.6 (-21.3 to 16.4)	3 494 (2 904 to 4 148)	-2.0 (-18.0 to 15.7)
Virgin Islands	5 275 (5 000 to 5 568)	35.7 (32.3 to 39.2)	12 377 (11 822 to 13 025)	21.5 (19.5 to 23.3)	413 (295 to 557)	7.7 (2.5 to 13.6)	69 (63 to 77)	-4.6 (-14.8 to 7.4)	2 874 (2 630 to 3 205)	-9.4 (-18.5 to 2.6)	3 287 (3 018 to 3 667)	-7.6 (-15.9 to 3.2)
**Central Latin America**	**4 256 (3 996 to 4 543)**	**-3.1 (-5.1 to -1.0)**	**11 668 (11 066 to 12 405)**	**-10.0 (-11.3 to -8.6)**	**427 (317 to 558)**	**-17.4 (-21.5 to -13.9)**	**74 (71 to 77)**	**-28.5 (-31.2 to -25.8)**	**3 629 (3 491 to 3 769)**	**-27.1 (-30.0 to -24.3)**	**4 056 (3 866 to 4 248)**	**-26.2 (-28.9 to -23.6)**
Colombia	3 674 (3 468 to 3 896)	-18.6 (-21.6 to -15.4)	10 366 (9 746 to 11 074)	-15.9 (-18.0 to -13.6)	372 (277 to 486)	-29.7 (-34.0 to -25.1)	61 (53 to 68)	-55.8 (-60.9 to -50.2)	3 114 (2 753 to 3 501)	-56.0 (-61.1 to -50.4)	3 486 (3 101 to 3 876)	-54.1 (-59.2 to -49.0)
Costa Rica	4 086 (3 836 to 4 331)	17.5 (14.1 to 21.3)	9 710 (9 223 to 10 251)	13.9 (12.1 to 15.8)	337 (242 to 451)	4.5 (-0.1 to 9.1)	51 (47 to 54)	-14.5 (-20.4 to -8.1)	2 130 (1 987 to 2 280)	-8.5 (-14.9 to -1.4)	2 468 (2 299 to 2 660)	-6.9 (-12.6 to -0.6)
El Salvador	3 921 (3 696 to 4 167)	-27.2 (-32.8 to -22.1)	14 810 (12 861 to 17 465)	-27.0 (-31.4 to -21.7)	560 (415 to 726)	-48.7 (-55.7 to -41.1)	106 (87 to 125)	-31.7 (-43.7 to -19.0)	4 924 (4 032 to 5 883)	-35.7 (-47.7 to -23.5)	5 484 (4 572 to 6 440)	-37.4 (-47.9 to -26.3)
Guatemala	4 001 (3 769 to 4 238)	-11.8 (-16.1 to -7.8)	11 861 (10 975 to 13 046)	-18.2 (-22.7 to -13.5)	477 (355 to 616)	-35.7 (-43.4 to -28.2)	92 (82 to 102)	-27.9 (-35.3 to -19.3)	4 351 (3 894 to 4 872)	-29.5 (-36.7 to -20.8)	4 828 (4 351 to 5 366)	-30.2 (-37.0 to -22.1)
Honduras	3 624 (3 414 to 3 845)	10.9 (7.8 to 13.9)	11 661 (10 846 to 12 651)	30.0 (22.5 to 39.3)	478 (361 to 612)	14.8 (7.3 to 24.1)	84 (68 to 103)	-29.0 (-44.3 to -10.2)	3 864 (3 095 to 4 647)	-36.1 (-50.5 to -19.2)	4 342 (3 549 to 5 175)	-32.8 (-46.3 to -16.9)
Mexico	4 576 (4 234 to 4 961)	1.4 (-1.2 to 4.1)	11 926 (11 275 to 12 691)	-10.7 (-12.2 to -9.1)	437 (321 to 579)	-8.7 (-11.2 to -6.6)	74 (73 to 75)	-19.4 (-21.1 to -18.1)	3 535 (3 486 to 3 588)	-16.1 (-17.8 to -14.3)	3 971 (3 842 to 4 120)	-15.4 (-16.9 to -13.7)
Nicaragua	3 425 (3 216 to 3 650)	3.4 (0.1 to 6.9)	13 965 (12 037 to 16 625)	-28.0 (-32.2 to -23.0)	537 (395 to 701)	-44.2 (-51.1 to -36.9)	38 (34 to 45)	-46.7 (-53.7 to -37.4)	1 701 (1 496 to 1 993)	-52.9 (-59.0 to -44.7)	2 238 (1 986 to 2 550)	-51.1 (-56.4 to -44.4)
Panama	3 799 (3 577 to 4 036)	14.2 (10.7 to 17.7)	9 792 (9 309 to 10 353)	12.0 (10.2 to 13.9)	343 (250 to 455)	-3.7 (-9.1 to 2.0)	47 (44 to 51)	-25.7 (-31.4 to -19.8)	2 319 (2 119 to 2 519)	-23.3 (-29.8 to -16.6)	2 663 (2 451 to 2 883)	-21.2 (-27.2 to -15.1)
Venezuela	4 665 (4 395 to 4 948)	14.1 (10.2 to 18.5)	12 738 (12 032 to 13 509)	17.1 (13.4 to 21.8)	440 (323 to 581)	2.9 (-2.3 to 9.0)	95 (81 to 111)	12.2 (-5.0 to 31.3)	5 025 (4 242 to 5 890)	20.7 (1.8 to 41.1)	5 466 (4 708 to 6 319)	19.0 (2.1 to 37.3)
**Tropical Latin America**	**4 942 (4 658 to 5 260)**	**17.4 (12.8 to 21.8)**	**13 110 (12 352 to 13 946)**	**15.4 (12.8 to 18.4)**	**511 (374 to 674)**	**15.7 (13.0 to 18.4)**	**74 (73 to 75)**	**-23.6 (-25.2 to -21.8)**	**3 539 (3 486 to 3 592)**	**-25.1 (-27.0 to -22.8)**	**4 049 (3 899 to 4 214)**	**-21.7 (-23.7 to -19.3)**
Brazil	4 948 (4 665 to 5 266)	17.4 (12.7 to 21.8)	13 152 (12 387 to 13 993)	15.5 (12.9 to 18.6)	512 (375 to 675)	16.1 (13.4 to 18.8)	74 (73 to 75)	-24.0 (-25.6 to -22.2)	3 573 (3 516 to 3 625)	-25.4 (-27.1 to -23.0)	4 085 (3 936 to 4 247)	-21.9 (-23.9 to -19.5)
Paraguay	4 711 (4 417 to 5 030)	17.6 (14.7 to 20.5)	11 417 (10 816 to 12 111)	7.7 (5.8 to 9.7)	460 (334 to 612)	1.2 (-3.2 to 5.5)	58 (48 to 69)	12.5 (-7.9 to 37.3)	2 572 (2 138 to 3 096)	5.8 (-14.2 to 29.7)	3 032 (2 592 to 3 579)	5.1 (-11.8 to 25.2)
**North Africa and Middle East**	**7 489 (7 075 to 7 917)**	**8.3 (5.5 to 12.4)**	**19 309 (17 984 to 20 921)**	**-2.5 (-3.4 to -1.4)**	**771 (573 to 1 008)**	**-19.3 (-23.2 to -14.6)**	**65 (62 to 69)**	**-32.6 (-36.4 to -25.8)**	**3 285 (3 141 to 3 462)**	**-34.5 (-38.4 to -26.4)**	**4 056 (3 796 to 4 363)**	**-32.1 (-35.7 to -25.3)**
**North Africa and Middle East**	**7 489 (7 075 to 7 917)**	**8.3 (5.5 to 12.4)**	**19 309 (17 984 to 20 921)**	**-2.5 (-3.4 to -1.4)**	**771 (573 to 1 008)**	**-19.3 (-23.2 to -14.6)**	**65 (62 to 69)**	**-32.6 (-36.4 to -25.8)**	**3 285 (3 141 to 3 462)**	**-34.5 (-38.4 to -26.4)**	**4 056 (3 796 to 4 363)**	**-32.1 (-35.7 to -25.3)**
Afghanistan	5 993 (5 697 to 6 310)	-16.4 (-19.1 to -14.0)	37 463 (31 795 to 44 643)	-24.1 (-26.5 to -21.6)	1 995 (1 442 to 2 642)	-42.3 (-48.5 to -34.9)	103 (86 to 122)	-35.9 (-51.9 to 37.6)	4 913 (4 067 to 5 876)	-40.9 (-56.9 to 44.4)	6 909 (5 920 to 8 048)	-41.3 (-53.2 to 3.5)
Algeria	5 787 (5 477 to 6 129)	-0.6 (-3.2 to 1.9)	16 214 (15 450 to 17 155)	-1.1 (-3.0 to 0.9)	624 (455 to 820)	-18.9 (-23.4 to -13.7)	36 (31 to 47)	-44.2 (-49.2 to -38.7)	1 731 (1 483 to 2 172)	-48.2 (-53.4 to -42.2)	2 355 (2 039 to 2 823)	-42.7 (-47.4 to -37.7)
Bahrain	6 274 (5 889 to 6 677)	10.6 (7.9 to 13.4)	16 582 (15 795 to 17 598)	5.5 (3.7 to 7.2)	575 (411 to 773)	-13.8 (-19.1 to -8.3)	23 (21 to 26)	-50.6 (-56.2 to -44.3)	1 014 (922 to 1 126)	-50.4 (-55.5 to -44.4)	1 589 (1 406 to 1 824)	-41.4 (-46.2 to -35.9)
Egypt	5 635 (5 331 to 5 977)	16.1 (13.2 to 19.2)	14 415 (13 797 to 15 269)	4.9 (3.3 to 6.4)	572 (416 to 763)	-17.7 (-23.2 to -11.7)	54 (44 to 64)	-32.0 (-43.2 to -21.1)	2 473 (2 109 to 2 828)	-39.9 (-47.7 to -29.0)	3 045 (2 660 to 3 451)	-36.7 (-43.5 to -27.3)
Iran	6 364 (6 022 to 6 741)	-38.4 (-40.3 to -36.5)	19 854 (18 518 to 21 427)	-15.7 (-18.7 to -13.1)	732 (543 to 962)	-36.4 (-42.1 to -30.7)	53 (52 to 54)	-70.1 (-71.9 to -68.7)	2 512 (2 464 to 2 611)	-72.5 (-73.4 to -71.3)	3 244 (3 051 to 3 499)	-68.4 (-69.9 to -66.8)
Iraq	13 245 (12 199 to 14 862)	36.4 (27.7 to 48.3)	34 339 (29 480 to 40 416)	-24.2 (-26.1 to -22.1)	1 552 (1 125 to 2 064)	-30.1 (-34.0 to -26.0)	115 (112 to 118)	14.0 (0.4 to 36.2)	6 311 (6 151 to 6 472)	22.7 (8.8 to 48.3)	7 863 (7 434 to 8 416)	6.8 (-2.8 to 21.6)
Jordan	5 436 (5 115 to 5 785)	-2.9 (-6.2 to 0.4)	14 165 (13 521 to 15 010)	-9.2 (-10.3 to -7.9)	494 (352 to 662)	-25.4 (-29.5 to -20.9)	29 (26 to 32)	-51.5 (-57.9 to -43.4)	1 387 (1 247 to 1 542)	-54.3 (-60.8 to -46.2)	1 881 (1 665 to 2 112)	-49.1 (-54.9 to -41.8)
Kuwait	6 607 (6 220 to 7 009)	-48.6 (-53.5 to -44.4)	17 816 (16 980 to 18 930)	-2.2 (-3.7 to -0.7)	626 (449 to 843)	-16.7 (-20.7 to -13.0)	25 (23 to 26)	-78.7 (-80.1 to -77.2)	1 073 (1 004 to 1 146)	-82.5 (-83.6 to -81.3)	1 700 (1 517 to 1 939)	-75.3 (-77.4 to -72.7)
Lebanon	6 501 (6 155 to 6 905)	-10.7 (-14.9 to -7.0)	22 721 (20 322 to 25 758)	-19.6 (-24.2 to -14.6)	803 (585 to 1 061)	-39.5 (-47.3 to -31.6)	33 (29 to 37)	-67.0 (-71.8 to -62.0)	1 447 (1 282 to 1 603)	-71.2 (-75.1 to -67.6)	2 249 (1 985 to 2 553)	-64.6 (-68.2 to -61.2)
Libya	8 195 (7 712 to 8 746)	33.5 (28.0 to 41.7)	22 123 (19 882 to 25 019)	31.3 (20.2 to 46.0)	855 (629 to 1 127)	16.3 (2.8 to 40.1)	85 (71 to 99)	24.8 (3.1 to 48.4)	4 132 (3 534 to 4 734)	28.3 (7.4 to 51.4)	4 987 (4 343 to 5 644)	26.1 (8.9 to 43.7)
Morocco	5 391 (5 107 to 5 708)	6.3 (3.7 to 9.0)	14 407 (13 775 to 15 236)	-0.7 (-1.9 to 0.5)	566 (415 to 753)	-17.3 (-21.8 to -12.4)	46 (37 to 60)	-36.3 (-47.8 to -24.0)	2 144 (1 729 to 2 782)	-42.8 (-53.5 to -31.3)	2 710 (2 246 to 3 374)	-38.9 (-48.1 to -29.1)
Palestine	5 920 (5 599 to 6 270)	-19.9 (-24.0 to -16.4)	29 839 (25 182 to 36 034)	20.5 (13.6 to 29.8)	1 254 (916 to 1 672)	2.5 (-3.6 to 8.7)	31 (27 to 34)	-55.3 (-61.8 to -49.6)	1 455 (1 242 to 1 607)	-60.6 (-66.5 to -54.8)	2 709 (2 321 to 3 152)	-44.9 (-51.0 to -38.9)
Oman	6 585 (6 218 to 6 988)	-1.5 (-4.4 to 1.6)	17 149 (16 432 to 18 131)	-2.7 (-4.2 to -1.4)	611 (431 to 830)	-20.3 (-25.2 to -15.3)	61 (50 to 72)	-50.4 (-60.5 to -37.1)	2 601 (2 165 to 3 065)	-54.3 (-63.5 to -41.9)	3 212 (2 733 to 3 736)	-50.3 (-58.8 to -38.8)
Qatar	7 312 (6 916 to 7 741)	-4.7 (-7.5 to -1.8)	18 982 (18 100 to 20 202)	-4.2 (-5.2 to -3.0)	670 (475 to 909)	-16.9 (-20.5 to -13.0)	43 (35 to 51)	-37.4 (-50.1 to -20.5)	1 785 (1 469 to 2 136)	-42.1 (-53.9 to -26.8)	2 454 (2 056 to 2 883)	-36.9 (-46.9 to -24.7)
Saudi Arabia	8 105 (7 687 to 8 560)	3.5 (0.5 to 6.9)	18 688 (17 835 to 19 830)	-8.0 (-8.9 to -7.1)	675 (481 to 915)	-24.9 (-29.6 to -20.0)	72 (61 to 83)	-30.3 (-46.7 to -12.7)	2 989 (2 516 to 3 461)	-35.4 (-49.0 to -19.8)	3 664 (3 136 to 4 208)	-33.7 (-45.5 to -20.8)
Sudan	5 530 (5 235 to 5 820)	-24.3 (-27.2 to -21.2)	19 593 (17 794 to 21 908)	6.9 (2.5 to 12.0)	744 (556 to 966)	-3.6 (-7.0 to 1.2)	62 (49 to 81)	-56.0 (-63.9 to -42.9)	3 105 (2 514 to 3 892)	-60.5 (-68.6 to -46.1)	3 849 (3 247 to 4 653)	-55.4 (-63.7 to -40.7)
Syria	26 544 (24 052 to 30 257)	451.0 (399.1 to 524.5)	32 417 (27 126 to 39 570)	124.2 (93.8 to 162.4)	1 426 (1 029 to 1 949)	122.6 (87.8 to 182.6)	271 (267 to 276)	487.5 (421.2 to 563.2)	14 915 (14 744 to 15 112)	602.2 (530.0 to 696.4)	16 341 (15 893 to 16 858)	491.0 (437.0 to 556.7)
Tunisia	6 262 (5 922 to 6 652)	-3.2 (-6.1 to -0.2)	15 965 (15 232 to 17 022)	-6.9 (-8.3 to -5.6)	561 (402 to 755)	-22.0 (-26.9 to -16.8)	48 (40 to 59)	-39.4 (-51.5 to -25.1)	1 983 (1 608 to 2 380)	-47.3 (-57.3 to -36.0)	2 544 (2 121 to 2 989)	-43.2 (-51.9 to -33.6)
Turkey	6 180 (5 853 to 6 539)	-3.3 (-6.1 to -0.5)	16 201 (15 399 to 17 249)	-2.8 (-4.8 to -0.5)	552 (395 to 740)	-24.3 (-30.1 to -17.9)	31 (28 to 34)	-38.0 (-45.7 to -30.5)	1 432 (1 318 to 1 556)	-43.5 (-50.0 to -35.8)	1 984 (1 785 to 2 197)	-39.2 (-45.1 to -32.6)
United Arab Emirates	7 610 (7 223 to 8 038)	-9.7 (-12.1 to -7.2)	19 554 (18 635 to 20 672)	-12.8 (-13.8 to -11.8)	707 (510 to 956)	-25.6 (-29.6 to -21.2)	78 (63 to 95)	-18.7 (-40.4 to 8.2)	3 005 (2 432 to 3 638)	-21.7 (-40.9 to 0.8)	3 712 (3 095 to 4 399)	-22.5 (-38.0 to -5.2)
Yemen	10 367 (9 580 to 11 492)	86.8 (74.4 to 106.8)	16 788 (15 805 to 18 037)	-2.6 (-5.0 to 0.4)	787 (587 to 1 011)	-10.3 (-14.6 to -5.6)	127 (111 to 150)	5.3 (-19.2 to 103.1)	6 511 (5 792 to 7 626)	3.4 (-21.8 to 125.9)	7 298 (6 526 to 8 438)	1.8 (-20.7 to 93.8)
**South Asia**	**6 081 (5 753 to 6 434)**	**10.9 (8.7 to 13.2)**	**15 971 (15 266 to 16 775)**	**14.4 (13.3 to 15.6)**	**660 (495 to 857)**	**5.7 (2.4 to 9.3)**	**79 (74 to 84)**	**-22.9 (-29.2 to -15.7)**	**2 960 (2 722 to 3 119)**	**-34.6 (-38.6 to -28.4)**	**3 620 (3 326 to 3 874)**	**-29.7 (-33.7 to -24.0)**
**South Asia**	**6 081 (5 753 to 6 434)**	**10.9 (8.7 to 13.2)**	**15 971 (15 266 to 16 775)**	**14.4 (13.3 to 15.6)**	**660 (495 to 857)**	**5.7 (2.4 to 9.3)**	**79 (74 to 84)**	**-22.9 (-29.2 to -15.7)**	**2 960 (2 722 to 3 119)**	**-34.6 (-38.6 to -28.4)**	**3 620 (3 326 to 3 874)**	**-29.7 (-33.7 to -24.0)**
Bangladesh	5 550 (5 227 to 5 915)	32.3 (28.4 to 36.0)	15 331 (14 553 to 16 193)	32.3 (28.8 to 36.1)	610 (457 to 791)	6.4 (-0.5 to 14.5)	42 (37 to 47)	-48.9 (-56.4 to -38.5)	1 878 (1 628 to 2 153)	-58.3 (-65.4 to -46.2)	2 489 (2 209 to 2 810)	-50.9 (-58.3 to -39.0)
Bhutan	6 017 (5 711 to 6 346)	1.4 (-1.6 to 4.4)	16 121 (15 371 to 16 934)	-0.3 (-2.3 to 1.9)	659 (496 to 859)	-12.4 (-16.6 to -7.7)	50 (40 to 58)	-46.0 (-57.1 to -34.4)	1 761 (1 378 to 2 086)	-55.5 (-66.4 to -44.0)	2 420 (1 958 to 2 811)	-48.6 (-58.9 to -37.9)
India	6 193 (5 864 to 6 553)	6.9 (4.6 to 9.3)	16 028 (15 318 to 16 853)	10.7 (9.7 to 11.9)	657 (488 to 855)	2.8 (-0.2 to 6.0)	85 (78 to 90)	-23.3 (-29.6 to -15.8)	3 070 (2 794 to 3 231)	-34.0 (-38.2 to -28.5)	3 726 (3 396 to 3 990)	-29.5 (-33.7 to -24.2)
Nepal	5 152 (4 878 to 5 447)	9.0 (6.1 to 11.8)	15 793 (14 997 to 16 713)	19.0 (16.0 to 22.4)	675 (510 to 857)	10.5 (6.0 to 15.3)	70 (57 to 85)	-21.0 (-37.7 to 0.4)	2 499 (1 900 to 3 120)	-36.1 (-51.9 to -10.9)	3 173 (2 564 to 3 850)	-29.8 (-44.0 to -8.0)
Pakistan	5 713 (5 414 to 6 045)	33.8 (31.0 to 36.6)	15 928 (15 206 to 16 715)	33.1 (30.6 to 35.8)	716 (538 to 919)	28.6 (24.3 to 33.1)	67 (53 to 80)	0.3 (-16.8 to 22.8)	2 940 (2 324 to 3 513)	-9.5 (-25.5 to 12.2)	3 655 (3 034 to 4 239)	-3.9 (-18.0 to 15.0)
**Southeast Asia, East Asia, and Oceania**	**4 361 (4 123 to 4 597)**	**36.5 (33.4 to 39.7)**	**16 381 (15 567 to 17 224)**	**22.8 (20.8 to 25.1)**	**529 (394 to 693)**	**16.1 (10.7 to 21.8)**	**49 (46 to 50)**	**-41.4 (-44.4 to -38.7)**	**2 040 (1 964 to 2 097)**	**-51.1 (-53.4 to -48.8)**	**2 569 (2 411 to 2 753)**	**-44.5 (-47.3 to -41.5)**
**East Asia**	**5 080 (4 800 to 5 378)**	**49.6 (45.7 to 53.6)**	**19 298 (18 278 to 20 356)**	**28.2 (26.0 to 30.6)**	**578 (428 to 756)**	**21.0 (14.9 to 27.6)**	**46 (43 to 48)**	**-43.8 (-48.2 to -40.7)**	**1 911 (1 820 to 1 981)**	**-55.1 (-58.0 to -52.7)**	**2 489 (2 314 to 2 691)**	**-47.4 (-51.0 to -43.9)**
China	5 111 (4 827 to 5 410)	50.6 (46.6 to 54.6)	19 423 (18 396 to 20 489)	28.8 (26.5 to 31.2)	580 (430 to 758)	21.2 (14.9 to 27.9)	46 (43 to 48)	-44.3 (-48.9 to -41.1)	1 893 (1 798 to 1 963)	-55.8 (-58.8 to -53.4)	2 473 (2 298 to 2 679)	-48.1 (-51.8 to -44.6)
North Korea	3 917 (3 696 to 4 154)	45.1 (40.9 to 49.2)	14 949 (14 150 to 15 816)	21.2 (18.1 to 24.3)	525 (395 to 681)	36.3 (31.5 to 40.9)	61 (50 to 73)	14.4 (-10.6 to 40.9)	3 036 (2 462 to 3 645)	14.9 (-11.2 to 44.0)	3 560 (2 950 to 4 197)	17.6 (-5.7 to 42.7)
Taiwan (Province of China)	4 479 (4 239 to 4 738)	8.0 (3.7 to 12.1)	16 607 (15 750 to 17 571)	4.1 (1.9 to 6.2)	508 (373 to 669)	-1.9 (-5.0 to 1.1)	40 (39 to 43)	-54.2 (-56.5 to -51.8)	1 580 (1 497 to 1 667)	-59.8 (-61.9 to -57.5)	2 088 (1 936 to 2 260)	-53.1 (-55.6 to -50.4)
**Oceania**	**4 717 (4 482 to 4 958)**	**23.8 (21.0 to 26.8)**	**14 781 (14 082 to 15 515)**	**28.7 (26.1 to 31.6)**	**615 (462 to 786)**	**31.2 (27.9 to 34.6)**	**112 (95 to 131)**	**-12.9 (-26.9 to 2.0)**	**5 473 (4 563 to 6 452)**	**-14.1 (-28.6 to 1.4)**	**6 088 (5 149 to 7 084)**	**-11.0 (-24.7 to 3.5)**
American Samoa	4 949 (4 695 to 5 211)	21.3 (18.4 to 24.2)	15 393 (14 651 to 16 196)	24.8 (22.0 to 28.1)	553 (413 to 720)	18.0 (13.8 to 22.7)	52 (48 to 56)	-17.3 (-25.7 to -8.7)	2 201 (2 029 to 2 403)	-19.5 (-27.8 to -10.6)	2 755 (2 516 to 3 007)	-14.0 (-21.6 to -6.1)
Federated States of Micronesia	4 642 (4 384 to 4 916)	35.7 (32.1 to 39.6)	13 958 (13 331 to 14 618)	27.9 (25.2 to 30.6)	574 (426 to 740)	24.5 (19.8 to 29.1)	73 (52 to 89)	-17.7 (-41.9 to 4.8)	3 282 (2 157 to 4 160)	-20.4 (-48.0 to 3.3)	3 856 (2 759 to 4 742)	-15.9 (-41.0 to 6.1)
Fiji	4 361 (4 135 to 4 610)	36.8 (33.1 to 40.8)	13 671 (13 049 to 14 345)	27.8 (25.3 to 30.4)	526 (389 to 679)	27.0 (23.1 to 30.8)	49 (44 to 55)	-8.0 (-21.9 to 7.7)	2 336 (2 068 to 2 648)	-6.6 (-20.8 to 10.1)	2 862 (2 562 to 3 210)	-1.8 (-15.1 to 13.2)
Guam	5 395 (5 118 to 5 680)	34.7 (30.9 to 39.0)	15 842 (15 107 to 16 593)	32.0 (29.7 to 34.3)	548 (400 to 726)	30.5 (26.7 to 34.1)	57 (53 to 62)	1.5 (-8.5 to 12.5)	2 655 (2 441 to 2 881)	4.0 (-6.0 to 15.2)	3 203 (2 938 to 3 479)	7.8 (-1.2 to 17.7)
Kiribati	4 074 (3 835 to 4 328)	45.0 (41.0 to 49.2)	13 647 (12 934 to 14 386)	34.3 (30.9 to 38.0)	543 (408 to 689)	44.7 (39.8 to 49.5)	63 (52 to 74)	-7.9 (-24.4 to 10.1)	3 101 (2 532 to 3 653)	-9.5 (-26.7 to 9.4)	3 645 (3 080 to 4 220)	-4.1 (-20.3 to 13.0)
Marshall Islands	4 720 (4 472 to 4 979)	38.6 (35.0 to 42.4)	14 046 (13 383 to 14 746)	30.3 (27.7 to 32.8)	559 (415 to 724)	29.1 (24.8 to 33.1)	94 (80 to 109)	-12.2 (-24.7 to 2.1)	4 279 (3 618 to 5 057)	-13.9 (-27.0 to 1.0)	4 838 (4 141 to 5 637)	-10.5 (-22.9 to 3.2)
Northern Mariana Islands	5 166 (4 905 to 5 433)	16.4 (13.5 to 19.4)	14 938 (14 240 to 15 648)	13.2 (11.6 to 14.9)	493 (359 to 657)	4.9 (1.6 to 8.4)	51 (45 to 56)	-20.3 (-32.3 to -6.1)	2 110 (1 852 to 2 355)	-19.2 (-32.7 to -2.9)	2 603 (2 335 to 2 900)	-15.6 (-26.9 to -1.7)
Papua New Guinea	4 718 (4 484 to 4 960)	19.3 (16.1 to 22.5)	14 887 (14 168 to 15 629)	27.3 (24.4 to 30.7)	623 (471 to 793)	29.7 (25.8 to 33.7)	126 (103 to 150)	-19.4 (-34.2 to -3.1)	6 181 (5 054 to 7 436)	-21.5 (-36.6 to -5.1)	6 803 (5 652 to 8 041)	-18.5 (-33.0 to -2.8)
Samoa	4 704 (4 451 to 4 968)	25.2 (22.2 to 28.6)	14 639 (13 936 to 15 380)	42.5 (38.2 to 47.5)	616 (464 to 790)	40.5 (35.3 to 45.9)	47 (39 to 57)	-25.8 (-39.8 to -10.5)	1 949 (1 539 to 2 447)	-33.3 (-48.0 to -16.7)	2 565 (2 099 to 3 063)	-23.7 (-37.9 to -8.9)
Solomon Islands	5 154 (4 890 to 5 422)	30.2 (27.0 to 33.2)	15 466 (14 738 to 16 226)	27.9 (25.9 to 30.0)	696 (522 to 889)	29.1 (26.1 to 31.8)	105 (89 to 123)	-17.1 (-33.3 to 2.0)	4 394 (3 637 to 5 212)	-19.8 (-37.2 to 0.2)	5 090 (4 279 to 5 893)	-15.4 (-32.0 to 2.9)
Tonga	4 500 (4 256 to 4 750)	33.8 (30.5 to 37.4)	13 420 (12 823 to 14 045)	32.4 (29.7 to 35.1)	528 (392 to 682)	24.8 (20.1 to 29.8)	48 (42 to 54)	-16.1 (-29.1 to -2.0)	2 093 (1 833 to 2 370)	-14.5 (-28.2 to 0.2)	2 621 (2 330 to 2 940)	-8.7 (-20.7 to 4.5)
Vanuatu	4 990 (4 731 to 5 279)	36.3 (32.9 to 39.5)	15 534 (14 774 to 16 300)	32.9 (30.0 to 36.0)	699 (529 to 890)	39.3 (35.6 to 42.8)	85 (64 to 109)	-5.4 (-28.1 to 26.3)	3 855 (2 813 to 5 085)	-6.7 (-31.6 to 27.3)	4 555 (3 480 to 5 776)	-1.7 (-24.3 to 29.6)
**Southeast Asia**	**2 762 (2 607 to 2 920)**	**5.0 (2.7 to 7.4)**	**9 204 (8 771 to 9 684)**	**13.3 (11.7 to 15.0)**	**395 (296 to 510)**	**3.2 (-0.3 to 7.3)**	**54 (52 to 56)**	**-39.4 (-43.2 to -34.7)**	**2 265 (2 168 to 2 357)**	**-44.9 (-48.1 to -41.0)**	**2 660 (2 514 to 2 810)**	**-40.8 (-44.0 to -37.0)**
Cambodia	3 312 (3 133 to 3 502)	-1.7 (-4.7 to 1.5)	13 815 (12 404 to 15 717)	-13.1 (-17.9 to -7.9)	628 (469 to 812)	-32.9 (-42.1 to -23.9)	98 (81 to 114)	-40.6 (-48.3 to -31.3)	3 701 (3 137 to 4 333)	-49.6 (-57.7 to -40.0)	4 329 (3 741 to 5 012)	-47.7 (-54.9 to -39.0)
Indonesia	2 159 (1 995 to 2 329)	-5.0 (-8.5 to -1.2)	7 961 (7 534 to 8 380)	-1.7 (-3.6 to 0.2)	359 (271 to 458)	-3.8 (-6.3 to -1.1)	39 (37 to 42)	-49.5 (-54.1 to -43.9)	1 560 (1 473 to 1 648)	-56.1 (-59.4 to -51.2)	1 919 (1 787 to 2 050)	-51.1 (-54.5 to -46.2)
Laos	2 667 (2 514 to 2 828)	-12.1 (-15.3 to -8.4)	8 790 (8 403 to 9 208)	7.7 (6.1 to 9.4)	421 (313 to 544)	-0.3 (-4.2 to 3.8)	69 (54 to 80)	-49.2 (-60.3 to -37.7)	3 203 (2 427 to 3 739)	-54.8 (-65.8 to -44.3)	3 624 (2 830 to 4 185)	-51.7 (-62.3 to -41.1)
Malaysia	3 284 (3 099 to 3 477)	29.0 (24.1 to 34.1)	9 556 (9 144 to 10 002)	20.4 (18.4 to 22.4)	394 (284 to 524)	4.1 (-1.9 to 10.4)	52 (47 to 57)	-25.8 (-36.6 to -16.9)	2 015 (1 844 to 2 215)	-27.8 (-36.6 to -19.4)	2 409 (2 200 to 2 644)	-24.0 (-32.2 to -16.5)
Maldives	2 732 (2 560 to 2 917)	19.1 (14.8 to 23.7)	8 596 (8 204 to 9 048)	14.4 (11.5 to 17.5)	324 (237 to 426)	-8.2 (-14.6 to -1.3)	26 (24 to 33)	-65.9 (-69.7 to -58.0)	958 (855 to 1 225)	-72.5 (-75.9 to -65.0)	1 282 (1 138 to 1 573)	-66.6 (-70.6 to -58.9)
Mauritius	2 790 (2 628 to 2 963)	43.7 (39.0 to 48.8)	8 389 (8 019 to 8 805)	21.7 (18.8 to 24.7)	316 (230 to 417)	17.7 (12.9 to 22.4)	38 (36 to 41)	-23.4 (-29.1 to -17.8)	1 746 (1 617 to 1 868)	-23.1 (-28.9 to -17.3)	2 062 (1 915 to 2 214)	-18.8 (-24.1 to -13.4)
Myanmar	4 093 (3 831 to 4 420)	81.0 (71.2 to 93.7)	10 534 (9 813 to 11 430)	39.9 (32.8 to 48.5)	524 (398 to 668)	29.2 (18.6 to 40.8)	77 (68 to 86)	-32.7 (-43.2 to -20.9)	3 393 (3 002 to 3 837)	-39.3 (-49.8 to -27.3)	3 917 (3 487 to 4 375)	-34.7 (-45.2 to -22.8)
Philippines	2 745 (2 596 to 2 900)	1.0 (-2.4 to 4.9)	8 802 (8 397 to 9 235)	20.8 (18.4 to 23.6)	359 (268 to 463)	17.6 (13.3 to 22.0)	56 (49 to 64)	-20.5 (-30.4 to -8.9)	2 536 (2 238 to 2 891)	-23.5 (-33.4 to -12.4)	2 895 (2 575 to 3 260)	-20.1 (-29.2 to -9.8)
Sri Lanka	2 871 (2 723 to 3 039)	-58.4 (-61.3 to -55.4)	14 143 (12 470 to 16 343)	54.6 (42.9 to 67.4)	548 (409 to 715)	25.4 (14.2 to 41.8)	62 (52 to 72)	-55.2 (-62.1 to -47.5)	2 302 (1 900 to 2 728)	-62.4 (-68.9 to -55.6)	2 850 (2 430 to 3 327)	-56.5 (-62.6 to -49.9)
Seychelles	3 033 (2 873 to 3 204)	37.1 (33.2 to 40.9)	9 354 (8 963 to 9 795)	26.4 (24.2 to 28.8)	362 (264 to 481)	14.4 (8.6 to 20.5)	55 (52 to 59)	-19.9 (-25.7 to -13.1)	2 332 (2 187 to 2 479)	-18.7 (-25.3 to -12.3)	2 694 (2 515 to 2 883)	-15.4 (-21.4 to -9.5)
Thailand	3 289 (3 115 to 3 476)	14.3 (10.1 to 18.7)	10 193 (9 724 to 10 708)	12.4 (10.9 to 14.0)	398 (289 to 527)	-4.5 (-9.7 to 1.2)	61 (55 to 67)	-38.5 (-46.5 to -30.3)	2 973 (2 675 to 3 267)	-38.8 (-46.7 to -31.0)	3 371 (3 026 to 3 703)	-36.1 (-43.5 to -28.7)
Timor-Leste	2 456 (2 318 to 2 607)	-52.2 (-55.7 to -48.6)	18 400 (14 992 to 22 830)	16.0 (13.8 to 18.1)	907 (655 to 1 223)	-8.2 (-15.2 to -0.7)	44 (30 to 54)	-55.0 (-69.5 to -45.8)	1 894 (1 044 to 2 326)	-63.5 (-79.5 to -54.6)	2 801 (1 998 to 3 355)	-54.7 (-67.6 to -47.0)
Vietnam	3 003 (2 851 to 3 181)	30.8 (26.5 to 35.0)	9 196 (8 799 to 9 627)	31.5 (29.6 to 33.6)	374 (275 to 494)	11.6 (5.0 to 17.9)	68 (60 to 75)	-25.1 (-36.0 to -13.0)	2 559 (2 220 to 2 820)	-34.6 (-44.9 to -23.1)	2 933 (2 578 to 3 225)	-30.9 (-41.0 to -20.3)
**Sub-Saharan Africa**	**5 823 (5 537 to 6 136)**	**-18.4 (-20.1 to -16.5)**	**16 983 (16 147 to 17 979)**	**-1.5 (-2.8 to -0.1)**	**752 (568 to 959)**	**-9.8 (-11.7 to -7.6)**	**75 (70 to 80)**	**-34.9 (-38.9 to -29.4)**	**2 977 (2 783 to 3 187)**	**-42.6 (-47.0 to -35.3)**	**3 729 (3 453 to 4 003)**	**-38.1 (-42.5 to -31.5)**
**Central sub-Saharan Africa**	**5 707 (5 410 to 6 010)**	**-2.2 (-4.1 to -0.4)**	**18 932 (17 732 to 20 520)**	**10.0 (6.3 to 14.2)**	**815 (619 to 1 032)**	**1.0 (-2.1 to 4.9)**	**82 (73 to 92)**	**-26.9 (-35.8 to -10.8)**	**3 761 (3 332 to 4 245)**	**-33.4 (-42.9 to -14.7)**	**4 577 (4 090 to 5 109)**	**-29.1 (-38.2 to -12.0)**
Angola	5 565 (5 281 to 5 870)	-36.2 (-40.1 to -33.0)	21 900 (19 878 to 24 708)	-8.2 (-9.9 to -6.7)	955 (718 to 1 212)	-26.2 (-30.6 to -21.8)	75 (65 to 87)	-55.6 (-63.9 to -38.1)	3 278 (2 803 to 3 849)	-63.6 (-71.2 to -45.6)	4 234 (3 689 to 4 901)	-58.9 (-66.3 to -42.6)
Central African Republic	9 025 (8 369 to 9 911)	78.5 (67.3 to 96.4)	16 973 (15 870 to 18 419)	21.4 (14.6 to 30.3)	879 (652 to 1 129)	33.4 (23.1 to 48.2)	201 (161 to 235)	27.0 (5.1 to 79.0)	10 134 (7 937 to 12 026)	31.9 (6.8 to 113.4)	11 013 (8 808 to 12 914)	32.0 (8.6 to 105.1)
Congo (Brazzaville)	5 498 (5 217 to 5 810)	-5.2 (-7.6 to -3.0)	19 128 (17 832 to 20 865)	14.2 (7.2 to 23.6)	850 (639 to 1 089)	10.7 (3.2 to 21.4)	77 (65 to 92)	-37.4 (-49.4 to -22.5)	3 295 (2 672 to 3 994)	-43.4 (-55.4 to -27.0)	4 146 (3 471 to 4 893)	-37.1 (-48.4 to -22.4)
DR Congo	5 577 (5 273 to 5 892)	9.7 (6.8 to 12.0)	18 178 (17 166 to 19 421)	16.5 (11.1 to 22.8)	770 (585 to 975)	12.4 (6.9 to 20.3)	78 (67 to 91)	-15.4 (-28.5 to 6.5)	3 624 (3 035 to 4 291)	-21.0 (-35.1 to 6.2)	4 394 (3 787 to 5 071)	-16.7 (-29.6 to 7.2)
Equatorial Guinea	5 621 (5 331 to 5 940)	2.8 (-0.1 to 5.8)	16 787 (15 989 to 17 637)	5.2 (3.4 to 6.9)	615 (459 to 801)	-18.7 (-23.4 to -13.3)	56 (40 to 78)	-61.9 (-73.0 to -47.3)	2 312 (1 604 to 3 229)	-68.7 (-78.3 to -54.6)	2 927 (2 201 to 3 904)	-64.1 (-73.4 to -50.8)
Gabon	5 916 (5 613 to 6 250)	-12.2 (-14.2 to -10.0)	17 082 (16 362 to 17 870)	-13.1 (-14.3 to -12.1)	730 (547 to 947)	-19.0 (-21.1 to -16.8)	72 (61 to 84)	-30.0 (-41.7 to -15.7)	3 018 (2 442 to 3 602)	-34.9 (-47.5 to -20.3)	3 748 (3 161 to 4 374)	-32.3 (-43.0 to -20.3)
**Eastern sub-Saharan Africa**	**6 666 (6 317 to 7 045)**	**-31.8 (-34.0 to -29.5)**	**20 559 (19 353 to 22 010)**	**-1.2 (-2.6 to 0.1)**	**933 (703 to 1 184)**	**-9.8 (-12.3 to -7.2)**	**77 (72 to 83)**	**-45.1 (-50.2 to -38.6)**	**2 798 (2 611 to 3 032)**	**-53.6 (-58.5 to -45.8)**	**3 731 (3 438 to 4 081)**	**-47.2 (-52.3 to -39.9)**
Burundi	6 323 (5 991 to 6 690)	-10.5 (-13.0 to -8.1)	36 584 (30 987 to 43 074)	74.3 (47.3 to 105.8)	1 790 (1 298 to 2 346)	87.6 (56.0 to 136.2)	88 (74 to 105)	-34.3 (-45.2 to -19.4)	3 099 (2 613 to 3 757)	-39.1 (-50.1 to -22.7)	4 890 (4 156 to 5 817)	-19.1 (-31.5 to -2.1)
Comoros	6 361 (6 032 to 6 721)	-14.0 (-16.3 to -11.7)	15 735 (15 023 to 16 593)	-15.8 (-16.9 to -14.6)	787 (582 to 1 014)	-22.0 (-24.2 to -19.9)	67 (56 to 82)	-35.5 (-46.1 to -22.6)	2 334 (1 905 to 2 948)	-42.6 (-52.8 to -28.9)	3 121 (2 631 to 3 805)	-38.5 (-47.3 to -27.2)
Djibouti	6 630 (6 279 to 7 026)	-20.6 (-23.7 to -17.9)	18 435 (17 559 to 19 396)	-4.3 (-6.1 to -2.2)	857 (642 to 1 103)	-14.6 (-17.3 to -11.5)	69 (52 to 94)	-32.9 (-50.1 to -9.5)	2 416 (1 758 to 3 416)	-42.5 (-58.0 to -15.8)	3 273 (2 583 to 4 275)	-37.1 (-50.4 to -15.3)
Eritrea	6 555 (6 217 to 6 911)	-90.1 (-90.8 to -89.3)	36 752 (31 740 to 43 448)	-31.9 (-34.0 to -29.7)	1 857 (1 367 to 2 448)	-52.6 (-57.5 to -46.3)	105 (87 to 125)	-91.6 (-93.0 to -89.9)	4 025 (3 255 to 4 929)	-94.1 (-95.2 to -92.7)	5 882 (4 940 to 6 952)	-91.9 (-93.1 to -90.4)
Ethiopia	6 328 (5 973 to 6 717)	-54.4 (-57.0 to -51.8)	19 435 (18 197 to 20 857)	-6.4 (-8.8 to -3.9)	951 (709 to 1 211)	-14.4 (-17.5 to -11.0)	69 (63 to 77)	-61.0 (-66.7 to -52.6)	2 378 (2 142 to 2 650)	-68.6 (-73.9 to -60.3)	3 328 (2 982 to 3 708)	-61.7 (-67.2 to -53.5)
Kenya	7 429 (7 000 to 7 889)	8.6 (6.7 to 10.5)	21 469 (20 436 to 22 633)	8.4 (7.2 to 9.7)	788 (588 to 1 033)	6.6 (5.1 to 8.3)	76 (69 to 84)	-11.4 (-26.3 to -1.2)	2 568 (2 332 to 2 948)	-11.9 (-23.0 to -2.3)	3 356 (3 042 to 3 800)	-8.1 (-18.1 to -0.2)
Madagascar	6 465 (6 138 to 6 818)	-8.3 (-10.8 to -6.0)	16 933 (16 158 to 17 824)	-11.8 (-12.9 to -10.8)	792 (588 to 1 018)	-16.3 (-18.4 to -14.3)	65 (54 to 76)	-31.4 (-42.1 to -19.9)	2 356 (1 954 to 2 831)	-39.9 (-50.4 to -26.2)	3 148 (2 686 to 3 677)	-35.3 (-44.1 to -24.2)
Malawi	5 800 (5 499 to 6 134)	-1.6 (-3.8 to 0.7)	16 794 (16 002 to 17 682)	1.3 (-0.9 to 3.8)	683 (511 to 878)	-7.1 (-9.6 to -4.6)	67 (59 to 76)	-31.7 (-47.6 to 20.7)	2 328 (1 998 to 2 712)	-38.6 (-56.3 to 46.1)	3 011 (2 616 to 3 431)	-33.5 (-50.1 to 27.9)
Mozambique	6 434 (6 096 to 6 789)	-14.6 (-18.2 to -11.5)	21 221 (19 477 to 23 595)	-18.1 (-22.0 to -14.0)	893 (673 to 1 134)	-40.4 (-49.4 to -31.6)	95 (83 to 109)	-25.7 (-36.8 to -13.1)	3 477 (2 998 to 4 024)	-35.8 (-45.8 to -24.0)	4 370 (3 863 to 4 951)	-36.8 (-44.8 to -27.1)
Rwanda	6 159 (5 840 to 6 536)	-42.7 (-46.6 to -39.4)	29 893 (26 503 to 34 047)	44.4 (27.5 to 63.6)	1 649 (1 195 to 2 229)	60.5 (33.8 to 102.5)	79 (66 to 96)	-57.9 (-64.6 to -48.8)	2 694 (2 228 to 3 358)	-65.6 (-71.5 to -56.1)	4 342 (3 642 to 5 196)	-51.0 (-58.7 to -40.5)
Somalia	9 781 (9 149 to 10 606)	-3.6 (-5.0 to -2.2)	21 549 (19 898 to 23 731)	9.3 (5.9 to 13.3)	1 141 (848 to 1 462)	5.7 (2.6 to 8.8)	150 (120 to 189)	-7.0 (-30.0 to 39.8)	6 224 (5 056 to 7 791)	-11.8 (-35.8 to 44.7)	7 365 (6 143 to 8 961)	-9.5 (-31.0 to 36.7)
South Sudan	11 529 (10 748 to 12 623)	29.3 (25.3 to 34.3)	23 474 (21 754 to 25 733)	26.0 (18.7 to 36.0)	1 167 (867 to 1 495)	29.1 (21.1 to 41.3)	141 (121 to 168)	15.5 (-12.1 to 64.5)	6 224 (5 439 to 7 291)	25.0 (-6.7 to 94.3)	7 392 (6 536 to 8 440)	25.7 (-2.2 to 80.5)
Tanzania	6 125 (5 809 to 6 474)	1.1 (-1.5 to 3.4)	16 938 (16 219 to 17 807)	-1.2 (-3.4 to 0.8)	739 (554 to 947)	-4.2 (-6.6 to -1.9)	61 (54 to 69)	-23.7 (-36.8 to 8.7)	2 258 (1 958 to 2 610)	-25.6 (-41.1 to 29.0)	2 997 (2 639 to 3 382)	-21.3 (-34.5 to 18.8)
Uganda	5 976 (5 673 to 6 330)	-2.6 (-5.1 to -0.1)	21 577 (19 943 to 23 723)	-11.0 (-14.6 to -7.5)	898 (680 to 1 134)	-19.3 (-29.2 to -11.2)	71 (61 to 84)	-18.4 (-33.4 to 0.0)	2 468 (2 095 to 2 997)	-24.8 (-38.5 to -7.4)	3 366 (2 918 to 3 962)	-23.4 (-34.5 to -10.0)
Zambia	6 162 (5 838 to 6 519)	-8.5 (-10.9 to -6.1)	17 344 (16 573 to 18 210)	-6.6 (-8.3 to -4.8)	761 (566 to 975)	-8.7 (-10.5 to -6.9)	75 (66 to 84)	-35.7 (-46.4 to -16.2)	2 628 (2 289 to 3 019)	-41.9 (-53.8 to -11.2)	3 389 (2 980 to 3 830)	-36.8 (-47.7 to -10.5)
**Southern sub-Saharan Africa**	**5 685 (5 390 to 6 004)**	**-8.2 (-9.7 to -6.6)**	**15 448 (14 848 to 16 208)**	**-12.7 (-13.8 to -11.7)**	**627 (464 to 819)**	**-21.3 (-23.3 to -19.0)**	**87 (81 to 92)**	**-36.8 (-43.1 to -31.8)**	**4 022 (3 741 to 4 283)**	**-41.3 (-46.3 to -37.0)**	**4 649 (4 306 to 4 988)**	**-39.2 (-43.7 to -35.3)**

Location	Incidence (95% UI)		Prevalence (95% UI)		Years lived with disability (95% UI)		Deaths (95% UI)		Years of life lost (95% UI)		Disability adjusted life years (95% UI)	
	2017 age-standardised rates per 100 000	Percentage change in age-standardised rates between 1990 and 2017	2017 age-standardised rates per 100 000	Percentage change in age-standardised rates between 1990 and 2017	2017 age-standardised rates per 100 000	Percentage change in age-standardised rates between 1990 and 2017	2017 age-standardised rates per 100 000	Percentage change in age-standardised rates between 1990 and 2017	2017 age-standardised rates per 100 000	Percentage change in age-standardised rates between 1990 and 2017	2017 age-standardised rates per 100 000	Percentage change in age-standardised rates between 1990 and 2017
Botswana	5 380<br>(5 114 to 5 680)	-1.3<br>(-3.5 to 1.1)	14 299<br>(13 743 to 14 998)	-0.7<br>(-1.8 to 0.4)	564<br>(417 to 741)	-10.9<br>(-14.5 to -7.2)	51<br>(46 to 58)	-29.0<br>(-42.6 to -13.5)	2 185<br>(1 929 to 2 487)	-30.6<br>(-45.0 to -13.3)	2 749<br>(2 449 to 3 106)	-27.3<br>(-40.3 to -12.5)
Lesotho	5 879<br>(5 613 to 6 176)	16.2<br>(13.7 to 18.6)	15 573<br>(14 971 to 16 349)	15.0<br>(13.8 to 16.2)	669<br>(498 to 864)	6.7<br>(3.7 to 10.0)	156<br>(126 to 184)	25.8<br>(-1.5 to 56.5)	7 282<br>(5 784 to 8 713)	30.3<br>(0.9 to 63.8)	7 951<br>(6 425 to 9 407)	28.0<br>(1.4 to 57.4)
Namibia	5 613<br>(5 352 to 5 903)	-5.3<br>(-7.6 to -3.1)	18 500<br>(17 110 to 20 301)	-18.9<br>(-23.0 to -14.9)	752<br>(563 to 963)	-38.7<br>(-47.2 to -31.3)	72<br>(59 to 87)	-37.4<br>(-49.0 to -23.8)	3 205<br>(2 551 to 3 994)	-38.0<br>(-50.6 to -22.8)	3 957<br>(3 288 to 4 766)	-38.1<br>(-48.4 to -25.9)
South Africa	5 790<br>(5 477 to 6 125)	-12.6<br>(-14.4 to -10.9)	15 158<br>(14 554 to 15 917)	-17.5<br>(-18.9 to -16.3)	612<br>(452 to 808)	-27.7<br>(-30.1 to -25.1)	84<br>(79 to 91)	-45.5<br>(-50.3 to -41.0)	3 965<br>(3 648 to 4 299)	-50.6<br>(-54.4 to -46.5)	4 577<br>(4 219 to 4 978)	-48.4<br>(-52.1 to -44.6)
Swaziland	5 832<br>(5 553 to 6 137)	4.1<br>(1.9 to 6.3)	15 463<br>(14 851 to 16 255)	-1.5<br>(-2.6 to -0.6)	644<br>(474 to 837)	-10.5<br>(-13.5 to -7.3)	117<br>(94 to 142)	-6.4<br>(-29.0 to 18.4)	5 532<br>(4 443 to 6 744)	-4.1<br>(-27.3 to 21.1)	6 176<br>(5 081 to 7 375)	-4.8<br>(-25.6 to 17.5)
Zimbabwe	5 388<br>(5 128 to 5 659)	9.6<br>(7.6 to 11.8)	16 182<br>(15 538 to 16 927)	7.7<br>(5.4 to 9.9)	668<br>(503 to 852)	18.4<br>(15.5 to 21.7)	103<br>(89 to 118)	19.6<br>(-3.9 to 42.8)	4 144<br>(3 539 to 4 732)	24.8<br>(-0.8 to 51.1)	4 813<br>(4 167 to 5 449)	23.8<br>(1.9 to 46.0)
**Western sub-Saharan Africa**	**5 180<br>(4 922 to 5 455)**	**-1.8<br>(-3.6 to 0.0)**	**13 654<br>(13 089 to 14 314)**	**-1.2<br>(-2.5 to 0.2)**	**607<br>(453 to 783)**	**-8.7<br>(-11.0 to -6.2)**	**69<br>(62 to 77)**	**-19.9<br>(-27.9 to -9.9)**	**2 676<br>(2 404 to 2 988)**	**-27.9<br>(-35.3 to -16.9)**	**3 282<br>(2 956 to 3 628)**	**-25.0<br>(-31.5 to -15.5)**
Benin	5 239<br>(4 976 to 5 519)	-2.0<br>(-4.2 to -0.1)	13 489<br>(12 960 to 14 132)	-4.9<br>(-5.8 to -4.1)	659<br>(488 to 850)	-11.6<br>(-14.1 to -9.0)	95<br>(76 to 118)	-24.5<br>(-36.8 to -8.9)	3 565<br>(2 814 to 4 471)	-33.0<br>(-45.2 to -18.5)	4 224<br>(3 450 to 5 160)	-30.4<br>(-40.9 to -17.6)
Burkina Faso	5 185<br>(4 933 to 5 455)	-0.6<br>(-3.0 to 1.5)	12 176<br>(11 669 to 12 785)	-1.1<br>(-1.9 to -0.2)	595<br>(442 to 771)	-5.8<br>(-8.8 to -3.0)	94<br>(83 to 106)	-14.9<br>(-26.5 to 1.2)	3 585<br>(3 043 to 4 244)	-18.2<br>(-30.6 to -1.9)	4 181<br>(3 636 to 4 849)	-16.7<br>(-27.8 to -2.4)
Cameroon	5 226<br>(4 971 to 5 502)	-3.3<br>(-5.6 to -0.9)	13 864<br>(13 266 to 14 531)	-7.3<br>(-8.3 to -6.2)	614<br>(457 to 791)	-15.0<br>(-17.5 to -12.6)	79<br>(65 to 93)	-20.3<br>(-33.6 to -5.5)	2 850<br>(2 325 to 3 403)	-25.4<br>(-39.3 to -10.2)	3 464<br>(2 885 to 4 059)	-23.8<br>(-35.8 to -11.0)
Cape Verde	5 090<br>(4 828 to 5 373)	7.5<br>(5.4 to 9.5)	13 541<br>(12 960 to 14 187)	3.3<br>(2.2 to 4.5)	522<br>(385 to 689)	-9.6<br>(-13.5 to -5.4)	58<br>(53 to 63)	-0.2<br>(-12.8 to 12.4)	2 542<br>(2 293 to 2 810)	-7.3<br>(-19.7 to 5.5)	3 064<br>(2 753 to 3 383)	-7.7<br>(-18.0 to 2.8)
Chad	5 300<br>(5 044 to 5 573)	-7.8<br>(-11.1 to -5.0)	16 731<br>(15 686 to 18 109)	3.4<br>(1.3 to 5.4)	776<br>(584 to 983)	-8.1<br>(-13.7 to -3.1)	89<br>(75 to 108)	-3.9<br>(-17.2 to 10.7)	3 577<br>(3 028 to 4 199)	-12.8<br>(-27.0 to 3.1)	4 353<br>(3 770 to 5 057)	-12.0<br>(-23.8 to 1.0)
Cote d'Ivoire	5 122<br>(4 871 to 5 389)	-2.9<br>(-5.1 to -0.7)	13 301<br>(12 753 to 13 939)	-3.3<br>(-4.4 to -2.2)	645<br>(478 to 832)	-4.8<br>(-6.9 to -2.8)	89<br>(77 to 102)	-14.1<br>(-27.2 to 1.9)	3 329<br>(2 849 to 3 867)	-17.7<br>(-31.0 to -1.2)	3 974<br>(3 456 to 4 557)	-15.8<br>(-27.4 to -1.9)
The Gambia	5 027<br>(4 774 to 5 298)	2.4<br>(0.4 to 4.4)	12 715<br>(12 188 to 13 356)	-4.2<br>(-5.5 to -2.9)	592<br>(438 to 764)	-11.2<br>(-14.6 to -8.2)	72<br>(61 to 86)	-7.9<br>(-22.9 to 10.0)	2 416<br>(1 899 to 3 051)	-18.0<br>(-33.4 to 1.5)	3 008<br>(2 469 to 3 663)	-16.7<br>(-29.8 to -1.1)
Ghana	5 422<br>(5 160 to 5 696)	7.4<br>(5.2 to 9.8)	13 929<br>(13 365 to 14 591)	4.9<br>(3.9 to 5.9)	606<br>(453 to 785)	-4.0<br>(-7.3 to -0.6)	76<br>(67 to 85)	-3.0<br>(-20.1 to 15.0)	2 646<br>(2 264 to 3 056)	-11.8<br>(-26.7 to 5.8)	3 252<br>(2 843 to 3 725)	-10.4<br>(-23.1 to 4.1)
Guinea	5 149<br>(4 908 to 5 404)	-0.8<br>(-2.8 to 1.3)	13 293<br>(12 743 to 13 921)	-2.8<br>(-3.9 to -1.6)	647<br>(481 to 831)	-10.5<br>(-13.0 to -7.9)	85<br>(72 to 100)	-13.4<br>(-26.8 to 1.0)	3 238<br>(2 713 to 3 779)	-28.9<br>(-41.8 to -12.5)	3 885<br>(3 331 to 4 489)	-26.4<br>(-37.7 to -12.0)
Guinea-Bissau	4 868<br>(4 630 to 5 128)	-11.3<br>(-13.5 to -9.3)	13 173<br>(12 607 to 13 816)	-9.1<br>(-10.6 to -7.2)	632<br>(471 to 810)	-14.0<br>(-16.5 to -11.4)	99<br>(85 to 116)	-31.7<br>(-44.2 to -16.9)	3 796<br>(3 132 to 4 602)	-37.5<br>(-50.1 to -20.5)	4 427<br>(3 753 to 5 218)	-34.9<br>(-46.7 to -19.9)
Liberia	4 617<br>(4 377 to 4 880)	-79.6<br>(-82.1 to -77.2)	18 264<br>(16 233 to 20 947)	11.5<br>(1.5 to 24.1)	825<br>(620 to 1 053)	-6.9<br>(-16.5 to 4.6)	61<br>(52 to 73)	-79.6<br>(-82.6 to -75.8)	2 173<br>(1 789 to 2 721)	-86.4<br>(-88.8 to -82.8)	2 997<br>(2 571 to 3 578)	-82.2<br>(-84.7 to -78.5)
Mali	5 380<br>(5 108 to 5 655)	-3.4<br>(-5.5 to -1.6)	16 137<br>(15 209 to 17 315)	7.7<br>(2.6 to 14.3)	702<br>(529 to 891)	-2.8<br>(-8.2 to 4.1)	77<br>(64 to 97)	-31.1<br>(-41.8 to -15.6)	3 394<br>(2 845 to 4 117)	-35.7<br>(-46.6 to -18.6)	4 097<br>(3 518 to 4 868)	-31.7<br>(-41.9 to -16.8)
Mauritania	4 910<br>(4 664 to 5 189)	-9.7<br>(-12.0 to -7.5)	12 320<br>(11 820 to 12 944)	-9.6<br>(-10.5 to -8.7)	560<br>(415 to 725)	-18.8<br>(-21.5 to -15.9)	62<br>(54 to 72)	-35.8<br>(-45.6 to -22.9)	2 276<br>(1 949 to 2 680)	-41.2<br>(-50.9 to -25.8)	2 836<br>(2 465 to 3 307)	-37.8<br>(-46.3 to -24.6)
Niger	5 124<br>(4 868 to 5 403)	-5.7<br>(-7.8 to -3.5)	12 742<br>(12 223 to 13 353)	-7.1<br>(-8.0 to -6.1)	625<br>(461 to 805)	-13.9<br>(-16.4 to -11.5)	71<br>(58 to 92)	-32.1<br>(-43.9 to -15.7)	2 858<br>(2 295 to 3 583)	-45.2<br>(-56.5 to -26.3)	3 483<br>(2 886 to 4 227)	-41.4<br>(-52.2 to -24.6)
Nigeria	5 164<br>(4 898 to 5 447)	5.5<br>(3.2 to 7.9)	13 369<br>(12 822 to 14 010)	-2.6<br>(-4.5 to -1.0)	565<br>(422 to 733)	-10.3<br>(-13.4 to -7.0)	55<br>(45 to 71)	-20.4<br>(-35.8 to 2.1)	2 229<br>(1 801 to 2 795)	-26.1<br>(-38.9 to -6.1)	2 794<br>(2 342 to 3 383)	-23.4<br>(-34.2 to -6.6)
Sao Tome and Principe	5 436<br>(5 148 to 5 760)	5.2<br>(2.2 to 8.0)	14 152<br>(13 514 to 14 912)	4.4<br>(3.2 to 5.6)	627<br>(468 to 812)	-7.8<br>(-11.1 to -4.7)	52<br>(43 to 61)	-3.2<br>(-20.7 to 15.6)	2 035<br>(1 754 to 2 352)	-21.2<br>(-34.6 to -5.2)	2 661<br>(2 321 to 3 031)	-18.4<br>(-29.6 to -5.5)
Senegal	4 989<br>(4 730 to 5 255)	-6.3<br>(-8.4 to -4.0)	12 700<br>(12 195 to 13 316)	-5.9<br>(-6.8 to -4.8)	618<br>(460 to 798)	-10.2<br>(-12.4 to -8.0)	67<br>(58 to 79)	-18.7<br>(-30.0 to -5.8)	2 342<br>(2 003 to 2 900)	-30.5<br>(-42.0 to -13.2)	2 960<br>(2 574 to 3 571)	-27.1<br>(-36.8 to -12.6)
Sierra Leone	5 148<br>(4 890 to 5 418)	2.9<br>(0.5 to 5.2)	17 024<br>(15 583 to 18 918)	22.8<br>(13.1 to 35.3)	781<br>(586 to 996)	16.2<br>(7.0 to 30.2)	76<br>(65 to 89)	-16.0<br>(-30.1 to 4.7)	2 978<br>(2 505 to 3 503)	-30.4<br>(-43.7 to -9.8)	3 759<br>(3 263 to 4 335)	-24.1<br>(-37.0 to -5.9)
Togo	4 913<br>(4 672 to 5 186)	-0.7<br>(-2.9 to 1.5)	12 699<br>(12 202 to 13 291)	-4.6<br>(-5.9 to -3.4)	588<br>(435 to 756)	-11.0<br>(-13.6 to -8.1)	73<br>(62 to 85)	-14.9<br>(-29.6 to 2.0)	2 658<br>(2 205 to 3 193)	-23.6<br>(-37.7 to -5.0)	3 246<br>(2 757 to 3 783)	-21.6<br>(-34.0 to -6.0)
