# Supplementary material for: Global injury morbidity and mortality from 1990 to 2017: results from the Global Burden of Disease Study 2017
Source: Inj Prev. 2020 Apr 24;26(Suppl 1):i96–i114. doi: 10.1136/injuryprev-2019-043494 (PMC7571366; doi:10.1136/injuryprev-2019-043494)
Supplement: Supplementary data [file injuryprev-2019-043494supp005.pdf]

Appendix Table 3: All-age incidence, prevalence, YLD, death, YLL, and DALY counts in 2017 and percent change between 1990 and 2017 for all injuries by location

Location	Incidence (95% UI)		Prevalence (95% UI)		Years lived with disability (95% UI)		Deaths (95% UI)		Years of life lost (95% UI)		Disability adjusted life years (95% UI)	
	2017 all-age counts	Percentage change in age-standardised rates between 1990 and 2017	2017 all-age counts	Percentage change in age-standardised rates between 1990 and 2017	2017 all-age counts	Percentage change in age-standardised rates between 1990 and 2017	2017 all-age counts	Percentage change in age-standardised rates between 1990 and 2017	2017 all-age counts	Percentage change in age-standardised rates between 1990 and 2017	2017 all-age counts	Percentage change in age-standardised rates between 1990 and 2017
**Global**	**520 710 288 (493 430 247 to 547 988 635)**	**47.1 (44.3 to 50.0)**	**1 507 481 369 (1 439 757 976 to 1 587 209**	**61.1 (59.3 to 62.7)**	**57 174 469 (42 073 855 to 75 427 035)**	**52.7 (49.3 to 56.4)**	**4 484 722 (4 332 010 to 4 585 554)**	**5.3 (2.0 to 9.5)**	**195 231 148 (188 807 653 to 199 825 464)**	**-15.9 (-18.9 to -11.3)**	**252 405 616 (236 154 016 to 271 424 808)**	**-6.4 (-10.4 to -1.3)**
**Low SDI**	**72 885 096 (68 891 884 to 77 185 429)**	**70.6 (66.8 to 74.6)**	**174 033 957 (165 195 975 to 183 911 869)**	**109.9 (107.7 to 112.3)**	**7 869 353 (5 908 508 to 10 058 535)**	**86.5 (80.7 to 93.2)**	**774 202 (720 525 to 816 602)**	**14.4 (4.5 to 34.1)**	**40 028 724 (37 124 919 to 42 500 309)**	**-3.7 (-14.0 to 16.8)**	**47 898 077 (44 443 855 to 51 310 290)**	**4.6 (-5.7 to 24.4)**
**Low-middle SDI**	**94 465 166 (89 588 836 to 99 666 566)**	**88.6 (85.0 to 92.4)**	**234 907 327 (223 838 097 to 247 298 914)**	**99.2 (96.9 to 101.7)**	**9 800 151 (7 361 900 to 12 594 725)**	**80.9 (74.6 to 88.2)**	**1 097 431 (1 039 119 to 1 144 390)**	**26.4 (19.4 to 35.0)**	**52 835 942 (50 124 360 to 55 216 581)**	**5.9 (-0.5 to 15.4)**	**62 636 092 (58 952 318 to 66 167 370)**	**13.3 (6.9 to 22.4)**
**Middle SDI**	**100 192 881 (94 928 349 to 105 664 970)**	**73.1 (68.9 to 77.6)**	**329 063 141 (314 071 181 to 345 509 181)**	**86.2 (81.8 to 90.7)**	**11 603 119 (8 628 144 to 15 188 235)**	**76.3 (67.9 to 85.0)**	**1 264 366 (1 214 871 to 1 300 348)**	**3.0 (-0.8 to 6.8)**	**54 390 292 (52 834 200 to 55 625 513)**	**-22.4 (-25.1 to -19.7)**	**65 993 411 (62 402 359 to 70 061 395)**	**-13.9 (-17.4 to -10.3)**
**High-middle SDI**	**115 473 653 (109 140 522 to 122 138 814)**	**29.5 (26.4 to 32.7)**	**380 202 778 (362 456 386 to 401 709 999)**	**51.2 (49.3 to 53.0)**	**13 249 892 (9 669 351 to 17 720 393)**	**35.7 (30.7 to 40.9)**	**767 257 (746 484 to 785 770)**	**-14.2 (-17.4 to -11.3)**	**30 753 348 (30 023 601 to 31 429 972)**	**-33.6 (-35.7 to -31.7)**	**44 003 240 (40 207 218 to 48 657 221)**	**-21.6 (-25.3 to -17.5)**
**High SDI**	**136 150 513 (128 763 920 to 144 335 914)**	**20.1 (16.3 to 24.0)**	**382 958 554 (362 920 799 to 409 374 635)**	**26.1 (24.6 to 27.6)**	**14 450 190 (10 393 630 to 19 652 633)**	**27.2 (25.5 to 29.0)**	**565 805 (556 294 to 575 898)**	**-1.6 (-3.2 to 0.1)**	**16 610 469 (16 348 973 to 16 873 311)**	**-28.3 (-29.4 to -27.2)**	**31 060 659 (27 044 551 to 36 248 204)**	**-10.1 (-14.0 to -6.1)**
**Central Europe, Eastern Europe, and Central Asia**	**66 809 666 (62 433 803 to 71 919 666)**	**-3.1 (-5.7 to -0.3)**	**187 153 231 (176 611 329 to 199 202 251)**	**10.1 (9.3 to 11.0)**	**7 359 329 (5 244 668 to 9 994 188)**	**1.7 (-1.0 to 4.7)**	**315 624 (310 296 to 321 210)**	**-22.3 (-23.4 to -21.1)**	**12 752 145 (12 518 611 to 12 998 694)**	**-31.7 (-32.8 to -30.6)**	**20 111 474 (17 997 599 to 22 762 723)**	**-22.4 (-24.9 to -19.9)**
**Central Asia**	**9 384 710 (8 752 705 to 10 087 550)**	**30.4 (26.1 to 35.1)**	**23 341 127 (21 841 620 to 25 041 922)**	**47.8 (46.1 to 49.5)**	**931 546 (670 869 to 1 250 960)**	**32.2 (27.7 to 37.0)**	**43 205 (41 318 to 45 534)**	**-12.2 (-16.1 to -7.9)**	**2 159 328 (2 061 334 to 2 280 537)**	**-22.4 (-26.2 to -18.3)**	**3 090 874 (2 810 228 to 3 413 210)**	**-11.3 (-15.7 to -7.3)**
Armenia	306 525 (285 048 to 329 970)	-28.5 (-31.7 to -25.0)	1 020 865 (949 384 to 1 102 649)	-10.5 (-12.6 to -8.5)	36 857 (26 587 to 49 431)	-22.7 (-27.3 to -18.1)	1 110 (1 070 to 1 152)	-52.6 (-55.0 to -50.0)	41 671 (40 152 to 43 340)	-65.5 (-67.4 to -63.5)	78 529 (68 089 to 91 121)	-53.3 (-56.8 to -49.9)
Azerbaijan	1 054 398 (977 311 to 1 139 058)	45.1 (38.9 to 51.5)	2 930 027 (2 732 738 to 3 140 609)	80.6 (76.8 to 85.1)	113 430 (81 777 to 152 833)	52.7 (45.0 to 61.1)	3 099 (2 779 to 3 522)	-24.1 (-33.0 to -12.0)	152 047 (135 693 to 173 545)	-35.0 (-43.2 to -25.0)	265 477 (228 943 to 308 013)	-13.9 (-22.3 to -4.1)
Georgia	408 962 (384 851 to 434 592)	-33.1 (-36.1 to -30.1)	1 248 448 (1 174 486 to 1 332 940)	-23.8 (-25.4 to -22.3)	47 631 (34 020 to 64 355)	-28.6 (-30.6 to -26.6)	2 266 (2 154 to 2 356)	-32.6 (-36.8 to -28.6)	87 808 (83 686 to 91 692)	-45.4 (-49.0 to -41.7)	135 439 (121 196 to 152 196)	-40.5 (-43.5 to -37.5)
Kazakhstan	2 075 610 (1 944 267 to 2 220 755)	4.6 (0.8 to 8.8)	5 112 179 (4 788 784 to 5 453 140)	13.9 (12.6 to 15.3)	196 345 (140 106 to 266 072)	1.6 (-2.8 to 5.9)	13 682 (12 933 to 14 551)	-16.9 (-21.5 to -11.9)	651 845 (613 935 to 700 301)	-25.6 (-30.2 to -20.1)	848 190 (777 257 to 930 185)	-20.7 (-25.1 to -16.1)
Kyrgyzstan	595 856 (551 142 to 647 311)	24.0 (18.0 to 29.9)	1 384 772 (1 295 005 to 1 483 197)	35.8 (33.9 to 37.6)	56 571 (40 845 to 75 906)	24.6 (21.0 to 28.4)	2 781 (2 656 to 2 943)	-30.8 (-35.3 to -25.4)	141 323 (134 314 to 149 712)	-37.0 (-41.6 to -31.8)	197 894 (181 209 to 217 900)	-26.6 (-31.6 to -21.4)
Mongolia	393 572 (367 277 to 424 055)	83.4 (76.7 to 91.1)	920 701 (862 500 to 985 058)	125.4 (122.1 to 128.9)	40 305 (29 218 to 53 761)	90.6 (81.4 to 101.0)	2 397 (2 145 to 2 678)	42.4 (24.5 to 60.5)	125 696 (112 101 to 140 700)	27.4 (9.7 to 45.1)	166 002 (147 447 to 186 179)	38.6 (22.9 to 53.9)
Tajikistan	892 670 (825 250 to 974 426)	77.5 (71.2 to 84.3)	2 194 161 (2 033 012 to 2 380 107)	116.3 (107.4 to 129.0)	98 536 (73 106 to 129 987)	96.1 (85.0 to 110.9)	3 173 (2 907 to 3 522)	10.2 (-0.1 to 22.7)	184 845 (168 186 to 206 986)	2.6 (-8.3 to 15.7)	283 381 (251 211 to 318 233)	23.0 (12.5 to 34.9)
Turkmenistan	507 194 (470 442 to 548 741)	49.2 (42.7 to 55.7)	1 221 787 (1 140 691 to 1 313 632)	74.9 (72.1 to 78.0)	48 814 (35 235 to 65 772)	44.4 (36.6 to 53.3)	1 767 (1 618 to 1 940)	-24.0 (-32.2 to -14.9)	93 954 (85 582 to 104 298)	-36.7 (-44.4 to -27.6)	142 768 (126 424 to 161 955)	-21.6 (-29.7 to -13.2)
Uzbekistan	3 149 923 (2 911 470 to 3 404 424)	65.3 (58.4 to 72.6)	7 308 189 (6 819 542 to 7 858 926)	94.0 (91.4 to 96.6)	293 056 (210 540 to 395 364)	70.5 (63.4 to 77.8)	12 931 (11 514 to 14 434)	7.2 (-5.5 to 21.1)	680 138 (605 843 to 759 676)	-7.9 (-19.2 to 3.9)	973 194 (858 111 to 1 098 708)	6.9 (-4.0 to 17.6)
**Central Europe**	**23 434 508 (21 840 770 to 25 160 864)**	**-5.3 (-9.1 to -1.4)**	**62 144 262 (58 671 497 to 65 823 195)**	**9.4 (8.5 to 10.3)**	**2 606 792 (1 862 314 to 3 561 320)**	**2.6 (-0.2 to 5.6)**	**60 287 (58 767 to 61 768)**	**-35.4 (-37.0 to -33.7)**	**1 926 268 (1 871 091 to 1 979 945)**	**-49.3 (-50.7 to -47.8)**	**4 533 060 (3 791 861 to 5 489 748)**	**-28.5 (-32.9 to -24.0)**
Albania	477 715 (441 096 to 519 444)	-8.9 (-13.5 to -3.9)	1 314 106 (1 229 740 to 1 403 210)	23.6 (20.9 to 26.2)	52 200 (37 145 to 71 490)	13.1 (7.1 to 18.8)	810 (677 to 964)	-37.0 (-47.4 to -24.9)	34 245 (28 729 to 40 710)	-53.1 (-61.1 to -44.2)	86 445 (69 892 to 105 803)	-27.5 (-35.7 to -19.8)
Bosnia and Herzegovina	592 536 (547 007 to 642 683)	-5.6 (-9.9 to -1.4)	1 933 300 (1 828 320 to 2 045 624)	24.3 (19.1 to 30.1)	78 639 (57 426 to 105 845)	14.7 (7.6 to 23.9)	1 144 (1 040 to 1 311)	-35.3 (-44.0 to -16.9)	38 446 (35 450 to 42 343)	-50.9 (-56.8 to -39.1)	117 085 (95 305 to 143 464)	-20.3 (-28.2 to -10.8)
Bulgaria	1 304 389 (1 212 516 to 1 400 235)	-18.7 (-22.7 to -14.2)	3 900 125 (3 677 529 to 4 146 917)	-10.6 (-11.5 to -9.6)	155 161 (109 830 to 213 028)	-15.7 (-18.1 to -13.3)	3 341 (3 148 to 3 536)	-42.2 (-45.5 to -38.9)	111 307 (104 360 to 118 630)	-53.4 (-56.3 to -50.5)	266 467 (220 024 to 324 258)	-37.0 (-40.7 to -33.4)
Croatia	803 693 (767 692 to 844 326)	-12.7 (-17.2 to -7.6)	2 037 209 (1 939 339 to 2 143 039)	-8.7 (-10.6 to -6.7)	86 584 (62 188 to 117 005)	-3.2 (-5.7 to -0.2)	2 702 (2 554 to 2 860)	-36.0 (-39.7 to -32.0)	64 083 (60 300 to 67 758)	-58.9 (-61.4 to -56.4)	150 667 (126 532 to 181 271)	-38.6 (-42.7 to -34.7)
Czech Republic	2 589 486 (2 398 804 to 2 815 495)	3.0 (-3.0 to 9.1)	6 590 749 (6 249 203 to 6 952 624)	26.2 (25.1 to 27.4)	289 282 (206 144 to 394 859)	23.9 (21.7 to 26.1)	5 458 (5 136 to 5 795)	-40.6 (-44.1 to -36.8)	154 607 (145 331 to 164 905)	-48.5 (-51.6 to -44.9)	443 889 (358 727 to 550 071)	-16.8 (-23.2 to -10.6)
Hungary	2 053 184 (1 896 046 to 2 231 410)	-19.5 (-24.3 to -14.2)	5 286 649 (4 976 182 to 5 624 106)	1.4 (0.2 to 3.0)	222 407 (158 996 to 305 153)	-9.7 (-12.2 to -7.2)	5 902 (5 608 to 6 215)	-54.5 (-56.8 to -51.9)	160 864 (152 326 to 170 230)	-62.1 (-64.3 to -59.9)	383 271 (317 903 to 465 447)	-42.9 (-47.5 to -38.4)
Macedonia	375 955 (345 979 to 407 098)	25.5 (20.3 to 30.7)	1 069 834 (997 359 to 1 145 729)	45.3 (42.8 to 47.6)	41 715 (29 610 to 57 251)	35.2 (29.8 to 40.4)	646 (599 to 693)	-15.3 (-23.3 to -7.9)	23 183 (21 649 to 24 925)	-34.6 (-40.4 to -29.1)	64 898 (52 434 to 80 349)	-2.1 (-10.0 to 5.2)
Montenegro	115 649 (107 177 to 124 653)	4.4 (0.3 to 8.7)	314 699 (294 836 to 335 448)	23.1 (21.5 to 24.6)	12 431 (8 836 to 17 054)	23.1 (20.4 to 25.6)	263 (241 to 287)	-13.9 (-21.7 to -4.7)	9 130 (8 328 to 10 015)	-35.1 (-41.3 to -28.1)	21 561 (17 830 to 26 198)	-10.8 (-17.5 to -4.8)
Poland	8 093 489 (7 529 622 to 8 723 151)	5.7 (-0.1 to 12.1)	20 621 336 (19 491 138 to 21 835 137)	19.6 (18.4 to 20.8)	884 230 (633 333 to 1 212 494)	12.6 (8.7 to 16.4)	21 676 (20 509 to 22 943)	-23.5 (-27.9 to -18.8)	722 212 (680 658 to 766 864)	-39.9 (-43.6 to -35.9)	1 606 442 (1 349 239 to 1 928 508)	-19.2 (-24.5 to -14.1)
Romania	3 744 708 (3 485 309 to 4 024 264)	-22.2 (-25.9 to -18.0)	10 344 791 (9 774 899 to 10 993 558)	-10.7 (-12.2 to -9.2)	422 170 (299 582 to 577 278)	-21.0 (-24.5 to -17.6)	10 147 (9 623 to 10 700)	-40.3 (-43.5 to -37.2)	359 365 (339 557 to 379 788)	-54.7 (-57.3 to -52.1)	781 535 (654 960 to 937 328)	-41.2 (-44.8 to -37.7)
Serbia	1 572 707 (1 457 066 to 1 701 110)	7.3 (3.1 to 11.7)	4 566 063 (4 282 324 to 4 876 556)	17.3 (15.7 to 19.1)	178 781 (127 225 to 245 019)	13.1 (9.9 to 16.5)	4 090 (3 850 to 4 356)	-29.4 (-34.0 to -24.2)	124 821 (116 949 to 134 432)	-50.1 (-53.7 to -45.8)	303 602 (251 964 to 370 181)	-25.6 (-31.3 to -19.6)
Slovakia	1 147 031 (1 066 912 to 1 237 008)	-0.5 (-4.7 to 4.2)	2 888 077 (2 724 802 to 3 060 063)	18.9 (17.9 to 19.9)	122 518 (87 339 to 167 745)	12.6 (10.3 to 15.0)	2 737 (2 526 to 2 950)	-33.3 (-38.0 to -27.2)	90 610 (83 999 to 98 030)	-44.7 (-48.5 to -39.6)	213 127 (176 719 to 259 666)	-21.8 (-26.9 to -16.2)
Slovenia	563 967 (519 321 to 614 167)	13.3 (6.4 to 21.0)	1 277 323 (1 216 516 to 1 340 578)	25.9 (24.7 to 27.2)	60 676 (43 468 to 82 624)	32.6 (30.5 to 34.7)	1 373 (1 278 to 1 466)	-25.1 (-30.3 to -19.9)	33 395 (30 959 to 35 738)	-51.5 (-55.1 to -48.1)	94 071 (76 652 to 115 708)	-17.9 (-24.9 to -11.6)
**Eastern Europe**	**33 990 447 (31 644 152 to 36 568 997)**	**-8.2 (-10.6 to -5.6)**	**101 667 841 (95 926 872 to 108 158 712)**	**4.4 (3.5 to 5.5)**	**3 820 990 (2 709 172 to 5 207 770)**	**-4.2 (-7.0 to -1.3)**	**212 132 (208 018 to 216 191)**	**-19.5 (-20.8 to -18.2)**	**8 666 550 (8 494 050 to 8 837 614)**	**-28.4 (-29.5 to -27.2)**	**12 487 540 (11 368 616 to 13 890 745)**	**-22.4 (-24.3 to -20.3)**
Belarus	1 534 236 (1 440 635 to 1 638 001)	-4.1 (-7.9 to -0.0)	4 602 984 (4 344 094 to 4 889 409)	6.1 (4.9 to 7.3)	172 736 (122 917 to 235 802)	-1.7 (-4.7 to 1.4)	8 038 (7 539 to 8 571)	-21.4 (-26.2 to -15.5)	297 698 (278 127 to 318 458)	-36.8 (-41.0 to -31.7)	470 434 (415 940 to 536 212)	-27.2 (-31.4 to -22.9)
Estonia	207 288 (194 165 to 222 276)	-30.3 (-33.7 to -26.8)	651 930 (615 947 to 693 092)	-11.9 (-13.2 to -10.6)	24 700 (17 564 to 33 767)	-20.5 (-22.2 to -18.8)	738 (648 to 835)	-64.8 (-69.2 to -60.0)	25 504 (22 211 to 29 151)	-72.5 (-76.1 to -68.4)	50 204 (42 223 to 59 457)	-59.4 (-63.5 to -55.0)
Latvia	323 600 (303 134 to 345 981)	-40.4 (-43.4 to -37.1)	978 734 (927 400 to 1 035 941)	-25.8 (-26.8 to -24.6)	37 574 (26 704 to 51 411)	-33.9 (-35.4 to -32.2)	1 605 (1 429 to 1 789)	-58.0 (-62.5 to -53.2)	55 765 (49 177 to 62 555)	-66.1 (-70.0 to -62.0)	93 339 (79 938 to 107 800)	-57.8 (-61.7 to -54.4)
Lithuania	519 584 (486 419 to 552 184)	-23.8 (-27.3 to -20.1)	1 492 072 (1 416 929 to 1 575 369)	-9.6 (-10.5 to -8.7)	58 930 (41 914 to 80 495)	-14.4 (-16.4 to -12.4)	2 937 (2 753 to 3 125)	-35.7 (-39.7 to -31.6)	101 852 (95 363 to 109 153)	-49.7 (-52.9 to -46.1)	160 781 (142 429 to 181 854)	-40.7 (-44.0 to -37.2)
Moldova	497 691 (465 583 to 534 512)	-27.8 (-31.0 to -24.1)	1 560 859 (1 458 531 to 1 675 495)	-7.7 (-9.1 to -5.9)	59 638 (42 569 to 80 762)	-18.0 (-21.1 to -14.9)	2 711 (2 615 to 2 818)	-40.5 (-43.1 to -37.8)	106 041 (101 971 to 110 616)	-53.6 (-56.0 to -50.9)	165 680 (148 631 to 185 664)	-45.1 (-47.9 to -42.0)
Russian Federation	24 070 449 (22 386 997 to 25 923 629)	-4.5 (-7.1 to -1.7)	70 793 750 (66 827 146 to 75 275 956)	8.5 (7.2 to 10.0)	2 672 932 (1 895 482 to 3 637 547)	-0.1 (-3.4 to 3.0)	153 835 (151 467 to 156 467)	-17.3 (-18.6 to -16.3)	6 281 437 (6 182 264 to 6 391 943)	-26.9 (-27.9 to -26.0)	8 954 368 (8 167 933 to 9 922 834)	-20.6 (-22.6 to -18.4)
Ukraine	6 837 600 (6 345 806 to 7 382 962)	-14.6 (-17.2 to -11.9)	21 587 513 (20 312 232 to 23 005 517)	-3.6 (-4.5 to -2.7)	794 481 (562 608 to 1 088 059)	-12.4 (-15.1 to -9.6)	42 269 (39 950 to 44 736)	-18.8 (-23.4 to -13.7)	1 798 254 (1 696 522 to 1 903 781)	-23.3 (-27.8 to -18.3)	2 592 735 (2 338 053 to 2 887 960)	-20.3 (-23.8 to -16.6)
**High-income**	**123 091 410 (116 452 763 to 130 455 209)**	**20.2 (16.7 to 24.2)**	**340 727 697 (322 370 944 to 364 666 316)**	**23.1 (21.6 to 24.8)**	**12 787 629 (9 187 273 to 17 416 431)**	**25.0 (23.2 to 26.9)**	**539 193 (530 194 to 549 750)**	**1.8 (0.0 to 3.8)**	**16 038 265 (15 777 572 to 16 313 027)**	**-25.0 (-26.2 to -23.6)**	**28 825 894 (25 251 254 to 33 410 715)**	**-8.8 (-12.4 to -5.2)**
**Australasia**	**6 372 069 (5 977 992 to 6 800 336)**	**78.2 (71.6 to 85.1)**	**14 255 586 (13 299 901 to 15 429 125)**	**67.6 (65.9 to 69.3)**	**558 803 (396 072 to 759 974)**	**80.8 (78.3 to 83.8)**	**11 607 (10 704 to 12 540)**	**19.4 (9.9 to 28.9)**	**353 521 (322 125 to 385 763)**	**-20.1 (-27.1 to -12.8)**	**912 325 (741 500 to 1 116 682)**	**21.4 (11.6 to 30.1)**
Australia	5 158 962 (4 827 068 to 5 521 165)	81.4 (74.1 to 89.3)	11 738 784 (10 928 611 to 12 733 490)	71.5 (69.9 to 73.1)	454 781 (322 109 to 619 896)	83.9 (81.0 to 87.1)	9 804 (8 903 to 10 743)	25.7 (14.2 to 37.5)	292 418 (261 634 to 324 251)	-16.4 (-25.3 to -7.4)	747 198 (606 534 to 915 179)	25.1 (14.8 to 34.3)
New Zealand	1 213 106 (1 147 017 to 1 288 795)	65.8 (58.7 to 72.8)	2 516 802 (2 368 331 to 2 697 053)	51.5 (48.1 to 54.7)	104 023 (73 957 to 141 253)	68.6 (65.2 to 72.3)	1 803 (1 714 to 1 904)	-6.3 (-11.1 to -0.9)	61 104 (57 986 to 64 665)	-34.0 (-37.5 to -30.1)	165 126 (134 702 to 202 927)	7.0 (-1.7 to 14.8)
**High-income Asia-Pacific**	**20 016 404 (18 787 457 to 21 337 120)**	**16.4 (12.8 to 20.6)**	**72 038 436 (67 846 095 to 77 770 580)**	**47.7 (46.0 to 49.7)**	**2 687 069 (1 930 196 to 3 636 987)**	**43.0 (40.0 to 46.1)**	**103 852 (100 396 to 107 498)**	**11.0 (7.1 to 14.9)**	**2 702 698 (2 604 387 to 2 812 831)**	**-30.5 (-33.1 to -27.6)**	**5 389 767 (4 647 372 to 6 347 818)**	**-6.5 (-12.1 to -1.3)**
Brunei	48 810 (45 660 to 52 336)	66.6 (60.9 to 72.2)	119 186 (111 585 to 128 042)	107.7 (104.7 to 110.9)	4 398 (3 096 to 5 997)	84.0 (76.6 to 91.6)	175 (160 to 191)	28.5 (16.4 to 42.4)	8 275 (7 551 to 9 102)	11.5 (0.4 to 24.1)	12 673 (11 131 to 14 435)	29.2 (18.9 to 40.2)
Japan	14 236 625 (13 278 704 to 15 215 511)	20.6 (16.3 to 25.3)	53 460 595 (50 382 784 to 57 752 684)	48.5 (46.7 to 50.5)	1 996 775 (1 436 508 to 2 691 759)	48.4 (46.0 to 51.0)	71 682 (69 548 to 73 862)	21.7 (18.3 to 25.3)	1 720 609 (1 668 002 to 1 785 987)	-19.5 (-21.9 to -16.5)	3 717 384 (3 159 300 to 4 412 096)	6.7 (1.3 to 11.7)
South Korea	5 175 653 (4 869 412 to 5 512 528)	2.7 (-2.3 to 8.2)	16 738 355 (15 727 612 to 17 989 997)	40.4 (37.6 to 43.5)	622 576 (442 634 to 853 947)	24.2 (18.9 to 30.4)	30 979 (28 621 to 33 260)	-7.3 (-14.8 to 0.2)	935 047 (862 491 to 1 006 393)	-44.7 (-49.4 to -40.0)	1 557 623 (1 369 867 to 1 797 853)	-29.0 (-34.2 to -23.4)
Singapore	555 316 (519 320 to 598 747)	74.3 (68.7 to 81.2)	1 720 300 (1 612 859 to 1 854 259)	120.5 (117.6 to 123.4)	63 320 (44 766 to 87 082)	112.9 (107.8 to 117.9)	1 016 (946 to 1 081)	-6.0 (-12.5 to -0.2)	38 767 (36 225 to 41 232)	-24.1 (-29.4 to -19.2)	102 087 (83 489 to 125 178)	26.3 (14.9 to 37.5)
**High-income North America**	**46 036 101 (43 002 682 to 49 180 391)**	**18.4 (12.7 to 24.8)**	**102 679 176 (97 041 251 to 109 946 485)**	**3.3 (0.1 to 6.0)**	**3 843 166 (2 773 221 to 5 217 049)**	**7.7 (4.2 to 10.8)**	**203 286 (198 415 to 207 809)**	**21.2 (18.1 to 24.0)**	**7 129 107 (6 928 436 to 7 314 235)**	**-6.2 (-9.0 to -3.6)**	**10 972 274 (9 878 836 to 12 358 996)**	**-1.8 (-4.3 to 0.5)**
Canada	3 965 165 (3 739 035 to 4 206 290)	44.4 (39.7 to 49.8)	10 874 536 (10 305 635 to 11 660 226)	56.5 (54.5 to 58.4)	405 253 (288 734 to 552 917)	59.5 (57.1 to 62.1)	16 818 (15 914 to 17 751)	23.0 (16.7 to 29.9)	487 872 (460 410 to 516 224)	-17.0 (-21.8 to -12.0)	893 125 (775 732 to 1 045 577)	6.1 (-0.1 to 12.2)
Greenland	6 426 (6 064 to 6 799)	-25.4 (-28.4 to -22.3)	15 845 (14 933 to 16 999)	-19.7 (-21.5 to -18.1)	611 (439 to 830)	-26.2 (-29.4 to -23.3)	63 (59 to 68)	-47.0 (-51.3 to -41.6)	2 603 (2 424 to 2 794)	-60.3 (-63.5 to -56.5)	3 215 (2 971 to 3 483)	-56.4 (-59.6 to -52.9)
USA	42 063 704 (39 207 538 to 45 021 385)	16.4 (10.5 to 23.2)	91 786 998 (86 616 917 to 98 326 910)	-0.7 (-4.0 to 2.1)	3 437 234 (2 480 352 to 4 661 861)	3.7 (0.2 to 6.9)	186 401 (181 743 to 190 790)	21.1 (17.9 to 24.2)	6 638 507 (6 443 632 to 6 824 718)	-5.3 (-8.2 to -2.5)	10 075 742 (9 106 576 to 11 315 171)	-2.4 (-4.8 to -0.1)
**Southern Latin America**	**6 415 216 (6 084 735 to 6 764 067)**	**51.2 (46.5 to 55.9)**	**16 739 414 (15 827 753 to 17 832 407)**	**61.0 (58.9 to 63.4)**	**611 194 (436 691 to 830 058)**	**42.2 (35.7 to 48.8)**	**33 493 (31 102 to 36 245)**	**9.4 (1.1 to 19.2)**	**1 373 512 (1 269 361 to 1 498 579)**	**-4.8 (-12.5 to 4.1)**	**1 984 706 (1 769 822 to 2 233 843)**	**6.0 (-1.1 to 14.1)**
Argentina	4 409 379 (4 149 367 to 4 671 584)	57.7 (52.7 to 62.8)	11 389 746 (10 745 989 to 12 163 066)	62.9 (60.7 to 65.1)	422 128 (301 032 to 572 709)	44.9 (38.3 to 51.6)	22 657 (20 377 to 25 309)	21.1 (8.8 to 35.3)	963 464 (860 834 to 1 079 128)	11.4 (-0.5 to 25.3)	1 385 591 (1 223 174 to 1 573 483)	19.8 (10.3 to 30.8)
Chile	1 636 217 (1 566 915 to 1 709 722)	42.5 (35.4 to 50.0)	4 349 664 (4 128 493 to 4 603 676)	65.8 (62.0 to 69.5)	152 995 (108 072 to 208 027)	41.3 (33.9 to 49.2)	8 329 (7 420 to 9 372)	-14.8 (-24.2 to -4.1)	319 978 (282 402 to 361 931)	-34.2 (-41.9 to -25.2)	472 972 (412 258 to 543 988)	-20.5 (-28.3 to -12.2)
Uruguay	369 337 (348 182 to 391 509)	23.2 (18.6 to 28.3)	999 263 (947 303 to 1 066 121)	28.5 (26.8 to 30.3)	36 045 (25 631 to 49 156)	19.3 (15.0 to 23.2)	2 506 (2 265 to 2 756)	17.7 (5.7 to 29.5)	90 010 (80 306 to 100 109)	-0.4 (-11.6 to 10.9)	126 055 (112 373 to 142 451)	4.5 (-4.2 to 13.1)
**Western Europe**	**44 251 621 (41 744 078 to 47 130 756)**	**15.0 (11.6 to 18.9)**	**135 015 086 (127 409 844 to 144 506 511)**	**23.2 (22.0 to 24.5)**	**5 087 396 (3 625 663 to 6 949 633)**	**25.8 (24.6 to 27.2)**	**186 956 (181 467 to 193 359)**	**-18.0 (-20.7 to -14.9)**	**4 479 427 (4 346 276 to 4 627 949)**	**-44.1 (-45.9 to -42.2)**	**9 566 823 (8 117 967 to 11 417 777)**	**-20.6 (-25.8 to -15.6)**
Andorra	8 198 (7 673 to 8 787)	58.2 (52.4 to 65.2)	25 907 (24 376 to 27 823)	75.2 (72.2 to 78.3)	975 (693 to 1 335)	82.8 (79.5 to 86.1)	26 (23 to 30)	32.4 (8.9 to 61.3)	760 (652 to 880)	-13.1 (-30.3 to 8.6)	1 735 (1 424 to 2 103)	23.2 (8.1 to 41.1)
Austria	927 989 (872 343 to 991 670)	3.4 (-1.7 to 8.5)	2 848 963 (2 689 849 to 3 056 157)	15.7 (14.3 to 17.1)	107 760 (76 620 to 147 538)	16.6 (14.7 to 18.2)	4 161 (3 920 to 4 407)	-26.6 (-31.0 to -22.3)	105 906 (99 293 to 113 169)	-46.8 (-50.1 to -43.3)	213 667 (180 477 to 252 204)	-26.7 (-31.8 to -21.7)
Belgium	1 396 148 (1 311 165 to 1 495 855)	34.4 (28.7 to 40.9)	3 936 058 (3 704 648 to 4 215 284)	35.2 (33.6 to 36.8)	150 630 (106 891 to 205 689)	36.7 (34.3 to 39.2)	7 062 (6 684 to 7 440)	0.6 (-5.2 to 6.9)	177 564 (167 079 to 188 155)	-29.5 (-33.8 to -25.4)	328 194 (282 686 to 386 576)	-9.4 (-14.8 to -3.9)
Cyprus	122 132 (114 649 to 130 545)	57.6 (52.1 to 63.4)	372 963 (350 535 to 398 167)	85.0 (82.5 to 87.5)	13 879 (9 848 to 19 015)	75.1 (70.9 to 79.4)	443 (399 to 489)	4.0 (-6.7 to 15.6)	14 717 (13 340 to 16 291)	-11.4 (-20.7 to -1.9)	28 596 (24 299 to 33 920)	16.6 (6.8 to 25.7)
Denmark	581 282 (542 641 to 623 517)	1.0 (-3.5 to 5.7)	1 755 293 (1 652 671 to 1 882 786)	17.5 (16.1 to 19.0)	65 473 (46 587 to 89 655)	15.0 (13.2 to 16.9)	2 289 (2 153 to 2 427)	-46.2 (-49.4 to -42.7)	54 189 (50 545 to 57 904)	-57.6 (-60.5 to -54.7)	119 662 (100 056 to 144 037)	-35.3 (-40.8 to -30.0)
Finland	701 768 (659 057 to 751 540)	19.3 (14.0 to 25.2)	2 056 128 (1 944 202 to 2 197 755)	31.5 (30.1 to 32.8)	78 636 (55 827 to 107 681)	34.9 (32.9 to 37.0)	3 145 (2 975 to 3 349)	-28.0 (-32.3 to -23.0)	81 507 (76 628 to 87 580)	-51.2 (-54.2 to -47.4)	160 143 (136 670 to 190 189)	-28.9 (-34.6 to -23.0)
France	7 524 271 (7 072 177 to 8 046 169)	11.6 (7.2 to 16.2)	21 503 312 (20 323 765 to 22 973 967)	21.1 (19.6 to 22.7)	825 139 (587 837 to 1 126 031)	24.3 (22.5 to 26.3)	40 603 (38 325 to 43 190)	-22.7 (-27.3 to -17.2)	916 538 (859 895 to 975 173)	-45.0 (-48.6 to -41.5)	1 741 677 (1 487 063 to 2 030 298)	-25.3 (-31.1 to -19.8)
Germany	8 814 380 (8 273 028 to 9 424 964)	13.6 (8.5 to 19.4)	27 313 087 (25 756 767 to 29 281 440)	20.9 (19.4 to 22.3)	1 032 325 (731 559 to 1 416 369)	22.4 (20.5 to 24.3)	38 763 (35 221 to 42 648)	-16.4 (-23.9 to -8.4)	923 573 (829 220 to 1 025 616)	-42.3 (-48.0 to -36.0)	1 955 898 (1 638 299 to 2 333 986)	-20.0 (-26.1 to -13.9)
Greece	990 407 (931 647 to 1 055 065)	0.1 (-3.5 to 3.7)	3 364 182 (3 171 733 to 3 602 733)	14.9 (13.5 to 16.3)	125 163 (88 836 to 171 636)	15.1 (13.4 to 17.1)	3 792 (3 592 to 4 007)	-20.8 (-25.3 to -15.3)	109 967 (103 936 to 116 642)	-40.2 (-43.7 to -36.6)	235 131 (196 593 to 282 209)	-19.7 (-24.6 to -14.7)
Iceland	33 859 (31 834 to 36 311)	38.3 (34.1 to 42.9)	96 606 (90 822 to 103 590)	57.9 (55.9 to 59.9)	3 621 (2 574 to 4 941)	58.4 (56.1 to 61.0)	120 (115 to 125)	-0.9 (-6.1 to 4.9)	3 477 (3 321 to 3 657)	-27.7 (-31.7 to -23.2)	7 099 (6 011 to 8 441)	0.0 (-7.1 to 7.2)
Ireland	462 640 (433 993 to 496 764)	39.4 (34.9 to 44.4)	1 346 044 (1 265 093 to 1 441 900)	60.3 (57.9 to 62.9)	49 664 (35 196 to 67 615)	62.8 (60.1 to 65.9)	1 239 (1 154 to 1 325)	-17.2 (-22.9 to -11.2)	41 236 (38 177 to 44 342)	-33.2 (-38.1 to -28.0)	90 900 (75 990 to 109 005)	-1.4 (-9.6 to 5.9)
Israel	847 529 (787 568 to 918 288)	78.1 (72.4 to 83.7)	2 230 178 (2 079 425 to 2 398 128)	113.3 (108.6 to 118.8)	83 717 (59 796 to 113 434)	110.8 (105.4 to 119.1)	2 351 (2 210 to 2 521)	20.7 (12.9 to 38.0)	75 063 (70 616 to 80 196)	-7.2 (-13.4 to 2.2)	158 780 (134 120 to 188 266)	31.7 (22.3 to 41.9)
Italy	5 683 666 (5 337 468 to 6 068 440)	3.1 (-1.6 to 8.2)	18 220 081 (17 270 169 to 19 425 065)	6.5 (4.9 to 8.2)	690 970 (491 286 to 940 224)	13.4 (11.3 to 15.6)	23 641 (22 286 to 24 977)	-21.1 (-25.8 to -16.7)	494 985 (463 372 to 526 727)	-50.6 (-53.7 to -47.3)	1 185 955 (987 717 to 1 437 380)	-26.4 (-31.5 to -21.1)
Luxembourg	64 254 (60 415 to 68 731)	34.0 (29.1 to 39.3)	188 345 (177 873 to 201 181)	43.5 (41.7 to 45.0)	7 057 (5 031 to 9 585)	45.5 (43.3 to 47.9)	246 (224 to 270)	-7.1 (-16.1 to 2.0)	6 425 (5 781 to 7 092)	-35.5 (-42.3 to -28.5)	13 482 (11 324 to 15 978)	-9.0 (-16.3 to -1.9)
Malta	45 355 (42 674 to 48 439)	27.0 (21.3 to 32.8)	144 195 (135 607 to 154 725)	53.3 (51.0 to 55.4)	5 394 (3 832 to 7 357)	58.3 (55.6 to 61.2)	139 (133 to 147)	24.8 (17.4 to 32.7)	3 592 (3 423 to 3 782)	-10.8 (-16.3 to -4.7)	8 986 (7 362 to 10 951)	20.9 (13.9 to 27.3)
Netherlands	1 523 044 (1 443 917 to 1 610 301)	23.9 (18.2 to 30.0)	4 403 697 (4 164 113 to 4 691 713)	31.5 (29.1 to 33.8)	166 419 (118 071 to 227 398)	34.0 (31.4 to 36.7)	7 531 (7 117 to 8 002)	20.1 (13.3 to 27.5)	163 851 (155 034 to 173 951)	-24.7 (-28.8 to -19.9)	330 270 (279 438 to 391 229)	-3.4 (-9.2 to 1.7)
Norway	718 010 (670 410 to 766 148)	43.5 (39.8 to 47.3)	1 725 196 (1 621 160 to 1 862 002)	30.1 (28.8 to 31.3)	69 043 (49 868 to 92 702)	35.3 (33.6 to 37.0)	2 357 (2 286 to 2 433)	-17.1 (-19.9 to -14.2)	56 657 (55 005 to 58 651)	-40.9 (-43.0 to -38.6)	125 700 (106 647 to 149 089)	-14.5 (-19.9 to -9.3)
Portugal	907 222 (853 246 to 965 626)	-11.4 (-15.5 to -7.2)	2 983 597 (2 824 008 to 3 184 063)	-2.1 (-4.3 to 0.0)	110 002 (77 722 to 150 051)	-5.5 (-8.3 to -2.8)	4 510 (4 236 to 4 780)	-41.7 (-45.2 to -38.1)	117 277 (109 383 to 125 230)	-64.3 (-66.8 to -61.7)	227 279 (192 888 to 266 162)	-48.9 (-53.3 to -44.8)
Spain	4 341 783 (4 072 780 to 4 628 409)	15.5 (11.0 to 20.5)	14 260 875 (13 454 595 to 15 240 479)	31.1 (29.2 to 33.1)	532 623 (378 328 to 728 924)	34.8 (32.7 to 37.1)	14 847 (13 975 to 15 835)	-24.4 (-29.0 to -19.0)	364 677 (341 900 to 387 912)	-55.7 (-58.6 to -52.7)	897 300 (739 098 to 1 089 079)	-26.4 (-33.3 to -19.8)
Sweden	1 115 967 (1 037 680 to 1 196 947)	33.8 (30.2 to 37.7)	3 334 969 (3 128 706 to 3 600 066)	31.8 (30.6 to 33.1)	127 253 (91 822 to 171 470)	34.2 (32.2 to 36.1)	4 607 (4 386 to 4 845)	-8.4 (-12.9 to -3.7)	109 751 (104 155 to 115 592)	-33.1 (-36.7 to -29.5)	237 004 (200 610 to 281 012)	-8.5 (-14.2 to -3.2)
Switzerland	976 761 (925 879 to 1 036 126)	-1.3 (-5.9 to 4.0)	2 772 915 (2 621 873 to 2 967 787)	10.2 (8.0 to 12.5)	105 959 (75 454 to 144 680)	9.3 (7.5 to 11.2)	3 888 (3 634 to 4 142)	-28.9 (-33.9 to -23.8)	84 072 (78 446 to 90 139)	-53.3 (-56.7 to -49.7)	190 031 (158 279 to 229 129)	-31.4 (-36.7 to -26.4)
United Kingdom	6 419 103 (5 983 585 to 6 883 619)	28.5 (25.3 to 31.8)	19 992 594 (18 786 018 to 21 452 027)	36.3 (34.9 to 38.0)	730 423 (519 131 to 997 648)	37.7 (36.5 to 39.2)	21 001 (20 677 to 21 308)	-2.7 (-4.4 to -1.3)	569 000 (560 459 to 578 416)	-29.9 (-31.1 to -28.7)	1 299 422 (1 086 575 to 1 568 530)	-3.2 (-8.7 to 2.0)
**Latin America and Caribbean**	**26 652 810 (25 179 699 to 28 336 753)**	**73.7 (69.2 to 78.6)**	**75 234 240 (71 347 469 to 79 651 998)**	**97.6 (93.3 to 102.3)**	**2 869 680 (2 125 013 to 3 776 141)**	**88.0 (79.1 to 96.0)**	**429 848 (421 659 to 438 254)**	**24.3 (21.5 to 27.0)**	**20 834 725 (20 432 386 to 21 266 267)**	**6.4 (3.8 to 9.1)**	**23 704 404 (22 817 409 to 24 649 534)**	**12.3 (9.1 to 15.6)**
**Andean Latin America**	**2 652 747 (2 508 770 to 2 814 213)**	**55.2 (49.1 to 60.5)**	**6 932 141 (6 570 562 to 7 334 805)**	**92.5 (88.1 to 97.0)**	**263 607 (194 050 to 348 009)**	**53.3 (40.7 to 66.5)**	**31 486 (28 869 to 34 277)**	**-10.5 (-19.5 to -1.0)**	**1 519 477 (1 379 441 to 1 663 045)**	**-32.4 (-40.4 to -23.3)**	**1 783 084 (1 620 469 to 1 937 608)**	**-26.3 (-34.1 to -17.4)**
Bolivia	456 690 (430 038 to 485 590)	94.6 (88.4 to 100.6)	1 137 599 (1 081 566 to 1 201 437)	112.2 (108.3 to 116.2)	45 285 (33 240 to 59 523)	77.7 (67.1 to 89.7)	6 187 (4 644 to 7 381)	-15.2 (-38.1 to 7.3)	299 043 (212 543 to 357 977)	-34.9 (-56.0 to -14.3)	344 327 (255 120 to 407 026)	-29.0 (-49.2 to -9.0)
Ecuador	773 461 (731 675 to 819 345)	105.5 (98.9 to 113.3)	2 024 578 (1 921 247 to 2 141 028)	111.3 (106.0 to 116.6)	75 992 (56 215 to 100 129)	66.9 (53.2 to 81.8)	11 855 (10 760 to 13 141)	65.6 (49.8 to 84.2)	576 291 (521 239 to 638 881)	47.1 (32.5 to 64.1)	652 283 (595 752 to 718 773)	49.2 (36.0 to 64.3)
Peru	1 422 596 (1 344 535 to 1 513 731)	29.5 (22.7 to 35.8)	3 769 964 (3 559 606 to 4 017 749)	79.0 (73.5 to 84.4)	142 331 (104 759 to 187 347)	41.0 (27.6 to 54.9)	13 443 (11 563 to 15 489)	-35.1 (-45.3 to -23.3)	644 143 (547 623 to 747 953)	-53.8 (-61.9 to -44.9)	786 474 (681 076 to 901 016)	-47.4 (-55.4 to -38.4)
**Caribbean**	**2 372 385 (2 250 175 to 2 504 083)**	**82.3 (78.1 to 86.8)**	**8 186 542 (7 636 650 to 8 776 986)**	**148.4 (135.0 to 164.7)**	**340 141 (253 308 to 440 630)**	**157.5 (132.8 to 190.5)**	**38 018 (35 426 to 40 864)**	**25.7 (16.5 to 37.6)**	**1 693 208 (1 566 733 to 1 833 814)**	**2.7 (-6.7 to 15.8)**	**2 033 349 (1 878 035 to 2 197 980)**	**14.2 (4.5 to 27.6)**
Antigua and Barbuda	4 400 (4 135 to 4 670)	89.1 (82.2 to 96.2)	12 260 (11 668 to 12 917)	104.7 (100.8 to 108.9)	402 (290 to 540)	86.4 (78.0 to 94.9)	35 (32 to 37)	30.1 (19.1 to 41.5)	1 507 (1 394 to 1 622)	23.4 (12.0 to 35.5)	1 909 (1 751 to 2 099)	32.9 (22.3 to 43.7)
The Bahamas	16 419 (15 490 to 17 437)	86.9 (81.4 to 92.8)	47 170 (44 955 to 49 652)	80.0 (73.8 to 85.9)	1 606 (1 170 to 2 145)	83.4 (74.8 to 91.4)	273 (249 to 298)	55.8 (41.7 to 71.7)	13 160 (12 005 to 14 390)	43.0 (29.2 to 58.2)	14 767 (13 512 to 16 067)	46.5 (33.8 to 60.4)
Barbados	13 657 (12 848 to 14 513)	55.0 (50.1 to 60.2)	41 180 (39 297 to 43 399)	63.4 (59.6 to 67.0)	1 403 (1 014 to 1 888)	59.5 (52.6 to 65.8)	132 (122 to 144)	3.3 (-5.2 to 12.2)	4 886 (4 466 to 5 318)	-11.6 (-19.7 to -3.4)	6 290 (5 708 to 6 952)	-1.9 (-9.2 to 5.9)
Belize	17 927 (16 928 to 19 033)	180.7 (172.6 to 189.4)	46 772 (44 365 to 49 436)	228.3 (219.6 to 237.5)	1 726 (1 287 to 2 274)	210.4 (197.3 to 223.5)	307 (292 to 321)	144.5 (127.0 to 163.2)	15 879 (15 088 to 16 717)	114.3 (97.0 to 133.3)	17 606 (16 643 to 18 513)	121.0 (104.4 to 138.8)
Bermuda	3 430 (3 215 to 3 654)	54.5 (49.3 to 59.9)	11 854 (11 298 to 12 471)	40.8 (35.9 to 45.4)	386 (279 to 517)	48.6 (39.0 to 56.6)	22 (20 to 24)	-28.1 (-33.8 to -21.8)	680 (616 to 747)	-49.4 (-54.6 to -43.7)	1 065 (933 to 1 213)	-33.5 (-39.9 to -26.9)
Cuba	677 332 (639 116 to 719 397)	52.2 (46.5 to 58.9)	1 768 711 (1 684 590 to 1 871 321)	57.5 (53.7 to 61.0)	61 811 (44 051 to 84 136)	50.7 (44.6 to 57.0)	7 737 (7 029 to 8 553)	-11.0 (-19.1 to -1.4)	210 010 (188 147 to 235 679)	-44.7 (-50.4 to -38.0)	271 821 (243 830 to 302 014)	-35.4 (-41.4 to -29.2)
Dominica	3 058 (2 894 to 3 240)	28.4 (24.7 to 32.4)	9 237 (8 797 to 9 724)	37.0 (33.4 to 40.6)	330 (241 to 438)	35.7 (29.9 to 41.4)	41 (39 to 44)	1.3 (-6.0 to 9.7)	1 828 (1 700 to 1 977)	-5.8 (-13.6 to 2.6)	2 159 (2 003 to 2 328)	-1.1 (-8.2 to 6.3)
Dominican Republic	515 165 (488 101 to 545 005)	116.7 (110.9 to 123.1)	1 279 296 (1 221 180 to 1 346 235)	132.8 (125.7 to 139.6)	47 209 (34 366 to 63 282)	91.8 (77.0 to 108.7)	8 388 (7 194 to 9 631)	89.5 (54.1 to 125.7)	405 798 (349 231 to 464 457)	52.4 (23.6 to 81.5)	453 008 (395 909 to 512 585)	55.8 (28.7 to 82.5)
Grenada	5 322 (5 040 to 5 628)	70.9 (64.8 to 76.9)	15 303 (14 595 to 16 125)	86.0 (80.8 to 90.7)	555 (405 to 739)	80.6 (71.5 to 89.0)	59 (55 to 62)	12.8 (4.9 to 21.7)	2 162 (2 026 to 2 307)	-11.2 (-19.0 to -2.7)	2 717 (2 505 to 2 940)	-0.9 (-9.2 to 7.2)
Guyana	32 779 (30 982 to 34 633)	26.7 (23.3 to 30.3)	83 890 (79 927 to 88 696)	27.9 (24.1 to 31.7)	3 316 (2 434 to 4 363)	24.5 (18.3 to 31.2)	671 (593 to 757)	7.8 (-5.1 to 22.5)	32 013 (28 224 to 36 052)	-5.2 (-16.8 to 7.9)	35 329 (31 709 to 39 296)	-3.0 (-13.8 to 9.0)
Haiti	415 206 (390 755 to 442 565)	95.2 (89.4 to 100.5)	3 266 023 (2 838 740 to 3 742 240)	445.0 (377.9 to 527.6)	161 388 (117 540 to 215 755)	471.6 (361.4 to 616.1)	11 068 (9 185 to 13 398)	6.1 (-13.7 to 38.5)	612 497 (511 484 to 734 359)	-7.9 (-25.4 to 25.2)	773 884 (662 543 to 905 598)	11.7 (-8.7 to 48.0)
Jamaica	138 230 (130 465 to 146 327)	60.2 (54.9 to 65.6)	370 303 (352 930 to 390 780)	82.6 (78.8 to 86.4)	13 115 (9 484 to 17 566)	72.6 (64.6 to 80.5)	1 664 (1 410 to 1 946)	166.7 (122.8 to 214.1)	77 838 (65 429 to 91 227)	146.9 (104.9 to 194.0)	90 953 (77 802 to 105 081)	132.5 (98.6 to 170.0)
Puerto Rico	333 914 (311 878 to 360 033)	132.4 (118.3 to 147.5)	628 487 (599 132 to 664 004)	66.8 (63.0 to 70.5)	23 192 (16 522 to 31 092)	74.1 (68.3 to 79.7)	4 580 (4 447 to 4 729)	79.8 (73.6 to 86.2)	179 565 (173 811 to 185 604)	50.5 (44.8 to 56.4)	202 758 (193 992 to 212 203)	52.8 (47.7 to 58.2)
Saint Lucia	7 801 (7 384 to 8 265)	70.4 (64.3 to 76.4)	23 121 (22 052 to 24 331)	88.1 (82.0 to 93.8)	808 (589 to 1 077)	79.9 (69.5 to 89.8)	110 (103 to 118)	34.1 (24.1 to 44.9)	4 810 (4 468 to 5 150)	15.9 (6.5 to 26.0)	5 618 (5 240 to 6 029)	22.2 (13.5 to 31.7)
Saint Vincent and the Grenadines	5 443 (5 161 to 5 744)	52.7 (47.2 to 58.4)	15 361 (14 650 to 16 200)	75.5 (70.0 to 81.0)	572 (420 to 756)	73.3 (65.8 to 80.8)	77 (72 to 82)	35.4 (25.4 to 45.5)	3 375 (3 166 to 3 584)	16.2 (6.6 to 26.0)	3 947 (3 684 to 4 236)	22.0 (13.2 to 31.2)
Suriname	25 968 (24 562 to 27 467)	96.1 (90.9 to 101.8)	72 041 (68 640 to 76 057)	98.6 (92.9 to 103.7)	2 768 (2 037 to 3 650)	76.8 (67.7 to 87.0)	455 (406 to 505)	37.2 (20.7 to 55.2)	20 082 (17 883 to 22 429)	13.5 (-0.3 to 29.0)	22 849 (20 594 to 25 288)	18.7 (5.7 to 33.3)

Location	Incidence (95% UI)		Prevalence (95% UI)		Years lived with disability (95% UI)		Deaths (95% UI)		Years of life lost (95% UI)		Disability adjusted life years (95% UI)	
	2017 all-age counts	Percentage change in age-standardised rates between 1990 and 2017	2017 all-age counts	Percentage change in age-standardised rates between 1990 and 2017	2017 all-age counts	Percentage change in age-standardised rates between 1990 and 2017	2017 all-age counts	Percentage change in age-standardised rates between 1990 and 2017	2017 all-age counts	Percentage change in age-standardised rates between 1990 and 2017	2017 all-age counts	Percentage change in age-standardised rates between 1990 and 2017
Trinidad and Tobago	65 018 (61 322 to 69 231)	41.0 (35.3 to 46.8)	184 710 (176 257 to 194 589)	78.5 (73.9 to 83.8)	6 760 (4 892 to 9 026)	66.7 (58.4 to 75.3)	942 (762 to 1 143)	26.5 (3.3 to 53.4)	43 080 (34 781 to 52 554)	10.7 (-10.4 to 34.7)	49 840 (41 167 to 59 196)	15.9 (-3.5 to 37.9)
Virgin Islands	6 003 (5 678 to 6 351)	52.0 (46.8 to 57.9)	16 432 (15 705 to 17 307)	59.5 (55.2 to 63.6)	562 (400 to 763)	45.7 (37.1 to 55.1)	90 (80 to 100)	27.2 (10.9 to 44.3)	3 148 (2 843 to 3 523)	-5.8 (-16.3 to 6.7)	3 710 (3 370 to 4 147)	-0.5 (-10.2 to 11.9)
**Central Latin America**	**10 588 764 (9 928 332 to 11 299 964)**	**64.3 (59.9 to 69.2)**	**29 201 889 (27 686 473 to 31 044 721)**	**73.8 (70.1 to 77.7)**	**1 062 300 (788 629 to 1 386 551)**	**58.6 (48.3 to 67.5)**	**190 158 (183 372 to 197 197)**	**29.1 (24.2 to 34.0)**	**9 559 607 (9 192 318 to 9 933 672)**	**15.3 (10.7 to 19.9)**	**10 621 908 (10 131 626 to 11 109 649)**	**18.5 (13.9 to 22.8)**
Colombia	1 862 767 (1 758 603 to 1 974 713)	36.2 (31.2 to 41.6)	5 463 986 (5 138 568 to 5 839 533)	63.0 (58.4 to 68.0)	196 487 (145 942 to 256 256)	35.6 (25.7 to 45.5)	31 595 (27 863 to 35 490)	-25.8 (-34.5 to -16.5)	1 604 994 (1 418 859 to 1 803 512)	-34.2 (-41.9 to -25.7)	1 801 481 (1 602 251 to 2 006 308)	-30.3 (-38.0 to -22.3)
Costa Rica	190 147 (178 991 to 201 801)	101.4 (94.1 to 109.3)	479 810 (455 995 to 506 420)	126.8 (121.4 to 132.2)	16 623 (11 919 to 22 252)	114.2 (103.9 to 124.5)	2 537 (2 367 to 2 709)	78.1 (65.5 to 91.8)	105 256 (97 999 to 112 719)	54.3 (42.7 to 66.9)	121 879 (113 573 to 131 217)	60.4 (50.2 to 71.7)
El Salvador	233 103 (219 927 to 247 811)	-14.1 (-21.6 to -7.4)	867 250 (755 788 to 1 017 245)	-6.2 (-13.5 to 2.4)	32 716 (24 279 to 42 227)	-36.0 (-46.2 to -24.8)	6 547 (5 378 to 7 741)	-8.7 (-25.1 to 8.0)	313 987 (256 210 to 374 559)	-22.3 (-36.9 to -7.5)	346 703 (289 287 to 408 014)	-23.9 (-37.0 to -10.5)
Guatemala	621 196 (584 123 to 660 217)	88.1 (78.1 to 97.5)	1 617 509 (1 505 791 to 1 768 291)	88.3 (74.3 to 102.7)	64 979 (48 536 to 84 377)	42.9 (22.9 to 62.9)	14 404 (12 900 to 16 093)	73.0 (55.2 to 93.9)	761 537 (681 839 to 853 156)	56.8 (40.2 to 76.4)	826 515 (744 856 to 921 199)	55.6 (40.3 to 73.9)
Honduras	319 373 (299 249 to 340 981)	124.1 (116.7 to 131.0)	937 591 (866 236 to 1 022 722)	208.8 (189.7 to 233.1)	38 395 (28 962 to 49 229)	165.2 (145.3 to 189.0)	7 040 (5 672 to 8 500)	52.4 (17.2 to 92.2)	360 983 (291 886 to 433 332)	32.5 (0.5 to 66.5)	399 377 (327 082 to 474 172)	39.2 (7.7 to 72.3)
Mexico	5 595 681 (5 173 125 to 6 060 678)	67.1 (61.0 to 72.9)	14 725 513 (13 904 370 to 15 675 094)	69.1 (65.0 to 73.1)	534 698 (394 445 to 707 014)	75.8 (69.5 to 81.3)	93 569 (92 105 to 94 983)	43.6 (40.7 to 46.2)	4 582 254 (4 520 073 to 4 649 039)	28.1 (25.5 to 31.2)	5 116 952 (4 954 436 to 5 299 532)	31.8 (29.0 to 34.9)
Nicaragua	205 421 (192 137 to 219 536)	76.3 (70.0 to 83.2)	771 186 (669 763 to 910 778)	25.9 (14.9 to 39.3)	29 476 (21 620 to 38 546)	-6.2 (-21.0 to 10.0)	2 270 (1 994 to 2 638)	-5.2 (-17.7 to 9.8)	110 577 (97 354 to 129 453)	-25.4 (-35.2 to -13.1)	140 053 (124 218 to 159 093)	-22.0 (-30.8 to -11.6)
Panama	148 813 (140 010 to 157 942)	104.2 (97.6 to 110.9)	390 158 (370 956 to 412 329)	118.5 (113.7 to 123.3)	13 677 (9 950 to 18 116)	90.7 (77.9 to 103.1)	1 868 (1 724 to 2 011)	37.0 (26.2 to 47.9)	91 145 (83 491 to 98 872)	22.5 (12.0 to 33.4)	104 822 (96 413 to 113 398)	28.5 (18.3 to 38.7)
Venezuela	1 412 263 (1 331 389 to 1 497 330)	101.8 (94.2 to 110.0)	3 948 885 (3 722 388 to 4 190 284)	140.2 (131.5 to 151.2)	135 248 (99 656 to 178 212)	113.5 (100.8 to 127.0)	30 327 (25 563 to 35 421)	112.3 (79.1 to 148.7)	1 628 876 (1 372 551 to 1 911 537)	98.2 (67.5 to 132.3)	1 764 124 (1 513 369 to 2 043 193)	99.3 (70.6 to 130.6)
**Tropical Latin America**	**11 038 914 (10 388 760 to 11 762 491)**	**87.5 (79.8 to 94.8)**	**30 913 669 (29 098 666 to 32 907 157)**	**115.1 (109.0 to 122.1)**	**1 203 631 (882 820 to 1 587 716)**	**117.8 (111.0 to 124.6)**	**170 186 (167 848 to 172 599)**	**27.8 (24.9 to 31.8)**	**8 062 432 (7 948 792 to 8 174 662)**	**9.1 (6.2 to 13.2)**	**9 266 063 (8 919 727 to 9 646 646)**	**16.6 (12.9 to 21.2)**
Brazil	10 726 143 (10 094 819 to 11 425 031)	86.9 (79.1 to 94.4)	30 191 062 (28 415 631 to 32 140 717)	115.1 (109.0 to 122.2)	1 174 794 (862 300 to 1 549 600)	118.2 (111.4 to 125.1)	166 430 (164 153 to 168 607)	26.6 (23.8 to 30.5)	7 880 332 (7 767 741 to 7 988 007)	8.1 (5.3 to 12.1)	9 055 126 (8 724 693 to 9 430 144)	15.6 (12.0 to 20.2)
Paraguay	312 771 (292 146 to 335 090)	107.6 (101.4 to 114.5)	722 606 (681 909 to 766 911)	114.0 (109.4 to 118.7)	28 837 (20 987 to 38 355)	100.8 (91.0 to 110.6)	3 756 (3 127 to 4 512)	113.7 (74.4 to 160.7)	182 100 (151 011 to 219 409)	83.5 (48.4 to 124.6)	210 937 (179 736 to 249 643)	85.7 (54.8 to 121.5)
**North Africa and Middle East**	**45 270 955 (42 704 903 to 47 992 132)**	**95.6 (89.9 to 103.4)**	**107 498 878 (99 870 565 to 116 912 003)**	**101.4 (98.9 to 104.0)**	**4 341 745 (3 213 242 to 5 671 131)**	**61.9 (52.8 to 72.7)**	**372 685 (354 860 to 394 403)**	**23.7 (16.6 to 38.5)**	**20 335 737 (19 459 942 to 21 417 708)**	**9.3 (1.6 to 24.4)**	**24 677 483 (23 189 581 to 26 418 484)**	**15.9 (8.3 to 30.0)**
**North Africa and Middle East**	**45 270 955 (42 704 903 to 47 992 132)**	**95.6 (89.9 to 103.4)**	**107 498 878 (99 870 565 to 116 912 003)**	**101.4 (98.9 to 104.0)**	**4 341 745 (3 213 242 to 5 671 131)**	**61.9 (52.8 to 72.7)**	**372 685 (354 860 to 394 403)**	**23.7 (16.6 to 38.5)**	**20 335 737 (19 459 942 to 21 417 708)**	**9.3 (1.6 to 24.4)**	**24 677 483 (23 189 581 to 26 418 484)**	**15.9 (8.3 to 30.0)**
Afghanistan	1 909 032 (1 793 620 to 2 034 691)	178.8 (168.1 to 188.1)	7 769 816 (6 486 456 to 9 397 136)	86.7 (78.1 to 96.2)	437 129 (312 563 to 585 613)	50.8 (35.7 to 69.3)	26 380 (21 936 to 31 285)	82.4 (34.8 to 306.7)	1 612 258 (1 348 416 to 1 904 629)	85.9 (36.1 to 350.1)	2 049 387 (1 751 207 to 2 358 802)	77.1 (38.6 to 225.6)
Algeria	2 340 201 (2 210 608 to 2 480 585)	62.5 (57.1 to 68.1)	6 398 375 (6 086 378 to 6 782 791)	109.3 (104.8 to 114.4)	246 311 (179 728 to 323 373)	67.7 (57.4 to 79.8)	14 259 (12 109 to 18 101)	-4.4 (-13.8 to 6.7)	720 652 (617 025 to 897 137)	-21.8 (-30.3 to -11.4)	966 963 (838 821 to 1 157 077)	-9.5 (-18.2 to 0.4)
Bahrain	94 039 (87 501 to 101 471)	225.0 (211.7 to 241.2)	267 818 (254 249 to 286 548)	298.3 (289.1 to 307.9)	9 269 (6 618 to 12 480)	218.7 (196.9 to 242.7)	319 (284 to 358)	69.4 (50.1 to 91.6)	15 451 (13 854 to 17 282)	46.9 (31.0 to 65.4)	24 720 (21 676 to 28 529)	84.1 (66.7 to 103.0)
Egypt	5 393 986 (5 081 312 to 5 742 651)	103.5 (98.1 to 109.4)	11 936 938 (11 414 760 to 12 651 037)	101.7 (98.1 to 105.1)	480 503 (350 037 to 640 935)	54.8 (43.9 to 67.1)	45 655 (37 988 to 52 927)	14.4 (-0.7 to 34.7)	2 384 711 (2 053 068 to 2 713 527)	-2.7 (-16.6 to 16.2)	2 865 214 (2 535 226 to 3 223 100)	3.7 (-8.9 to 21.0)
Iran	5 254 315 (4 950 320 to 5 595 243)	-9.4 (-12.7 to -6.1)	16 844 989 (15 676 252 to 18 226 291)	58.5 (50.2 to 66.5)	621 321 (460 748 to 815 677)	14.6 (1.8 to 27.8)	42 562 (41 917 to 43 689)	-53.5 (-55.5 to -51.4)	2 097 157 (2 061 146 to 2 170 883)	-63.4 (-64.7 to -61.7)	2 718 478 (2 556 405 to 2 935 525)	-56.6 (-58.8 to -54.2)
Iraq	5 921 103 (5 432 687 to 6 656 162)	269.8 (245.4 to 302.2)	11 824 042 (10 103 327 to 14 075 646)	90.0 (83.7 to 98.3)	549 136 (397 957 to 738 202)	70.2 (56.5 to 85.0)	48 501 (47 275 to 49 701)	223.4 (186.7 to 291.7)	2 951 931 (2 874 293 to 3 027 809)	226.2 (190.5 to 303.5)	3 501 067 (3 335 552 to 3 706 394)	185.2 (158.2 to 231.1)
Jordan	580 490 (544 069 to 621 115)	175.0 (164.0 to 185.2)	1 298 242 (1 237 987 to 1 378 645)	218.9 (213.6 to 224.5)	45 275 (32 400 to 60 602)	154.6 (138.5 to 172.1)	2 690 (2 435 to 2 971)	38.1 (20.0 to 63.1)	152 327 (136 541 to 169 673)	23.8 (6.0 to 47.8)	197 602 (175 258 to 221 190)	40.3 (23.4 to 63.1)
Kuwait	286 094 (266 906 to 308 020)	16.0 (2.9 to 27.1)	780 459 (739 584 to 831 786)	189.4 (183.7 to 194.9)	27 198 (19 501 to 36 640)	138.4 (124.2 to 152.4)	977 (912 to 1 049)	-52.0 (-55.1 to -48.5)	47 892 (44 816 to 51 390)	-60.7 (-63.2 to -57.9)	75 090 (67 073 to 85 509)	-43.6 (-48.6 to -37.3)
Lebanon	556 889 (525 138 to 593 319)	87.6 (77.8 to 96.7)	1 706 902 (1 534 976 to 1 922 893)	75.9 (62.3 to 90.6)	60 359 (43 832 to 80 363)	28.6 (9.0 to 48.8)	2 582 (2 274 to 2 878)	-28.4 (-38.5 to -19.3)	129 018 (113 797 to 143 400)	-38.5 (-46.8 to -30.6)	189 378 (168 281 to 213 627)	-26.2 (-34.3 to -18.8)
Libya	574 708 (538 194 to 615 741)	135.8 (124.4 to 151.6)	1 494 055 (1 333 428 to 1 701 871)	193.0 (167.1 to 225.5)	58 435 (42 781 to 77 441)	157.5 (125.2 to 212.6)	5 711 (4 765 to 6 658)	145.9 (103.8 to 192.1)	300 739 (256 547 to 344 186)	123.3 (88.2 to 163.0)	359 174 (311 802 to 406 233)	128.2 (96.3 to 161.0)
Morocco	1 911 221 (1 809 427 to 2 025 484)	50.2 (45.9 to 54.6)	5 108 820 (4 878 832 to 5 407 262)	73.3 (70.6 to 75.9)	201 244 (147 638 to 267 668)	41.2 (32.8 to 50.7)	15 792 (12 628 to 20 857)	-6.6 (-24.6 to 12.6)	771 660 (623 295 to 999 508)	-24.6 (-38.6 to -9.1)	972 904 (807 155 to 1 204 832)	-16.6 (-29.4 to -2.4)
Palestine	290 388 (271 619 to 309 667)	91.5 (80.2 to 100.8)	1 156 940 (972 104 to 1 413 050)	210.7 (193.2 to 233.1)	49 544 (35 871 to 66 724)	156.1 (140.9 to 172.3)	1 284 (1 101 to 1 413)	-3.0 (-17.1 to 11.3)	72 302 (61 638 to 80 798)	-15.4 (-28.3 to -0.2)	121 846 (105 491 to 140 815)	16.2 (2.2 to 32.3)
Oman	308 622 (286 873 to 332 818)	151.9 (140.5 to 164.4)	735 197 (699 644 to 780 939)	203.7 (198.5 to 209.0)	26 047 (18 419 to 35 248)	141.9 (125.8 to 159.0)	2 437 (1 984 to 2 942)	30.4 (3.0 to 68.0)	126 873 (104 557 to 152 154)	15.5 (-7.9 to 50.9)	152 921 (128 638 to 179 358)	26.8 (3.8 to 59.3)
Qatar	217 630 (202 847 to 234 867)	520.9 (494.2 to 549.2)	532 294 (503 266 to 569 560)	599.6 (591.0 to 609.8)	18 625 (13 220 to 25 379)	487.1 (456.6 to 519.9)	1 036 (840 to 1 258)	319.2 (228.7 to 442.8)	53 479 (43 551 to 64 706)	283.2 (202.0 to 389.8)	72 104 (59 847 to 85 022)	321.0 (250.7 to 408.7)
Saudi Arabia	2 866 925 (2 699 588 to 3 055 030)	144.9 (135.3 to 155.7)	6 046 209 (5 747 794 to 6 424 212)	168.0 (164.0 to 172.0)	216 836 (154 578 to 294 199)	113.6 (99.2 to 129.0)	22 799 (18 843 to 26 921)	73.4 (35.5 to 117.4)	1 134 274 (940 952 to 1 339 316)	50.0 (16.8 to 87.4)	1 351 110 (1 140 126 to 1 571 746)	57.5 (28.0 to 90.3)
Sudan	2 201 755 (2 068 932 to 2 336 372)	49.1 (42.2 to 55.8)	5 872 811 (5 315 332 to 6 587 349)	113.9 (107.3 to 121.2)	228 530 (171 133 to 295 549)	90.3 (83.3 to 98.8)	21 897 (17 760 to 27 413)	-23.5 (-39.8 to 5.3)	1 344 297 (1 092 483 to 1 638 749)	-30.7 (-46.6 to 0.4)	1 572 828 (1 323 868 to 1 890 207)	-23.6 (-39.8 to 7.4)
Syria	4 820 818 (4 314 697 to 5 588 353)	689.8 (602.7 to 813.6)	5 506 141 (4 548 156 to 6 820 755)	322.2 (263.1 to 397.1)	245 472 (176 307 to 338 478)	311.3 (245.9 to 420.0)	45 411 (44 766 to 46 158)	867.5 (767.7 to 1 000.2)	2 639 242 (2 610 344 to 2 673 781)	835.8 (722.1 to 987.2)	2 884 714 (2 807 085 to 2 977 936)	744.2 (657.7 to 857.6)
Tunisia	718 590 (679 790 to 763 090)	32.3 (27.3 to 37.4)	1 976 453 (1 884 312 to 2 109 319)	68.1 (65.2 to 71.3)	69 369 (49 782 to 93 442)	38.5 (29.2 to 49.1)	5 762 (4 685 to 7 023)	-0.9 (-20.6 to 22.5)	234 441 (189 609 to 283 864)	-28.2 (-42.3 to -12.4)	303 810 (253 602 to 356 877)	-19.4 (-32.4 to -5.3)
Turkey	4 984 549 (4 714 514 to 5 268 387)	36.1 (31.3 to 40.8)	14 137 597 (13 421 671 to 15 055 426)	74.3 (70.0 to 79.0)	480 145 (344 294 to 644 716)	33.1 (22.1 to 45.3)	25 861 (23 345 to 28 163)	-1.7 (-14.6 to 11.7)	1 150 319 (1 057 580 to 1 247 966)	-24.9 (-34.1 to -14.4)	1 630 464 (1 462 854 to 1 812 217)	-13.8 (-23.0 to -3.5)
United Arab Emirates	797 755 (741 099 to 858 399)	404.6 (381.0 to 431.1)	2 164 308 (2 043 496 to 2 304 739)	524.5 (511.3 to 537.1)	78 401 (56 444 to 105 784)	424.7 (391.3 to 461.6)	6 159 (4 819 to 7 649)	421.5 (286.1 to 588.9)	291 811 (228 093 to 361 387)	343.7 (231.3 to 476.3)	370 212 (300 179 to 444 164)	358.7 (264.4 to 466.1)
Yemen	3 199 569 (2 920 744 to 3 570 238)	345.5 (310.1 to 402.1)	3 840 085 (3 589 500 to 4 167 466)	149.6 (143.0 to 157.5)	188 541 (140 661 to 244 630)	125.8 (113.3 to 140.6)	34 262 (30 489 to 40 044)	130.0 (71.8 to 400.9)	2 085 913 (1 868 809 to 2 417 180)	110.1 (54.1 to 379.9)	2 274 455 (2 043 550 to 2 612 197)	111.4 (58.4 to 335.5)
**South Asia**	**104 773 040 (99 110 820 to 111 302 843)**	**86.5 (82.1 to 91.0)**	**261 230 229 (248 880 133 to 274 959 856)**	**115.6 (112.8 to 118.7)**	**10 796 160 (8 068 438 to 13 977 167)**	**96.2 (89.3 to 104.2)**	**1 167 667 (1 080 250 to 1 229 819)**	**28.0 (19.6 to 39.7)**	**51 266 858 (47 086 258 to 54 098 762)**	**-0.5 (-7.4 to 10.8)**	**62 063 019 (57 061 135 to 66 428 115)**	**8.9 (1.4 to 20.3)**
**South Asia**	**104 773 040 (99 110 820 to 111 302 843)**	**86.5 (82.1 to 91.0)**	**261 230 229 (248 880 133 to 274 959 856)**	**115.6 (112.8 to 118.7)**	**10 796 160 (8 068 438 to 13 977 167)**	**96.2 (89.3 to 104.2)**	**1 167 667 (1 080 250 to 1 229 819)**	**28.0 (19.6 to 39.7)**	**51 266 858 (47 086 258 to 54 098 762)**	**-0.5 (-7.4 to 10.8)**	**62 063 019 (57 061 135 to 66 428 115)**	**8.9 (1.4 to 20.3)**
Bangladesh	8 611 410 (8 074 502 to 9 203 668)	102.7 (96.2 to 109.6)	22 614 749 (21 425 195 to 23 973 920)	143.6 (136.6 to 151.0)	898 710 (675 301 to 1 166 836)	91.1 (77.1 to 107.5)	57 813 (51 125 to 64 878)	-36.2 (-46.7 to -18.9)	2 920 437 (2 524 456 to 3 345 659)	-54.0 (-62.3 to -40.0)	3 819 147 (3 388 116 to 4 322 549)	-43.9 (-53.0 to -28.3)
Bhutan	56 477 (53 340 to 59 946)	82.4 (75.8 to 89.0)	142 354 (135 141 to 150 089)	113.6 (108.1 to 119.2)	5 762 (4 324 to 7 476)	83.2 (73.1 to 94.9)	367 (284 to 434)	-7.0 (-30.0 to 17.1)	16 228 (11 868 to 19 289)	-29.8 (-49.4 to -9.0)	21 990 (17 421 to 25 562)	-16.3 (-36.0 to 3.8)
India	82 938 618 (78 431 974 to 87 975 214)	77.6 (73.3 to 82.2)	206 644 919 (197 155 586 to 217 281 001)	106.2 (103.6 to 109.1)	8 455 546 (6 305 699 to 10 970 453)	88.5 (81.9 to 95.9)	977 117 (893 106 to 1 029 633)	31.5 (22.6 to 42.3)	41 201 788 (37 476 195 to 43 393 174)	2.0 (-4.8 to 11.3)	49 657 335 (45 168 144 to 53 161 813)	10.7 (3.5 to 20.0)
Nepal	1 493 655 (1 407 015 to 1 589 728)	79.4 (74.2 to 84.5)	4 282 938 (4 046 131 to 4 546 052)	122.0 (115.8 to 129.1)	182 367 (138 390 to 231 736)	103.0 (93.6 to 112.9)	16 831 (13 323 to 20 579)	24.1 (-3.7 to 67.0)	715 602 (540 423 to 894 816)	-9.6 (-33.6 to 29.8)	897 969 (718 350 to 1 092 186)	1.9 (-20.9 to 40.5)
Pakistan	11 672 880 (11 002 016 to 12 424 012)	167.5 (161.0 to 173.6)	27 545 270 (26 149 495 to 29 019 414)	184.5 (178.4 to 191.6)	1 253 775 (938 240 to 1 615 812)	175.7 (165.2 to 186.3)	115 539 (90 955 to 137 658)	79.0 (46.6 to 121.5)	6 412 802 (5 095 090 to 7 629 504)	61.5 (31.5 to 105.8)	7 666 578 (6 374 623 to 8 868 483)	73.2 (45.2 to 114.0)
**Southeast Asia, East Asia, and Oceania**	**99 427 300 (93 882 709 to 105 301 532)**	**79.6 (73.8 to 85.8)**	**409 428 495 (389 282 308 to 430 617 677)**	**89.6 (83.7 to 95.7)**	**13 366 159 (9 957 437 to 17 533 460)**	**84.9 (73.8 to 96.6)**	**1 122 652 (1 065 752 to 1 159 350)**	**-14.4 (-19.4 to -10.3)**	**44 431 454 (42 652 348 to 45 735 474)**	**-40.6 (-43.6 to -37.7)**	**57 797 613 (53 826 241 to 62 476 600)**	**-29.6 (-33.8 to -25.1)**
**East Asia**	**80 525 641 (75 775 665 to 85 286 045)**	**86.9 (80.1 to 94.5)**	**346 140 027 (328 822 381 to 364 593 436)**	**88.6 (82.2 to 95.5)**	**10 637 158 (7 880 731 to 13 994 160)**	**87.1 (74.8 to 100.1)**	**775 366 (720 835 to 809 835)**	**-18.9 (-25.6 to -14.2)**	**28 720 067 (27 196 646 to 29 844 375)**	**-47.5 (-51.3 to -44.4)**	**39 357 225 (36 142 412 to 43 139 078)**	**-34.8 (-39.9 to -29.6)**
China	77 069 739 (72 520 470 to 81 649 224)	88.2 (81.2 to 95.9)	331 226 633 (314 608 899 to 348 895 773)	89.5 (83.0 to 96.6)	10 152 426 (7 520 543 to 13 366 714)	87.6 (75.1 to 100.9)	733 517 (681 254 to 767 006)	-19.7 (-26.5 to -14.8)	27 067 763 (25 600 588 to 28 162 416)	-48.4 (-52.3 to -45.2)	37 220 189 (34 160 038 to 40 804 836)	-35.6 (-40.8 to -30.5)
North Korea	1 040 575 (982 483 to 1 101 861)	88.1 (82.7 to 94.0)	4 419 634 (4 188 216 to 4 666 138)	77.3 (71.9 to 83.0)	157 185 (117 635 to 203 762)	103.9 (95.1 to 112.0)	16 542 (13 315 to 20 019)	58.3 (22.2 to 98.5)	763 802 (610 931 to 929 323)	38.7 (5.6 to 75.9)	920 987 (755 899 to 1 101 431)	46.8 (15.3 to 80.1)
Taiwan (Province of China)	1 118 104 (1 057 664 to 1 180 745)	32.0 (26.3 to 37.9)	4 917 640 (4 676 672 to 5 172 090)	52.7 (48.2 to 57.6)	156 188 (113 477 to 206 740)	52.8 (46.3 to 59.2)	12 816 (12 169 to 13 507)	-24.0 (-27.8 to -19.9)	425 839 (403 484 to 449 811)	-47.4 (-50.2 to -44.4)	582 027 (535 086 to 636 088)	-36.2 (-40.1 to -32.0)
**Oceania**	**583 144 (551 946 to 616 260)**	**146.0 (139.7 to 152.5)**	**1 544 699 (1 465 655 to 1 629 837)**	**165.2 (158.8 to 172.0)**	**65 348 (49 432 to 83 415)**	**172.5 (164.4 to 181.0)**	**12 614 (10 498 to 14 913)**	**76.8 (47.2 to 108.9)**	**706 580 (590 281 to 835 289)**	**71.3 (42.8 to 102.1)**	**771 928 (653 958 to 901 042)**	**76.9 (49.5 to 106.5)**
American Samoa	2 692 (2 547 to 2 843)	41.9 (38.0 to 45.7)	7 989 (7 585 to 8 443)	67.0 (61.6 to 73.0)	288 (216 to 374)	60.7 (53.9 to 68.5)	25 (23 to 26)	1.5 (-8.8 to 12.9)	1 185 (1 088 to 1 298)	-10.7 (-20.7 to 0.5)	1 473 (1 347 to 1 611)	-2.2 (-11.9 to 8.2)
Federated States of Micronesia	4 783 (4 507 to 5 090)	42.5 (37.7 to 47.9)	13 121 (12 482 to 13 816)	53.2 (48.9 to 57.5)	543 (403 to 700)	50.3 (43.7 to 57.1)	66 (45 to 82)	-8.7 (-37.5 to 17.4)	3 409 (2 285 to 4 310)	-16.0 (-44.0 to 8.5)	3 952 (2 837 to 4 866)	-10.6 (-36.7 to 11.8)
Fiji	39 746 (37 599 to 42 072)	64.3 (59.0 to 69.7)	120 704 (115 130 to 126 815)	75.3 (70.3 to 80.2)	4 677 (3 454 to 6 038)	75.6 (69.0 to 82.8)	409 (364 to 462)	19.3 (1.1 to 40.3)	21 255 (18 778 to 24 131)	8.9 (-7.5 to 28.8)	25 931 (23 200 to 29 096)	16.9 (1.2 to 35.3)
Guam	9 031 (8 580 to 9 505)	63.5 (58.5 to 68.9)	27 713 (26 475 to 29 041)	85.9 (80.6 to 91.2)	965 (703 to 1 281)	89.3 (81.6 to 96.3)	95 (88 to 103)	37.8 (24.6 to 52.7)	4 412 (4 057 to 4 786)	19.3 (8.0 to 32.4)	5 376 (4 931 to 5 855)	27.8 (17.1 to 39.6)
Kiribati	4 811 (4 515 to 5 139)	133.0 (125.8 to 140.5)	14 051 (13 221 to 14 910)	118.5 (111.5 to 125.4)	563 (421 to 716)	139.7 (130.2 to 149.2)	67 (55 to 78)	45.8 (19.0 to 75.8)	3 754 (3 067 to 4 441)	39.3 (13.0 to 68.5)	4 317 (3 642 to 5 003)	47.3 (22.0 to 74.3)
Marshall Islands	2 644 (2 495 to 2 804)	82.2 (75.5 to 88.9)	6 932 (6 572 to 7 315)	100.2 (94.2 to 106.2)	278 (207 to 359)	101.0 (92.5 to 109.1)	45 (38 to 54)	27.2 (7.6 to 49.9)	2 416 (2 039 to 2 864)	17.9 (-0.1 to 38.9)	2 694 (2 300 to 3 156)	23.2 (5.9 to 42.9)
Northern Mariana Islands	2 309 (2 186 to 2 430)	12.8 (8.5 to 17.2)	7 660 (7 312 to 8 022)	42.0 (36.9 to 46.8)	258 (186 to 344)	38.5 (31.7 to 45.0)	22 (20 to 25)	-7.3 (-22.3 to 11.7)	938 (824 to 1 049)	-26.7 (-39.0 to -11.3)	1 195 (1 068 to 1 339)	-18.5 (-30.0 to -4.2)
Papua New Guinea	426 135 (403 273 to 450 264)	176.1 (167.8 to 184.6)	1 110 105 (1 051 324 to 1 172 696)	201.3 (193.1 to 210.1)	47 438 (35 830 to 60 321)	208.8 (197.7 to 219.7)	10 362 (8 440 to 12 507)	87.8 (51.7 to 127.0)	586 950 (480 320 to 707 039)	82.9 (48.2 to 119.7)	634 388 (527 263 to 755 160)	88.7 (55.8 to 124.5)
Samoa	9 056 (8 559 to 9 593)	50.9 (46.3 to 55.6)	24 820 (23 582 to 26 177)	88.3 (81.4 to 96.4)	1 049 (792 to 1 341)	87.9 (80.2 to 96.5)	76 (62 to 94)	-9.4 (-27.6 to 10.6)	3 716 (2 953 to 4 628)	-21.2 (-38.4 to -2.3)	4 765 (3 876 to 5 659)	-9.7 (-26.5 to 7.7)
Solomon Islands	31 437 (29 721 to 33 292)	157.0 (149.5 to 164.3)	77 856 (73 892 to 82 080)	169.1 (162.8 to 175.2)	3 550 (2 677 to 4 534)	172.7 (165.0 to 180.2)	506 (420 to 597)	68.0 (32.6 to 108.4)	26 607 (21 980 to 31 659)	58.4 (23.6 to 95.1)	30 157 (25 278 to 35 115)	66.6 (33.5 to 100.9)
Tonga	4 557 (4 296 to 4 825)	46.0 (41.8 to 50.6)	12 441 (11 870 to 13 041)	59.2 (54.9 to 63.3)	490 (365 to 632)	50.2 (43.6 to 57.0)	44 (39 to 49)	1.7 (-13.9 to 18.8)	2 121 (1 870 to 2 409)	-8.0 (-21.9 to 7.3)	2 611 (2 315 to 2 926)	-0.8 (-13.8 to 13.3)
Vanuatu	13 809 (13 028 to 14 683)	166.8 (159.1 to 174.4)	36 186 (34 325 to 38 180)	173.4 (166.4 to 180.6)	1 649 (1 248 to 2 102)	187.1 (177.7 to 196.1)	202 (150 to 265)	92.8 (42.2 to 162.2)	10 881 (7 867 to 14 330)	86.2 (35.9 to 156.2)	12 530 (9 485 to 16 043)	95.2 (48.2 to 158.9)
**Southeast Asia**	**18 318 515 (17 267 336 to 19 402 434)**	**52.1 (48.1 to 56.7)**	**61 743 769 (58 725 880 to 65 102 415)**	**93.9 (90.0 to 97.7)**	**2 663 653 (1 993 024 to 3 428 588)**	**75.1 (67.2 to 83.7)**	**334 672 (321 302 to 348 076)**	**-3.8 (-9.5 to 2.7)**	**15 004 807 (14 339 137 to 15 621 311)**	**-23.8 (-28.6 to -18.2)**	**17 668 460 (16 679 633 to 18 667 289)**	**-16.8 (-21.8 to -10.5)**
Cambodia	520 783 (490 097 to 553 897)	57.6 (51.6 to 64.1)	1 974 812 (1 776 787 to 2 246 130)	47.9 (35.7 to 61.2)	90 742 (67 991 to 116 937)	8.6 (-9.3 to 27.8)	12 773 (10 665 to 15 094)	-5.8 (-20.4 to 12.5)	579 104 (489 915 to 681 899)	-29.4 (-41.6 to -12.5)	669 846 (580 130 to 777 663)	-25.9 (-37.2 to -9.8)
Indonesia	5 505 409 (5 077 919 to 5 948 462)	34.3 (28.6 to 40.2)	20 555 326 (19 417 859 to 21 681 873)	63.3 (59.3 to 67.3)	933 592 (706 206 to 1 188 082)	58.4 (53.1 to 64.5)	86 670 (81 900 to 91 497)	-25.0 (-31.1 to -17.6)	3 903 039 (3 686 064 to 4 131 948)	-42.5 (-47.3 to -35.2)	4 836 631 (4 487 234 to 5 177 499)	-34.4 (-39.8 to -26.8)
Laos	183 355 (172 078 to 195 542)	46.6 (40.0 to 54.4)	535 658 (510 319 to 565 105)	106.0 (102.4 to 109.8)	25 648 (19 157 to 33 277)	85.5 (76.6 to 94.4)	4 195 (3 183 to 4 900)	-18.4 (-39.5 to 2.7)	229 543 (173 494 to 269 083)	-29.0 (-47.5 to -8.8)	255 191 (198 245 to 295 436)	-24.3 (-41.8 to -3.6)
Malaysia	1 018 972 (957 601 to 1 084 245)	138.7 (128.4 to 149.4)	2 882 770 (2 754 011 to 3 021 916)	155.2 (149.5 to 160.7)	118 765 (85 728 to 157 710)	120.9 (107.1 to 135.7)	14 718 (13 416 to 16 239)	57.6 (37.5 to 76.3)	640 672 (586 142 to 704 941)	37.0 (22.1 to 52.7)	759 437 (692 480 to 834 600)	45.6 (30.7 to 59.8)
Maldives	13 068 (12 130 to 14 162)	176.5 (161.7 to 193.8)	39 460 (37 426 to 41 725)	244.0 (232.1 to 256.8)	1 462 (1 073 to 1 918)	166.4 (145.6 to 190.1)	99 (89 to 128)	-24.8 (-35.1 to -2.4)	4 330 (3 852 to 5 591)	-46.5 (-55.0 to -27.1)	5 792 (5 119 to 7 174)	-33.0 (-43.2 to -12.3)
Mauritius	36 082 (34 009 to 38 253)	69.4 (62.2 to 76.3)	126 157 (120 557 to 132 530)	78.2 (72.6 to 84.0)	4 785 (3 479 to 6 344)	74.5 (65.6 to 83.0)	546 (505 to 584)	7.2 (-1.0 to 15.4)	23 099 (21 361 to 24 706)	-10.1 (-17.0 to -3.1)	27 883 (25 829 to 30 078)	-2.0 (-8.5 to 4.9)
Myanmar	2 203 912 (2 056 295 to 2 389 135)	147.8 (133.3 to 166.5)	5 563 706 (5 168 529 to 6 055 505)	113.5 (101.4 to 128.5)	278 946 (211 327 to 356 696)	93.4 (74.8 to 113.0)	37 701 (33 389 to 42 462)	-6.8 (-22.1 to 10.8)	1 791 639 (1 577 879 to 2 042 852)	-24.3 (-37.7 to -7.6)	2 070 584 (1 839 661 to 2 321 085)	-17.5 (-30.9 to -1.7)
Philippines	2 813 995 (2 652 648 to 2 982 498)	63.1 (56.6 to 70.3)	8 304 443 (7 898 549 to 8 765 890)	122.3 (116.5 to 128.1)	338 352 (253 754 to 435 562)	116.3 (107.2 to 125.7)	51 491 (45 355 to 58 796)	38.6 (20.4 to 59.1)	2 640 335 (2 332 629 to 3 017 042)	21.3 (5.6 to 39.4)	2 978 686 (2 648 673 to 3 354 802)	27.6 (13.0 to 44.9)
Sri Lanka	625 133 (593 450 to 659 381)	-49.3 (-53.2 to -45.3)	3 315 438 (2 926 738 to 3 824 621)	131.3 (115.5 to 147.8)	128 610 (96 279 to 167 380)	84.4 (67.5 to 104.9)	13 688 (11 449 to 16 185)	-33.9 (-44.3 to -22.1)	513 566 (421 327 to 611 890)	-51.7 (-60.4 to -42.6)	642 177 (546 852 to 750 689)	-43.3 (-51.3 to -34.3)
Seychelles	3 132 (2 971 to 3 308)	98.0 (90.7 to 105.2)	10 384 (9 936 to 10 877)	113.0 (107.4 to 118.5)	404 (294 to 537)	95.8 (85.1 to 107.1)	59 (56 to 63)	33.0 (23.0 to 43.4)	2 479 (2 318 to 2 637)	24.3 (14.1 to 33.7)	2 884 (2 689 to 3 083)	31.0 (21.7 to 40.3)
Thailand	2 400 774 (2 278 498 to 2 530 892)	46.1 (39.9 to 52.5)	8 870 309 (8 472 469 to 9 306 466)	89.3 (84.8 to 93.8)	350 969 (254 317 to 465 331)	63.7 (53.6 to 75.0)	48 268 (43 321 to 53 332)	-9.9 (-22.1 to 2.3)	2 131 507 (1 907 823 to 2 355 565)	-27.3 (-36.9 to -17.4)	2 482 476 (2 223 231 to 2 738 530)	-21.1 (-30.6 to -11.4)
Timor-Leste	30 349 (28 481 to 32 390)	-28.9 (-34.8 to -22.7)	179 232 (147 325 to 221 142)	63.4 (58.5 to 69.2)	8 910 (6 405 to 12 059)	24.5 (13.6 to 37.4)	466 (285 to 568)	-35.9 (-59.9 to -20.7)	24 766 (13 353 to 30 125)	-48.4 (-71.0 to -34.4)	33 676 (22 745 to 40 222)	-38.9 (-58.8 to -25.8)
Vietnam	2 939 448 (2 785 414 to 3 116 625)	98.5 (90.9 to 106.9)	9 304 834 (8 886 250 to 9 753 241)	142.6 (137.4 to 147.9)	378 964 (278 170 to 499 514)	107.2 (94.1 to 120.1)	63 559 (55 503 to 70 312)	25.8 (6.8 to 46.0)	2 500 985 (2 135 104 to 2 764 958)	-6.0 (-22.1 to 11.1)	2 879 949 (2 500 758 to 3 165 653)	1.3 (-14.6 to 17.5)
**Sub-Saharan Africa**	**54 685 108 (51 560 138 to 58 171 113)**	**67.1 (62.5 to 71.9)**	**126 208 599 (119 365 773 to 133 579 547)**	**107.7 (105.5 to 109.7)**	**5 653 767 (4 241 831 to 7 208 324)**	**85.7 (81.1 to 91.2)**	**537 054 (502 524 to 574 563)**	**18.4 (8.9 to 35.1)**	**29 571 963 (27 350 560 to 31 823 478)**	**7.4 (-3.3 to 27.1)**	**35 225 730 (32 519 746 to 37 887 674)**	**15.2 (4.5 to 33.8)**
**Central sub-Saharan Africa**	**6 364 408 (5 982 506 to 6 762 573)**	**118.8 (114.0 to 123.3)**	**16 929 157 (15 788 193 to 18 364 363)**	**150.2 (142.9 to 158.4)**	**733 037 (552 505 to 929 340)**	**124.5 (117.8 to 132.7)**	**79 034 (69 971 to 89 183)**	**40.1 (19.2 to 84.2)**	**4 770 805 (4 129 973 to 5 484 570)**	**26.6 (5.7 to 70.3)**	**5 503 842 (4 798 674 to 6 285 116)**	**34.4 (12.7 to 76.7)**
Angola	1 368 099 (1 287 717 to 1 460 224)	64.9 (53.2 to 74.7)	4 088 867 (3 723 639 to 4 569 657)	126.4 (117.8 to 135.3)	180 035 (135 125 to 229 757)	73.1 (58.1 to 87.3)	15 619 (13 370 to 18 396)	-6.5 (-26.6 to 40.9)	944 564 (797 458 to 1 126 529)	-17.5 (-36.5 to 29.1)	1 124 598 (971 329 to 1 312 669)	-10.0 (-28.9 to 34.9)
Central African Republic	411 526 (377 622 to 456 655)	232.7 (209.0 to 270.4)	632 697 (583 710 to 695 969)	135.7 (119.4 to 156.8)	34 620 (25 325 to 44 652)	161.9 (137.6 to 196.9)	8 055 (6 305 to 9 523)	117.7 (75.9 to 253.0)	485 151 (375 227 to 584 500)	108.3 (64.7 to 253.7)	519 771 (412 159 to 619 822)	111.2 (68.4 to 247.5)
Congo (Brazzaville)	249 297 (234 989 to 264 201)	99.9 (94.3 to 105.3)	773 670 (716 497 to 851 432)	169.0 (151.4 to 192.9)	34 882 (26 063 to 44 764)	157.3 (137.2 to 186.1)	2 944 (2 390 to 3 568)	19.9 (-4.8 to 55.8)	157 592 (125 494 to 192 734)	5.0 (-17.8 to 41.6)	192 474 (158 236 to 230 345)	17.6 (-4.8 to 50.8)
DR Congo	4 174 719 (3 915 051 to 4 438 594)	138.4 (131.9 to 144.2)	11 024 111 (10 349 028 to 11 825 188)	161.9 (148.7 to 178.0)	467 309 (354 362 to 590 271)	150.4 (136.6 to 170.3)	50 895 (42 445 to 59 758)	58.4 (29.0 to 115.9)	3 103 838 (2 536 614 to 3 715 686)	43.9 (15.2 to 100.9)	3 571 147 (2 968 101 to 4 240 306)	52.4 (24.0 to 107.7)
Equatorial Guinea	66 711 (62 453 to 71 309)	219.8 (209.2 to 230.9)	162 119 (152 020 to 172 457)	242.2 (234.2 to 251.1)	5 687 (4 288 to 7 333)	144.9 (129.1 to 163.0)	516 (359 to 715)	-10.0 (-38.0 to 31.2)	29 640 (20 428 to 41 187)	-20.3 (-45.7 to 20.4)	35 327 (25 952 to 47 383)	-10.6 (-35.7 to 30.3)
Gabon	94 057 (88 699 to 99 569)	59.3 (55.4 to 63.4)	247 692 (236 410 to 259 728)	68.1 (65.3 to 70.9)	10 505 (7 876 to 13 570)	55.4 (50.8 to 60.2)	1 005 (823 to 1 189)	19.4 (-2.0 to 45.4)	50 021 (39 801 to 60 127)	6.6 (-15.0 to 33.6)	60 525 (50 328 to 71 073)	12.7 (-6.8 to 36.5)
**Eastern sub-Saharan Africa**	**23 568 449 (22 144 855 to 25 182 073)**	**35.3 (29.6 to 41.3)**	**56 578 007 (52 982 108 to 60 543 637)**	**103.1 (100.4 to 105.7)**	**2 596 485 (1 949 403 to 3 300 809)**	**77.9 (70.9 to 84.8)**	**188 178 (175 587 to 203 937)**	**-5.7 (-16.0 to 11.5)**	**9 893 685 (9 195 459 to 10 815 562)**	**-17.4 (-27.8 to 2.3)**	**12 490 170 (11 474 442 to 13 710 824)**	**-7.0 (-18.1 to 11.2)**
Burundi	616 451 (576 540 to 659 192)	84.8 (79.1 to 90.9)	2 858 790 (2 407 074 to 3 403 086)	263.2 (205.0 to 333.2)	144 720 (104 563 to 192 719)	297.6 (223.1 to 414.8)	5 604 (4 733 to 6 762)	17.2 (-3.8 to 50.4)	298 643 (250 811 to 358 183)	11.6 (-10.1 to 48.1)	443 364 (377 618 to 520 508)	45.8 (20.2 to 84.4)
Comoros	42 938 (40 405 to 45 672)	43.8 (39.6 to 48.2)	93 675 (89 247 to 98 838)	63.6 (61.3 to 66.1)	4 757 (3 506 to 6 132)	46.3 (41.1 to 51.8)	352 (291 to 440)	6.3 (-12.6 to 31.0)	15 401 (12 553 to 19 560)	-16.4 (-32.2 to 4.9)	20 158 (16 935 to 24 770)	-7.0 (-21.6 to 11.3)
Djibouti	67 408 (63 423 to 71 761)	82.8 (74.8 to 90.4)	168 104 (159 263 to 177 652)	169.5 (163.5 to 176.0)	7 900 (5 896 to 10 191)	133.6 (124.2 to 144.8)	511 (371 to 728)	48.1 (6.2 to 118.9)	24 442 (17 524 to 35 695)	15.2 (-18.5 to 75.3)	32 343 (25 320 to 43 113)	31.5 (1.2 to 84.6)
Eritrea	339 504 (319 017 to 361 968)	-82.7 (-84.0 to -81.3)	1 482 763 (1 274 055 to 1 760 420)	14.5 (10.8 to 18.9)	78 066 (56 038 to 103 152)	-23.5 (-31.6 to -12.7)	3 956 (3 176 to 4 878)	-88.2 (-90.4 to -85.3)	210 001 (168 276 to 259 378)	-90.4 (-92.2 to -88.1)	288 067 (240 946 to 341 858)	-87.4 (-89.3 to -85.0)
Ethiopia	5 861 501 (5 483 184 to 6 286 467)	-14.5 (-20.8 to -7.7)	13 886 462 (12 933 583 to 14 874 465)	88.3 (84.5 to 92.2)	687 438 (510 140 to 877 383)	61.1 (54.4 to 68.3)	41 834 (37 703 to 46 584)	-36.7 (-47.4 to -19.7)	2 158 104 (1 932 626 to 2 428 993)	-45.7 (-55.3 to -29.2)	2 845 542 (2 551 985 to 3 181 089)	-35.3 (-46.2 to -18.4)
Kenya	3 240 071 (3 034 076 to 3 472 821)	145.6 (141.5 to 150.0)	7 641 309 (7 224 833 to 8 093 734)	166.4 (162.1 to 170.8)	277 273 (208 915 to 361 222)	164.3 (159.6 to 169.7)	22 053 (20 073 to 25 209)	97.3 (75.9 to 118.2)	1 048 571 (951 296 to 1 206 589)	78.9 (62.7 to 100.2)	1 325 844 (1 203 614 to 1 505 266)	91.9 (76.2 to 111.0)
Madagascar	1 520 064 (1 423 727 to 1 622 813)	99.3 (93.3 to 105.5)	3 113 546 (2 957 631 to 3 289 666)	98.0 (95.1 to 100.8)	148 049 (109 874 to 191 476)	85.7 (80.1 to 90.8)	10 445 (8 652 to 12 525)	24.6 (3.0 to 53.9)	553 306 (459 843 to 669 572)	12.5 (-8.5 to 47.8)	701 355 (596 576 to 823 031)	22.7 (3.9 to 53.7)
Malawi	899 045 (841 188 to 965 255)	78.7 (74.1 to 83.7)	2 148 358 (2 034 028 to 2 273 945)	90.1 (84.2 to 96.1)	85 438 (63 948 to 109 159)	69.4 (63.6 to 75.2)	7 202 (6 230 to 8 335)	4.5 (-23.9 to 135.6)	354 775 (300 939 to 423 020)	-13.0 (-40.0 to 130.9)	440 213 (378 678 to 510 303)	-3.9 (-31.0 to 114.7)
Mozambique	1 672 096 (1 570 181 to 1 791 310)	70.5 (62.2 to 78.2)	4 046 535 (3 735 685 to 4 440 438)	41.8 (29.2 to 55.0)	174 038 (130 847 to 220 562)	-0.3 (-19.0 to 17.5)	16 786 (14 574 to 19 342)	21.1 (2.1 to 43.6)	883 076 (760 392 to 1 017 757)	5.9 (-13.0 to 29.1)	1 057 114 (927 804 to 1 201 266)	4.8 (-11.1 to 24.4)
Rwanda	698 450 (654 282 to 749 299)	-2.6 (-10.5 to 4.1)	2 776 569 (2 462 377 to 3 152 783)	173.0 (141.6 to 207.4)	158 531 (113 020 to 217 063)	200.7 (147.4 to 283.4)	6 129 (5 082 to 7 625)	-37.3 (-48.1 to -19.6)	299 605 (246 114 to 380 534)	-47.3 (-57.1 to -28.5)	458 136 (382 620 to 554 406)	-26.2 (-38.8 to -5.7)
Somalia	1 533 270 (1 418 928 to 1 677 644)	129.3 (125.5 to 133.5)	2 482 721 (2 279 195 to 2 757 010)	152.4 (146.2 to 159.2)	139 164 (102 160 to 178 456)	140.8 (133.6 to 147.7)	17 315 (14 083 to 21 581)	104.8 (49.1 to 239.5)	994 080 (813 716 to 1 229 486)	90.2 (36.5 to 230.0)	1 133 244 (955 232 to 1 369 530)	95.2 (45.0 to 215.0)
South Sudan	1 064 052 (981 175 to 1 167 827)	126.4 (119.3 to 135.6)	1 603 819 (1 469 974 to 1 782 864)	115.1 (101.4 to 135.0)	84 035 (61 860 to 107 834)	122.6 (106.7 to 146.5)	10 341 (9 042 to 12 060)	101.0 (51.5 to 208.7)	618 994 (544 017 to 715 077)	107.6 (55.1 to 233.9)	703 029 (627 606 to 797 526)	109.2 (60.9 to 214.8)
Tanzania	2 983 485 (2 797 121 to 3 200 066)	116.7 (111.3 to 121.9)	6 641 448 (6 336 448 to 6 981 199)	115.7 (109.5 to 121.8)	286 683 (215 100 to 369 307)	106.8 (100.4 to 113.3)	22 209 (19 342 to 25 570)	55.4 (24.1 to 163.0)	1 187 486 (1 011 487 to 1 413 587)	45.7 (10.4 to 180.2)	1 474 169 (1 277 583 to 1 719 454)	54.6 (21.6 to 160.9)
Uganda	2 083 428 (1 940 255 to 2 244 070)	120.9 (113.4 to 128.0)	5 475 395 (5 088 583 to 5 939 961)	80.2 (66.5 to 93.8)	226 599 (171 532 to 286 855)	54.6 (31.1 to 76.2)	15 684 (13 369 to 18 915)	52.6 (25.8 to 88.7)	837 508 (698 408 to 1 012 057)	37.3 (10.3 to 77.5)	1 064 107 (912 336 to 1 256 851)	40.6 (17.7 to 73.9)
Zambia	931 897 (871 431 to 1 002 138)	105.6 (99.4 to 111.5)	2 123 011 (2 018 419 to 2 240 468)	117.7 (111.9 to 123.6)	92 164 (69 069 to 118 569)	108.2 (102.2 to 114.0)	7 640 (6 637 to 8 761)	23.3 (-3.2 to 91.8)	403 484 (343 541 to 474 359)	9.4 (-16.9 to 90.5)	495 648 (431 671 to 572 185)	20.0 (-5.6 to 93.8)
**Southern sub-Saharan Africa**	**4 334 587 (4 092 781 to 4 594 135)**	**39.6 (36.9 to 42.6)**	**10 854 500 (10 416 568 to 11 398 255)**	**50.8 (48.3 to 52.7)**	**442 873 (327 865 to 576 735)**	**34.7 (30.7 to 39.1)**	**63 603 (59 356 to 67 546)**	**0.6 (-8.1 to 8.2)**	**3 196 750 (2 964 743 to 3 422 516)**	**-10.3 (-17.1 to -3.4)**	**3 639 623 (3 366 744 to 3 909 570)**	**-6.5 (-13.1 to -0.1)**

Location	Incidence (95% UI)		Prevalence (95% UI)		Years lived with disability (95% UI)		Deaths (95% UI)		Years of life lost (95% UI)		Disability adjusted life years (95% UI)	
	2017 all-age counts	Percentage change in age-standardised rates between 1990 and 2017	2017 all-age counts	Percentage change in age-standardised rates between 1990 and 2017	2017 all-age counts	Percentage change in age-standardised rates between 1990 and 2017	2017 all-age counts	Percentage change in age-standardised rates between 1990 and 2017	2017 all-age counts	Percentage change in age-standardised rates between 1990 and 2017	2017 all-age counts	Percentage change in age-standardised rates between 1990 and 2017
Botswana	119 141 (112 509 to 126 613)	76.8 (71.7 to 82.5)	289 474 (276 883 to 304 230)	114.8 (111.1 to 118.3)	11 474 (8 495 to 15 012)	88.0 (79.0 to 97.3)	1 000 (885 to 1 141)	42.9 (14.1 to 78.7)	50 058 (43 923 to 57 253)	30.9 (3.3 to 65.7)	61 532 (54 422 to 69 696)	38.7 (12.2 to 69.4)
Lesotho	111 490 (105 647 to 117 744)	28.6 (25.2 to 31.8)	257 621 (246 636 to 270 344)	38.6 (36.7 to 40.5)	11 371 (8 458 to 14 670)	29.3 (25.3 to 33.5)	2 725 (2 170 to 3 260)	54.9 (20.0 to 93.7)	142 647 (112 973 to 170 717)	53.9 (19.1 to 93.0)	154 018 (123 512 to 183 029)	51.7 (19.9 to 86.9)
Namibia	128 097 (121 160 to 135 509)	65.5 (61.3 to 69.3)	352 779 (328 790 to 384 566)	40.2 (29.3 to 51.1)	14 639 (10 976 to 18 719)	2.9 (-13.7 to 17.9)	1 450 (1 165 to 1 788)	15.4 (-7.5 to 42.9)	74 288 (59 033 to 93 026)	9.7 (-13.1 to 36.9)	88 927 (72 839 to 108 011)	8.5 (-10.2 to 31.2)
South Africa	3 179 786 (2 993 283 to 3 378 081)	33.5 (30.3 to 36.8)	7 983 958 (7 653 886 to 8 394 911)	46.7 (44.0 to 48.9)	323 361 (238 876 to 426 215)	26.7 (22.0 to 32.2)	46 539 (43 015 to 50 099)	-12.3 (-19.6 to -4.8)	2 310 594 (2 117 443 to 2 512 623)	-23.5 (-29.7 to -16.8)	2 633 955 (2 416 478 to 2 871 873)	-19.6 (-25.7 to -13.2)
Swaziland	62 904 (59 444 to 66 525)	52.5 (48.4 to 56.4)	136 733 (130 720 to 143 611)	63.2 (60.8 to 65.6)	5 842 (4 318 to 7 540)	47.4 (41.6 to 53.6)	1 130 (906 to 1 377)	52.8 (16.8 to 93.1)	61 487 (49 132 to 74 975)	44.7 (11.9 to 82.7)	67 329 (54 755 to 80 593)	44.9 (14.9 to 79.4)
Zimbabwe	733 167 (690 122 to 778 652)	62.5 (59.1 to 66.2)	1 833 935 (1 751 947 to 1 929 801)	66.7 (61.5 to 71.6)	76 186 (57 394 to 96 836)	87.5 (81.9 to 93.3)	10 759 (9 223 to 12 268)	88.9 (51.7 to 127.0)	557 677 (478 826 to 637 041)	84.4 (50.7 to 121.9)	633 863 (550 137 to 712 991)	84.8 (54.4 to 117.7)
**Western sub-Saharan Africa**	**20 417 663 (19 267 327 to 21 714 732)**	**119.7 (114.9 to 124.1)**	**41 846 936 (39 932 900 to 43 978 994)**	**120.8 (117.5 to 124.2)**	**1 881 372 (1 403 207 to 2 421 809)**	**102.5 (97.3 to 108.5)**	**206 238 (185 481 to 229 789)**	**53.2 (37.4 to 77.7)**	**11 710 722 (10 340 764 to 13 150 150)**	**42.4 (25.3 to 71.5)**	**13 592 094 (12 170 189 to 15 084 676)**	**48.6 (32.1 to 75.7)**
Benin	547 663 (515 112 to 584 292)	133.5 (127.2 to 139.5)	1 069 847 (1 021 467 to 1 122 598)	138.7 (135.8 to 141.4)	53 455 (39 318 to 69 415)	118.9 (111.8 to 125.5)	7 139 (5 658 to 8 916)	47.7 (20.7 to 79.7)	382 798 (302 870 to 484 642)	29.9 (3.6 to 66.1)	436 253 (354 871 to 537 992)	36.7 (11.0 to 70.6)
Burkina Faso	970 339 (914 706 to 1 031 359)	121.3 (115.5 to 126.9)	1 739 851 (1 662 074 to 1 826 122)	123.2 (120.5 to 125.7)	87 663 (64 669 to 113 497)	109.6 (101.8 to 116.6)	13 376 (11 368 to 15 887)	70.9 (44.7 to 104.4)	747 841 (608 549 to 924 701)	63.7 (33.9 to 103.8)	835 504 (690 942 to 1 013 777)	67.5 (40.2 to 104.5)
Cameroon	1 314 870 (1 238 718 to 1 398 161)	165.7 (158.5 to 172.5)	2 781 530 (2 639 705 to 2 933 552)	161.1 (157.2 to 165.5)	124 468 (92 109 to 159 886)	136.1 (128.3 to 143.6)	13 617 (11 133 to 16 208)	91.0 (55.0 to 131.8)	708 198 (573 709 to 844 222)	75.6 (38.9 to 120.9)	832 665 (689 027 to 976 616)	82.6 (51.0 to 122.4)
Cape Verde	27 482 (26 037 to 29 156)	72.3 (67.2 to 77.6)	70 221 (67 077 to 73 758)	101.1 (97.9 to 104.3)	2 678 (1 983 to 3 519)	74.6 (66.4 to 83.3)	302 (274 to 331)	73.3 (51.4 to 95.7)	13 980 (12 589 to 15 443)	45.4 (24.9 to 67.3)	16 658 (14 942 to 18 392)	49.4 (31.1 to 69.0)
Chad	717 814 (675 390 to 765 882)	121.3 (111.9 to 129.2)	1 572 148 (1 475 032 to 1 692 396)	122.7 (114.2 to 131.2)	75 266 (55 964 to 95 876)	96.9 (79.7 to 111.0)	9 447 (8 026 to 11 090)	98.4 (66.4 to 137.0)	573 102 (478 332 to 683 243)	96.6 (60.5 to 145.0)	648 369 (549 234 to 758 565)	96.6 (64.3 to 138.7)
Cote d'Ivoire	1 156 431 (1 091 676 to 1 229 321)	103.6 (98.6 to 108.6)	2 412 925 (2 307 124 to 2 528 495)	121.4 (118.0 to 124.7)	120 702 (88 971 to 155 492)	113.5 (107.5 to 119.7)	14 428 (12 378 to 16 754)	73.7 (45.9 to 108.2)	776 077 (656 870 to 904 853)	56.4 (29.6 to 89.6)	896 778 (774 854 to 1 039 720)	62.3 (37.7 to 92.6)
The Gambia	98 599 (92 614 to 105 278)	126.9 (121.7 to 131.7)	191 192 (182 584 to 200 706)	122.5 (116.6 to 127.4)	9 160 (6 801 to 11 855)	102.7 (92.2 to 111.7)	934 (740 to 1 165)	83.8 (50.7 to 126.5)	44 734 (33 548 to 58 382)	48.7 (19.7 to 93.1)	53 894 (42 580 to 68 154)	55.8 (29.4 to 94.4)
Ghana	1 497 224 (1 415 729 to 1 584 086)	122.5 (117.4 to 127.7)	3 320 512 (3 170 538 to 3 493 267)	138.0 (135.0 to 140.9)	144 361 (107 924 to 186 699)	113.7 (105.3 to 122.2)	15 255 (13 097 to 17 537)	93.3 (60.9 to 131.7)	724 589 (612 533 to 852 671)	66.3 (37.9 to 103.1)	868 950 (749 523 to 1 008 428)	72.6 (46.0 to 104.2)
Guinea	550 185 (519 518 to 584 914)	82.3 (77.8 to 87.5)	1 093 908 (1 044 800 to 1 147 257)	78.4 (75.9 to 81.1)	54 959 (40 747 to 70 850)	64.7 (59.9 to 69.5)	6 957 (5 883 to 8 064)	21.6 (-0.3 to 50.8)	369 627 (306 527 to 435 799)	2.2 (-19.3 to 36.5)	424 586 (360 732 to 495 241)	7.5 (-12.5 to 39.3)
Guinea-Bissau	81 457 (76 638 to 86 778)	64.2 (59.8 to 68.7)	170 594 (162 083 to 179 459)	77.4 (73.8 to 81.5)	8 521 (6 337 to 10 910)	65.3 (59.5 to 71.6)	1 181 (969 to 1 438)	10.5 (-12.1 to 42.5)	64 406 (51 193 to 79 967)	0.9 (-21.4 to 36.3)	72 927 (59 378 to 88 246)	5.7 (-15.6 to 38.6)
Liberia	201 301 (188 661 to 215 676)	-53.5 (-60.3 to -47.4)	637 323 (566 066 to 730 740)	152.3 (130.5 to 178.5)	29 505 (21 941 to 37 892)	107.2 (84.6 to 133.5)	1 793 (1 485 to 2 243)	-66.8 (-72.6 to -58.0)	92 304 (73 854 to 121 618)	-72.7 (-78.3 to -62.9)	121 809 (101 394 to 151 930)	-65.5 (-71.2 to -55.7)
Mali	1 005 539 (944 251 to 1 073 030)	122.6 (117.1 to 127.8)	2 239 927 (2 103 924 to 2 406 164)	144.5 (132.9 to 159.4)	98 138 (73 676 to 125 211)	116.1 (103.1 to 133.0)	12 555 (10 492 to 15 127)	42.6 (17.0 to 80.3)	807 417 (655 802 to 980 644)	38.3 (10.5 to 79.5)	905 555 (752 210 to 1 083 319)	44.0 (17.4 to 83.5)
Mauritania	178 446 (167 991 to 190 444)	75.7 (71.0 to 80.5)	357 982 (341 386 to 375 894)	81.3 (79.3 to 83.5)	16 444 (12 188 to 21 308)	59.7 (53.8 to 66.1)	1 690 (1 457 to 1 972)	14.7 (-3.2 to 43.4)	82 840 (70 840 to 99 030)	6.7 (-12.4 to 38.6)	99 284 (85 970 to 116 647)	12.9 (-4.3 to 42.0)
Niger	973 724 (911 465 to 1 043 408)	142.7 (135.9 to 150.0)	1 664 024 (1 586 743 to 1 748 206)	137.2 (134.1 to 140.4)	84 665 (62 638 to 109 348)	116.6 (109.5 to 124.5)	10 464 (8 422 to 12 930)	29.6 (-0.6 to 80.2)	649 901 (509 596 to 812 210)	13.0 (-15.5 to 64.8)	734 566 (593 816 to 901 229)	19.6 (-8.9 to 69.3)
Nigeria	9 704 377 (9 133 853 to 10 335 335)	140.0 (133.9 to 146.1)	19 438 813 (18 510 887 to 20 461 643)	112.1 (106.9 to 116.6)	822 871 (614 871 to 1 065 791)	95.3 (88.3 to 102.9)	82 759 (66 888 to 102 684)	58.0 (31.6 to 99.1)	4 943 594 (3 958 862 to 6 102 794)	56.0 (28.1 to 96.9)	5 766 465 (4 735 767 to 6 954 705)	60.6 (35.3 to 97.3)
Sao Tome and Principe	10 107 (9 517 to 10 793)	69.4 (63.6 to 75.0)	22 294 (21 176 to 23 580)	87.6 (84.6 to 90.4)	985 (734 to 1 276)	64.1 (58.1 to 69.6)	76 (65 to 88)	26.6 (4.2 to 53.5)	3 822 (3 312 to 4 446)	3.4 (-15.2 to 28.9)	4 807 (4 185 to 5 524)	11.9 (-5.2 to 34.5)
Senegal	669 122 (630 087 to 712 079)	80.6 (75.9 to 85.3)	1 366 572 (1 307 786 to 1 435 402)	98.5 (96.0 to 101.1)	67 842 (50 299 to 87 761)	86.1 (81.0 to 91.3)	6 517 (5 588 to 7 974)	35.9 (14.1 to 69.3)	314 367 (265 101 to 406 031)	9.1 (-11.9 to 47.0)	382 209 (328 275 to 478 242)	17.8 (-2.0 to 51.7)
Sierra Leone	370 694 (348 846 to 394 304)	106.8 (100.5 to 112.5)	972 638 (890 813 to 1 079 821)	150.4 (131.1 to 176.3)	45 579 (33 883 to 58 390)	137.3 (117.3 to 167.1)	4 167 (3 515 to 4 883)	29.1 (4.3 to 68.7)	232 685 (193 436 to 276 757)	12.0 (-12.0 to 53.9)	278 264 (236 960 to 324 388)	22.6 (-1.7 to 63.5)
Togo	342 088 (321 977 to 364 788)	108.5 (102.9 to 114.1)	724 222 (692 805 to 759 138)	127.5 (123.1 to 131.6)	34 091 (25 266 to 44 111)	109.0 (101.4 to 117.3)	3 580 (2 969 to 4 295)	63.6 (33.2 to 104.2)	178 325 (146 766 to 215 876)	32.9 (6.5 to 71.5)	212 416 (178 883 to 251 240)	41.1 (16.4 to 75.8)
