# Supplementary figures and images for: Global injury morbidity and mortality from 1990 to 2017: results from the Global Burden of Disease Study 2017
Source: Inj Prev. 2020 Apr 24;26(Suppl 1):i96–i114. doi: 10.1136/injuryprev-2019-043494 (PMC7571366; doi:10.1136/injuryprev-2019-043494)

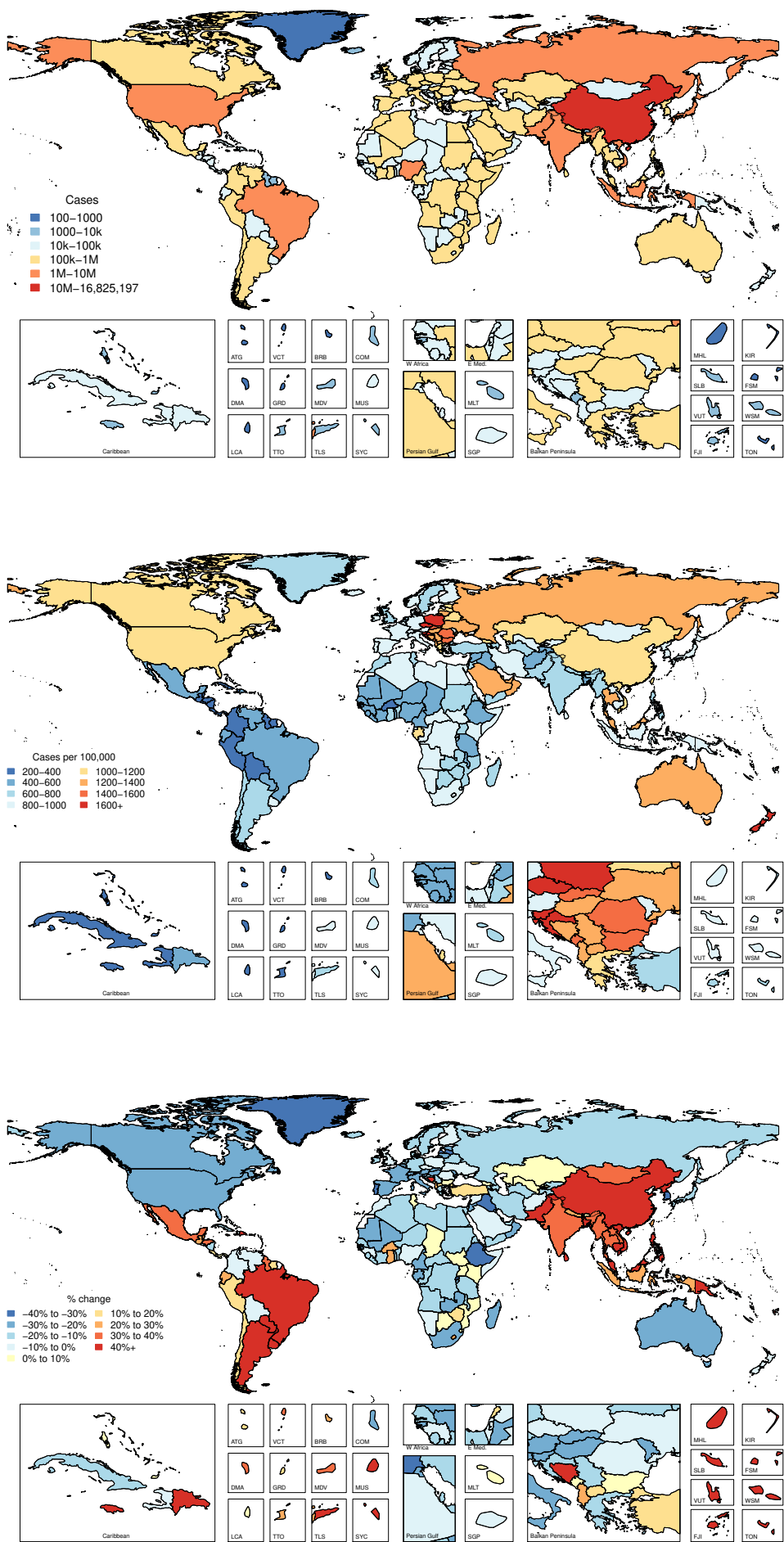

Supplement: Supplementary data [file injuryprev-2019-043494supp006.pdf]

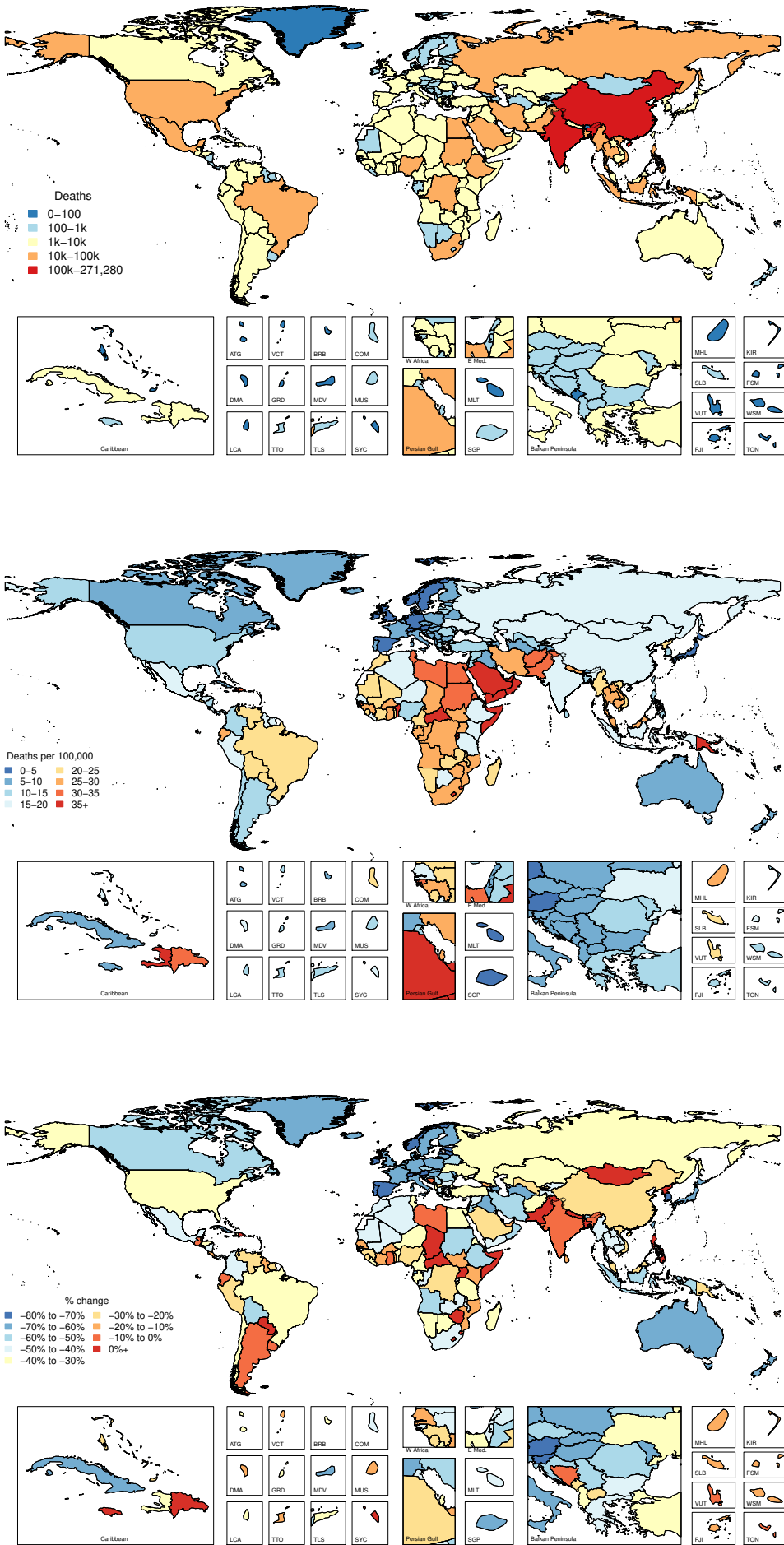

Supplement: Supplementary data [file injuryprev-2019-043494supp007.pdf]

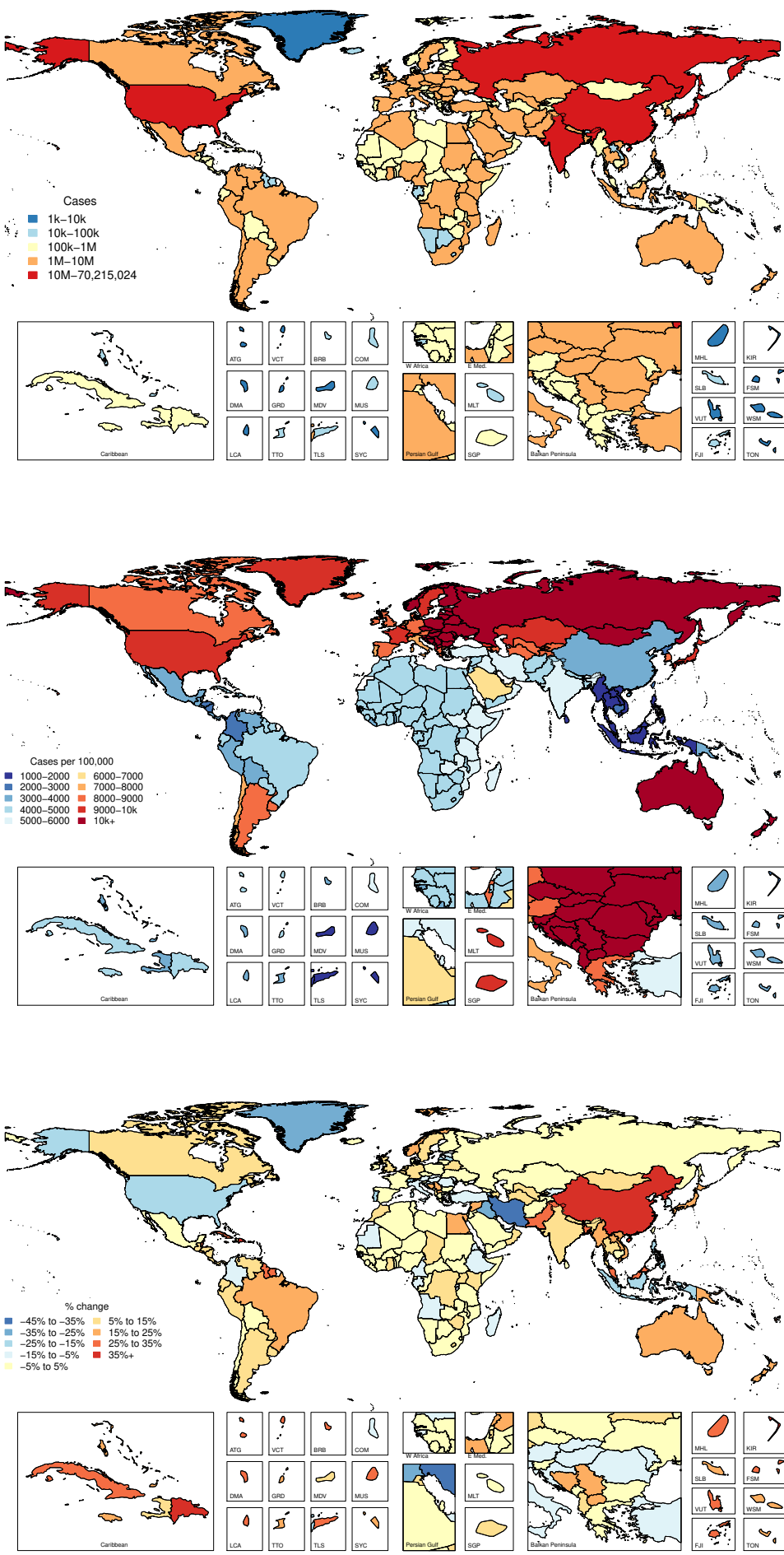

Supplement: Supplementary data [file injuryprev-2019-043494supp008.pdf]

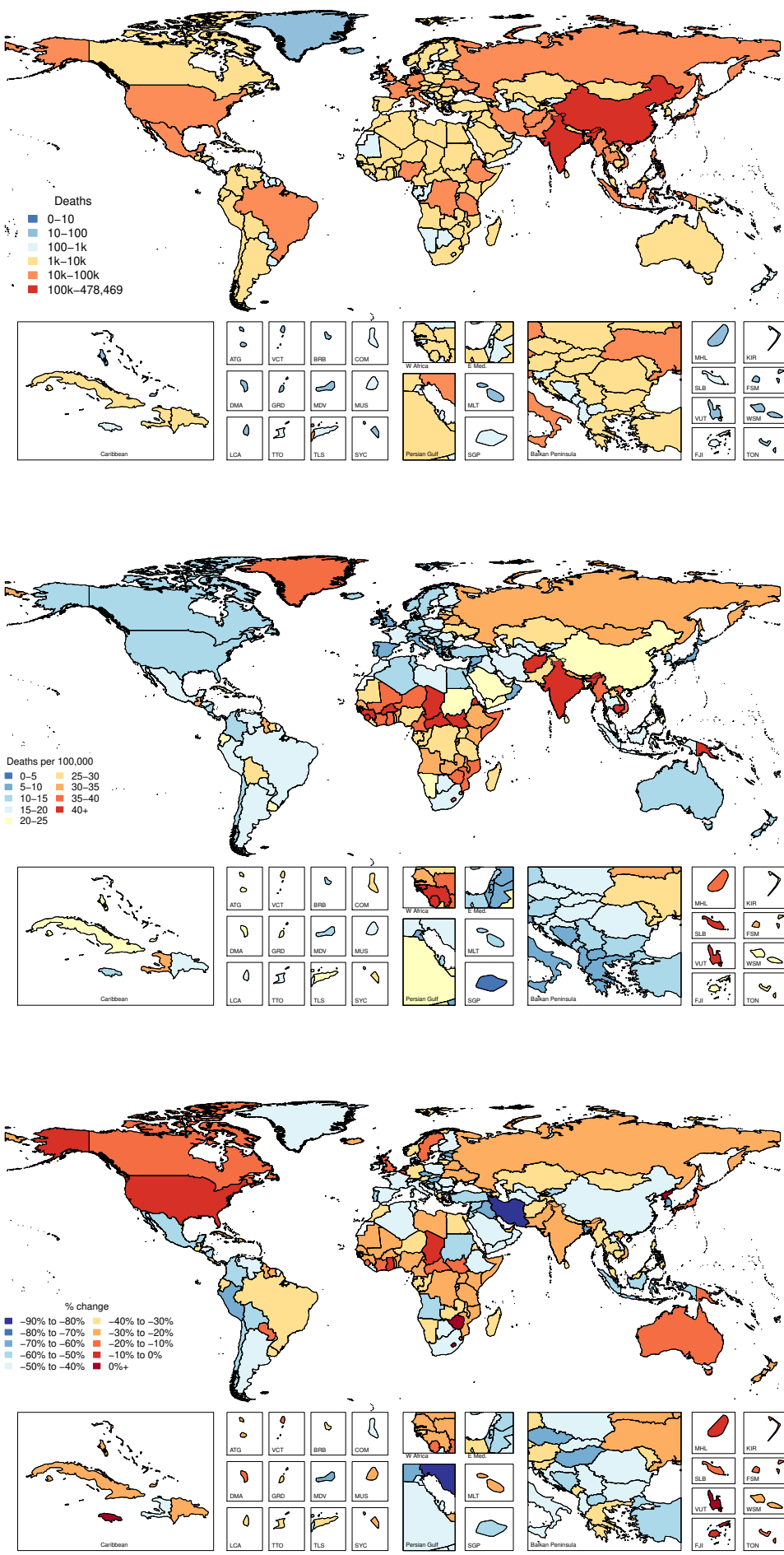

Supplement: Supplementary data [file injuryprev-2019-043494supp009.pdf]

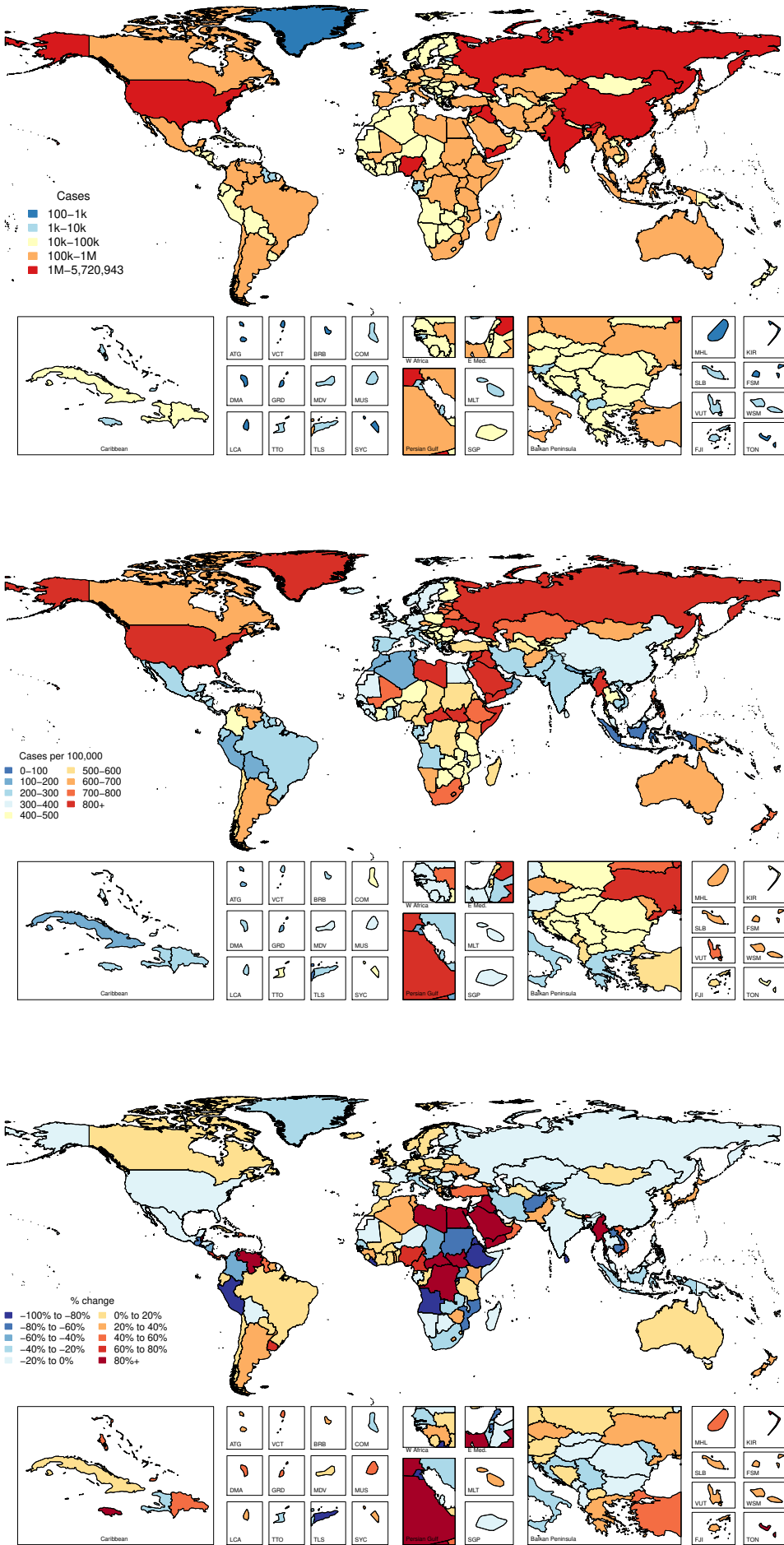

Supplement: Supplementary data [file injuryprev-2019-043494supp010.pdf]

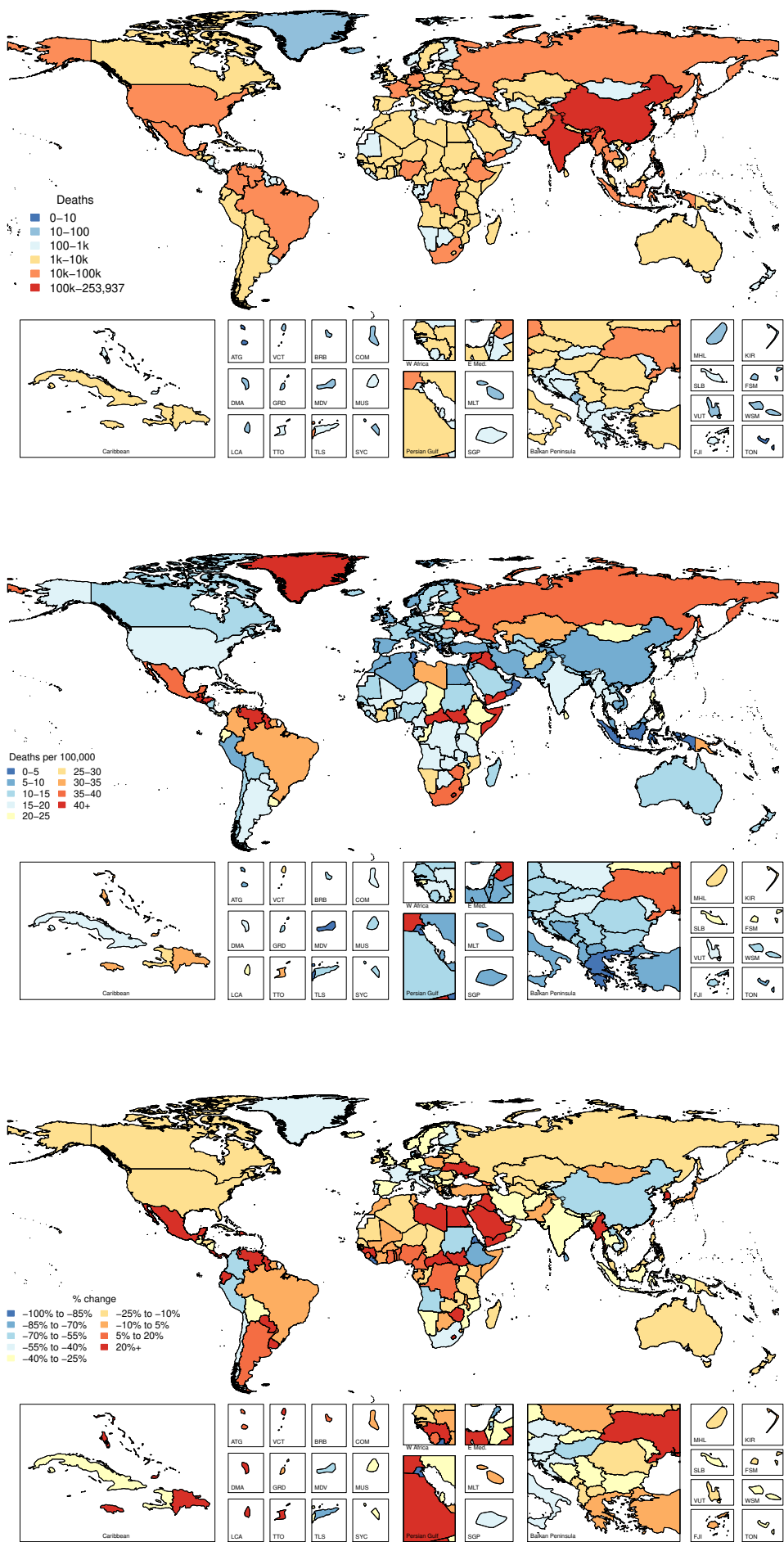

Supplement: Supplementary data [file injuryprev-2019-043494supp011.pdf]
